# Chromosome-level genome and recombination map of the male buffalo

**DOI:** 10.1093/gigascience/giad063

**Published:** 2023-08-17

**Authors:** Xiaobo Wang, Zhipeng Li, Tong Feng, Xier Luo, Lintao Xue, Chonghui Mao, Kuiqing Cui, Hui Li, Jieping Huang, Kongwei Huang, Saif-ur Rehman, Deshun Shi, Dongdong Wu, Jue Ruan, Qingyou Liu

**Affiliations:** Guangdong Provincial Key Laboratory of Animal Molecular Design and Precise Breeding, School of Life Science and Engineering, Foshan University, Foshan 528225, China; State Key Laboratory for Conservation and Utilization of Subtropical Agro-Bioresources, Guangxi University, Nanning 530005, China; Shenzhen Branch, Guangdong Laboratory of Lingnan Modern Agriculture, Genome Analysis Laboratory of the Ministry of Agriculture and Rural Affairs, Agricultural Genomics Institute at Shenzhen, Chinese Academy of Agricultural Sciences, Shenzhen 518120, China; State Key Laboratory for Conservation and Utilization of Subtropical Agro-Bioresources, Guangxi University, Nanning 530005, China; State Key Laboratory for Conservation and Utilization of Subtropical Agro-Bioresources, Guangxi University, Nanning 530005, China; State Key Laboratory for Conservation and Utilization of Subtropical Agro-Bioresources, Guangxi University, Nanning 530005, China; Reproductive Medical and Genetic Center, The People's Hospital of Guangxi Zhuang Autonomous Region, Nanning, Guangxi 530021, China; Shenzhen Branch, Guangdong Laboratory of Lingnan Modern Agriculture, Genome Analysis Laboratory of the Ministry of Agriculture and Rural Affairs, Agricultural Genomics Institute at Shenzhen, Chinese Academy of Agricultural Sciences, Shenzhen 518120, China; Guangdong Provincial Key Laboratory of Animal Molecular Design and Precise Breeding, School of Life Science and Engineering, Foshan University, Foshan 528225, China; State Key Laboratory for Conservation and Utilization of Subtropical Agro-Bioresources, Guangxi University, Nanning 530005, China; State Key Laboratory for Conservation and Utilization of Subtropical Agro-Bioresources, Guangxi University, Nanning 530005, China; State Key Laboratory for Conservation and Utilization of Subtropical Agro-Bioresources, Guangxi University, Nanning 530005, China; State Key Laboratory for Conservation and Utilization of Subtropical Agro-Bioresources, Guangxi University, Nanning 530005, China; State Key Laboratory for Conservation and Utilization of Subtropical Agro-Bioresources, Guangxi University, Nanning 530005, China; State Key Laboratory for Conservation and Utilization of Subtropical Agro-Bioresources, Guangxi University, Nanning 530005, China; State Key Laboratory of Genetic Resources and Evolution, Kunming Institute of Zoology, Chinese Academy of Sciences, Kunming, Yunnan 650223, China; Shenzhen Branch, Guangdong Laboratory of Lingnan Modern Agriculture, Genome Analysis Laboratory of the Ministry of Agriculture and Rural Affairs, Agricultural Genomics Institute at Shenzhen, Chinese Academy of Agricultural Sciences, Shenzhen 518120, China; Guangdong Provincial Key Laboratory of Animal Molecular Design and Precise Breeding, School of Life Science and Engineering, Foshan University, Foshan 528225, China; State Key Laboratory for Conservation and Utilization of Subtropical Agro-Bioresources, Guangxi University, Nanning 530005, China

**Keywords:** male buffalo, genome, y chromosome, recombination map

## Abstract

**Background:**

The swamp buffalo (*Bubalus bubalis carabanesis*) is an economically important livestock supplying milk, meat, leather, and draft power. Several female buffalo genomes have been available, but the lack of high-quality male genomes hinders studies on chromosome evolution, especially Y, as well as meiotic recombination.

**Results:**

Here, a chromosome-level genome with a contig N50 of 72.2 Mb and a fine-scale recombination map of male buffalo were reported. We found that transposable elements (TEs) and structural variants (SVs) may contribute to buffalo evolution by influencing adjacent gene expression. We further found that the pseudoautosomal region (PAR) of the Y chromosome is subject to stronger purification selection. The meiotic recombination map showed that there were 2 obvious recombination hotspots on chromosome 8, and the genes around them were mainly related to tooth development, which may have helped to enhance the adaption of buffalo to inferior feed. Among several genomic features, TE density has the strongest correlation with recombination rates. Moreover, the TE subfamily, SINE/tRNA, is likely to play a role in driving recombination into SVs.

**Conclusions:**

The male genome and sperm sequencing will facilitate the understanding of the buffalo genomic evolution and functional research.

## Background

For sexually reproducing organisms, meiotic recombination plays a vital role in generating genetic diversity and ensuring segregation of homologous chromosomes. Recombination events tend to be unevenly distributed in many species and frequently occur in small genomic regions termed *recombination hotspots* [1, 2]. Genomic characters like transposable elements (TEs), GC contents, and PRDM9 binding are reported to be associated with recombination frequency and promote the formation of recombination hotspots [3–5]. Hotspots among mammals and even between relative species are poorly conserved, and crossover regions are fast-evolving and possibly facilitate adaptive evolution [6]. Therefore, the study of recombination for each individual is necessary for the further functional and evolutionary research on animals.

The domestic water buffalo is an importantly economic animal resource. The global population size of the buffalo is about 200 million, and they supply milk, meat, leather, and draft power in agricultural production for more than 2 billion people [7, 8]. Water buffaloes feed the largest human population all over the world among domestic animals and are viewed as the most exploitative potential livestock by the Food and Agriculture Organization. Two kinds of water buffalo, including swamp buffalo (*Bubalus bubalis carabanesis*; NCBI:txid346063) and river buffalo (*Bubalus bubalis bubalis*), are classified. Swamp buffaloes are mainly distributed in China and Southeast Asian countries, serving as the primary draft animals for rice growing over thousands of years [9]. Their strong bodies are capable of enduring the heavy work in the field. However, high-quality food is often in short supply in its living environment[10], which may have contributed to the buffalo's higher digestibility of crude protein and fiber [11, 12]. Along with the boost of agricultural mechanization, buffaloes are optimized for meat or milk production [13, 14]. Buffalo meat contains less fat and cholesterol in comparison with beef, suggesting that it can decrease the burden on the cardiovascular system and therefore increase the benefits to human health. Moreover, buffalo meat is effective for the treatment of diabetes described in the Chinese medical classic “The Compendium of Materia Medica” [15].

Although several of female buffalo genomes have been finished [9, 14], the genome of a male buffalo, including the Y chromosome, is absent. Genome assembly of the Y chromosome is a huge challenge because of its massive repeat content, half the sequencing depth due to its haploid nature, and high similarity with some regions of the X chromosome. Furthermore, the absence of a male swamp buffalo genome hinders the detection of sperm meiotic recombination on the Y chromosome and the study of its influencing factors. To solve these problems, we sorted long reads from the Y chromosome by a computational method and assembled them separately to generate a high-quality genome of the male swamp buffalo. We further sequenced 78 single sperms from the same male buffalo to provide the first whole-genome recombination map in buffalo. The high-quality genome, fine-scale recombination map, and subsequent analyses are likely to facilitate the genetic breeding of buffalo and promote the comparative genomics research.

## Results

### Genome assembly, evaluation, and annotation

Many mammalian genome projects prioritize sequencing female individuals (XX) over males (XY), as the haploid nature of the Y chromosome results in half its sequencing depth. This can decrease the assembled contiguity and length of the Y chromosome [16]. Additionally, the high number of repetitive sequences and the similarity to parts of the X chromosome make the Y genome assembly more challenging. Recently, a computational method based on population datasets was developed to sort long reads and generate genome sequences of the male-specific region of the Y chromosome (MSY) [17]. This method was applied to male buffalo, resulting in a total length of 9.3 Mb of buffalo MSY with an N50 value of 1.1 Mb. The remaining reads were further assembled, and all resulting contigs were polished with 170× (∼450 G) short reads. Compared to the previously published buffalo genomes [9, 14], our assembly exhibited the best continuity with a contig N50 of 72.2 Mb (Table 1).

**Table 1:** Comparison of the genome assemblies of 3 buffaloes. The orthologous gene dataset used for BUSCO evaluation is mammalia_odb10 (v2021-02-19).

		Contig		Scaffold		
	Species	Total length (Mb)	N50 (Mb)	Total length (Mb)	N50 (Mb)	BUSCO
**This study**	**Swamp buffalo (male)**	2,675	72.2	2,675	120.0	95.8%
**Low et al. (2020)**	**River buffalo (female)**	2,654	18.8	2,654	117.2	94.0%
**Luo et al. (2020)**	**Swamp buffalo (female)**	2,609	8.8	2,631	117.3	95.2%
**Luo et al. (2020)**	**River buffalo (female)**	2,626	3.1	2,646	116.1	95.7%

**Figure 1:**
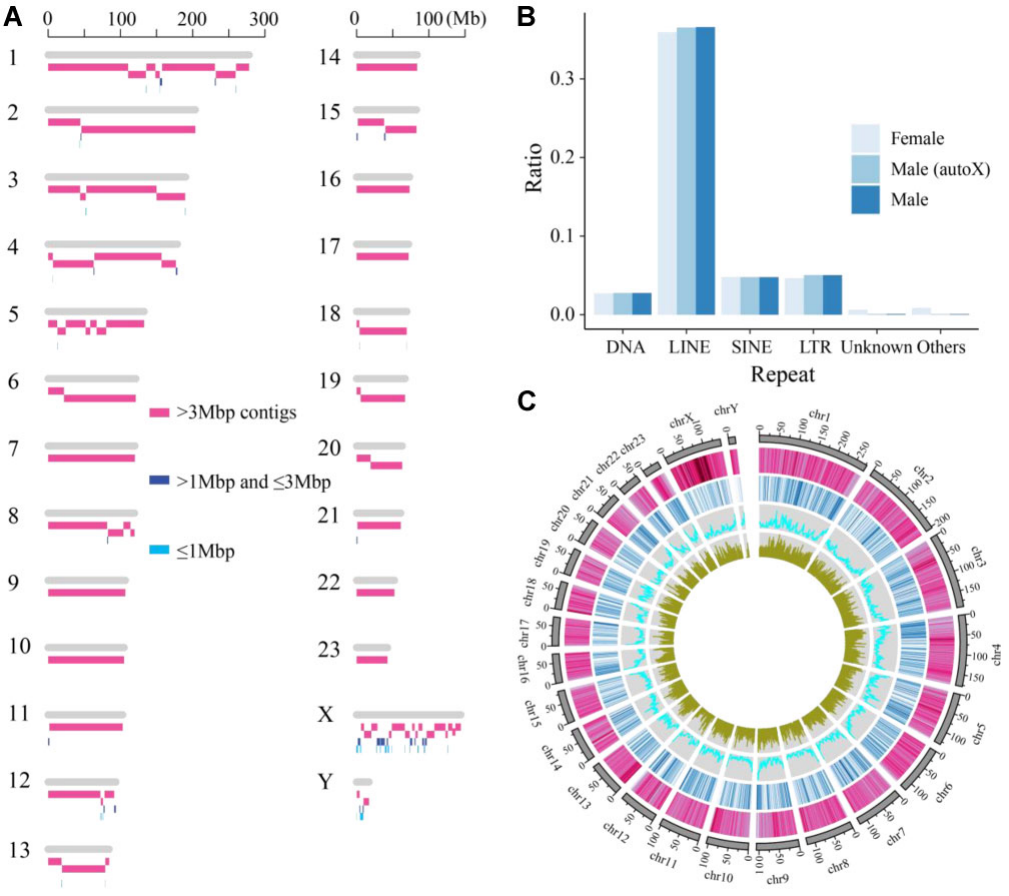
Chromosome-scale assembly of the male swamp buffalo. (A) The distribution of contigs on chromosomes. The assembled results were divided into 3 types of contigs larger than 3 Mb (pink), between 1 Mb and 3 Mb (dark blue), and smaller than 1 Mb (light blue) according to their lengths. (B) Comparison of repetitive content between male and female buffaloes. Male buffalo genome containing only X and autosomes was labeled as “Male (autox).” (C) Circos plot of male buffalo genome. The tracks from outer to inner circles (a–d) indicated the following: chromosomes, TE coverage, gene coverage, GC contents, and gene expression, respectively.

We further sequenced ∼60× Hi-C data to scaffold these contigs. Interestingly, a contig with a length of 7.6 Mb showed a strong interaction signal with both X- and Y- contigs (Supplementary Fig. S1), which is assumed to be the pseudoautosomal region (PAR). The contig was phased by HapCUT2 using short-read, long-read, and Hi-C data. We aligned the 2 haplotypes onto the X chromosome of a female swamp buffalo [9] to determine their locations. Finally, we generated a chromosome-level assembly including 25 long pesudo-chromosomes (N50 = 120.0 Mb) (Fig. 1A,C and Supplementary Fig. S2). Among them, 8 chromosomes consist of only 1 contig (Fig. 1A). Eight chromosomes contain telomeric repeats at one of their ends, and 2 autosomes (Chr3 and Chr5) contain telomeric repeats at both ends. We identified centromeric repeats in 16 chromosomes, and all of them are acrocentric except for chromosomes 1–5, which is consistent with karyotyping analysis [18, 19]. Chromosomes 1–5 are homologous to 2 or 3 cattle chromosomes separately [9, 20], and centromeric repeats are located in all junctions. Based on the comparison with the female swamp buffalo genome [9], our genome closed 287 gaps (65.0 Mb, maximum length is 2.4 Mb) in the female genome (total 532 gaps) (Supplementary Fig. S3). Additionally, we found more transposons, especially LINEs (Long Interspersed Nuclear Elements), that reach several kilobases in length and fewer unknown or other repeats in our assembly (Fig. 1B). All of these results suggest the completeness of our genome assembly of male swamp buffalo.

We further estimated the completeness and accuracy of the final assembly and found that it captured 95.8% of the BUSCO orthologs (Table 1). Using Merqury [21], we obtained a quality value score of 41.3 for our genome assembly. We mapped the short reads of the transcriptome on the genome and found 98.3% of them could be aligned. The homozygous single-nucleotide polymorphism (SNP) ratio was approximately 3.39 × 10^−6^ per base pair based on genomic short-read alignment. Besides, about 92% of the annotated Y genes in the bull genome (Btau_5.0.1) could be explicitly (>90% identity and >95% coverage) mapped to the Y chromosome. To perform genome annotation, we combined 3 methods, including *de novo*, homology-based, and transcriptome-based prediction. In total, we predicted 22,608 protein-coding genes in the male buffalo genome (Supplementary Table S1).

### Evolution of genomic elements

**Figure 2:**
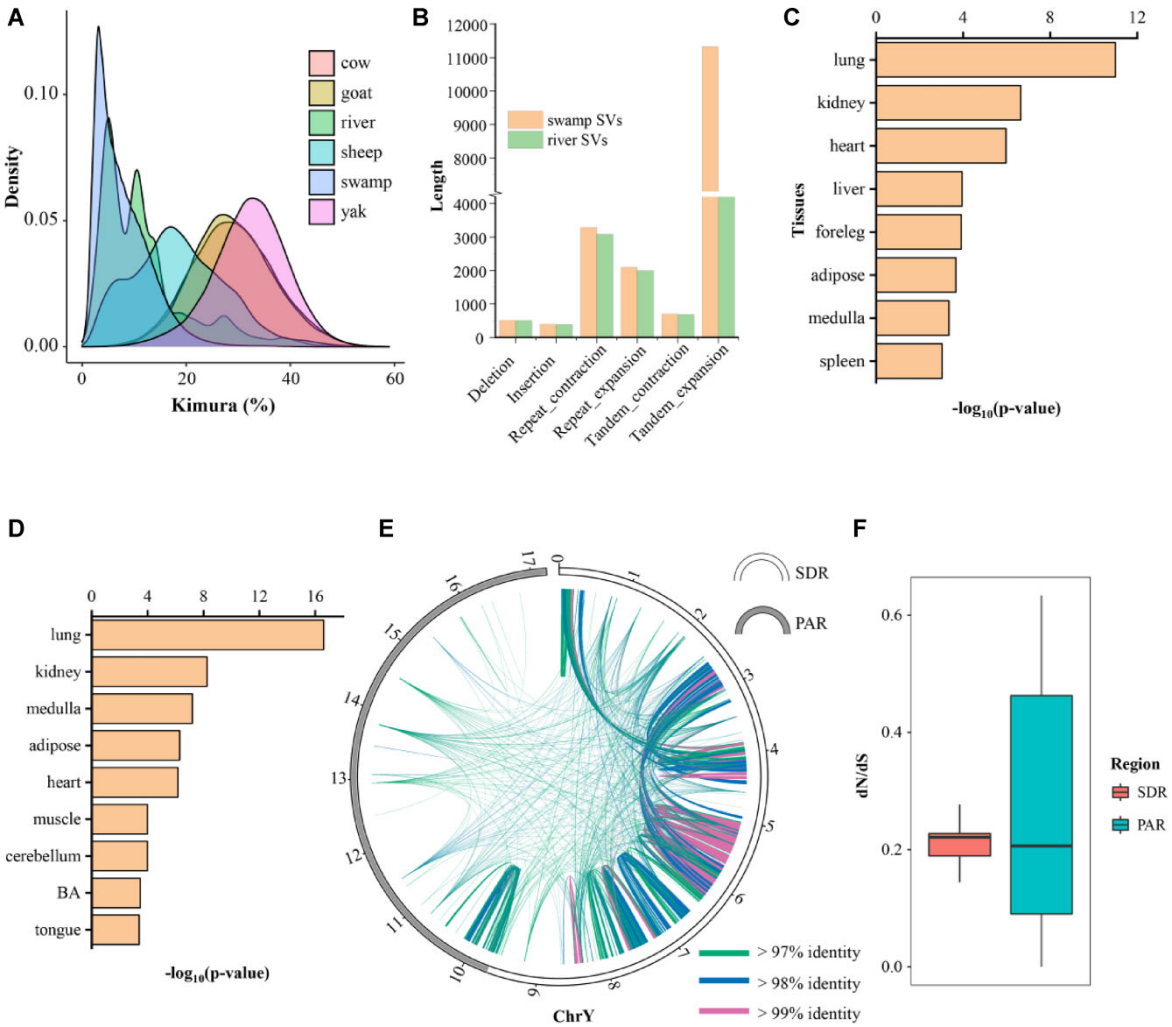
Genomic features of the male buffalo genome. (A) Kimura divergence of TE subfamily LINE/RTE-BovB. The kimura values were calculated by RepeatMasker. (B) Distribution of the SV lengths of male swamp buffalo and river buffalo. (C, D) Tissue distributions of SV-inserted (C) and unique SV-inserted (D) genes with the highest expression levels. Only tissues that are significantly enriched (*P* < 0.05) for genes within SVs compared to all swamp buffalo genes are shown. (E) Intrachromosomal similarities in the Y chromosome of the male buffalo. As shown, line colors represent the minimum identities (only hits >500 bp are plotted). (F) Comparison of the dN/dS values in 2 regions of the Y chromosome.

TEs are ubiquitous in eukaryotic genomes and play a fundamental role in shaping genomic function and evolution [22–27]. In male swamp buffalo, TEs account for approximately half (49.39%) of the genome (Supplementary Table S2). Among them, the LINE/RTE-BovB subclass is the most abundant TE, with a proportion of 17.77%. LINE/RTE-BovB repeats in ruminants are believed to be transferred horizontally from reptiles [28, 29]. We investigated 6 ruminant species with high-quality genomes and found that swamp buffalo LINE/RTE-BovB repeats are more active recently in swamp buffalo than in other species (Fig. 2A). The kimura value of LINE/RTE-BovB burst insertion is 0.03, and the corresponding time is about 1.36 Mya (Million years ago) under a mutation rate of 1.1 × 10^−8^ per generation [30]. This burst time is close to the time when the 2 buffaloes (swamp and river) diverged [9], indicating that it may promote the differentiation of the 2 buffalo species. We discovered that about 14,000 genes of swamp buffalo contained LINE/RTE-BovB repeats in their intronic regions, and LINE/RTE-BovB might be involved in the regulation of many genes, which presumably contributed to the differentiation.

In addition to TEs, structural variants (SVs) offer an alternative approach for genome evolution by influencing gene expression and phenotypes [31–37]. We mapped both swamp and river buffalo to the cattle reference genome (ARS-UCD1.3) and used Assemblytics to detect SVs. We identified a similar number of SVs in both buffalo species (82,877 for swamp and 82,747 for river), of which 63,352 were shared and 19,525 and 19,395 were unique to swamp and river buffalo, separately. The total lengths of SVs were 160.74 Mb and 144.55 Mb in swamp and river buffalo, respectively. Apart from deletions, the average length of all other 5 SV categories (including insertions, repeat expansions, repeat contractions, tandem expansions, and tandem contractions) in swamp buffalo was longer than that in river buffalo (Fig. 2B). To investigate the impact of SVs on genes in swamp buffalo, we studied the expression of genes with SV insertions across diverse tissues. We found that genes with SV insertions tended to have the highest expression levels in the lung tissue (*P* = 1.7E-05) (Fig. 2C). We investigated the condition of swamp buffalo genes with unique SV insertions and still found the same trend (Fig. 2D). Our analysis indicates that SVs in swamp buffalo may have contributed to the development and evolution of the respiratory system.

The genome construction of the Y chromosome provides an opportunity to study the evolution of the sex chromosome in buffalo. It has been reported that mammals' Y chromosome undergoes abundant gene conversion [38], which leads to sequence homogenization [39]. We illustrate the intrachromosomal similarities across the swamp Y chromosome in a circle map (Fig. 2E). It is evident that the sex differentiation region (SDR) sequence is more homogeneous than that of the PAR. Furthermore, we identified paralogous genes within the SDR and between PARs of the X and Y chromosomes and calculated the dN/dS value of these paralogs. The dN/dS value in the PAR was lower than that in the SDR (Fig. 2F), indicating that the PAR was subjected to stronger purification selection against possible gene damage caused by homologous recombination between X and Y chromosomes.

### Identification of recombination events and hotspots

**Figure 3:**
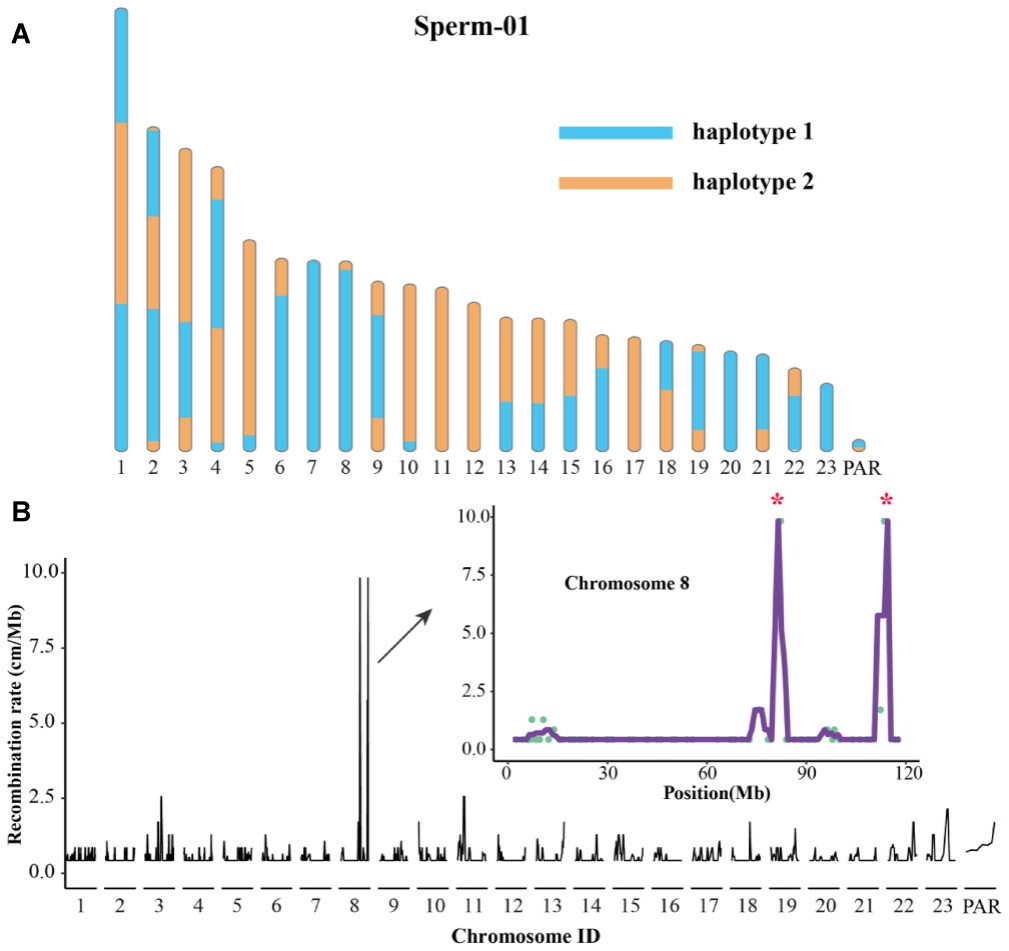
Detecting position of recombination and hotspots. (A) An example of identified recombination maps for the single sperm with ID “Sperm-01.” (B) Distribution of recombination rates across all chromosomes in male swamp buffalo. The distribution of recombination rates on chromosome 8 is amplified. Green circles represent the recombination rate for each bin (3 Mb length), and asterisks represent the locations of recombination hotspots.

To investigate the landscape of recombination events in buffalo, we sequenced 78 sperms from the same male buffalo with an average depth of ∼5×, in total achieving 99.8% genome coverage. By employing a set of stringent filtering measurements and the donor's heterozygous SNP information, we identified a total of 1,934,008 high-confidence SNP loci. Using Hapi [40] software, we inferred chromosome-level haplotypes and identify recombination spots for each sperm (Fig. 3A). In total, we identified 1,956 crossovers with an average of 25.1 per sperm cell, which is similar to that in human studies [41, 42]. Approximately 74.8%, 63.2%, and 42.1% of these crossovers were arranged into the interval of 200, 100, and 30 kb, respectively, indicating a high level of resolution (Supplementary Fig. S4). The distribution of distances between adjacent crossovers was not uniform, with a peak at approximately 50 Mb (Supplementary Fig. S5). Compared to noncrossover regions with a density of 66.3 PRDM9 binding motifs (CCnCCnTnnCCnC) per Mb, we found a higher density of 69.2 binding motifs per Mb around crossovers, indicating a potential role of PRDM9 in regulating meiotic recombination hotspots.

Recombination hotspots are crucial for ensuring the proper segregation of meiotic chromosomes and generating genetic diversity in offspring [43–45]. We calculated the recombination rate with a 3-Mb sliding window and identified 2 distinct recombination hotspots, both located on chromosome 8 (Fig. 3B). These hotspot regions contained 31 genes. By performing functional enrichment analyses in using DAVID [46], we found that the most significant functional term was biomineral tissue development (*P* = 5.6E-4), which included 3 tooth-related genes (IBSP, SPP1, MEPE) (Supplementary Table S3). MEPE, in particular, is thought to be strongly positively selected in herbivorous mammalian lineages and plays a crucial role in promoting the formation and mineralization of dentin, thus contributing to the strength of tooth structure [47]. Notably, buffaloes are known to efficiently utilize coarse feed, such as straw, sunflower cakes, and sprouts, and convert them into valuable animal products [10, 48, 49]. Recombination hotspots may provide genetic diversity to these tooth-related genes, but further experimental validation is required to confirm their functional roles.

### Factors affecting the recombination rate

**Figure 4:**
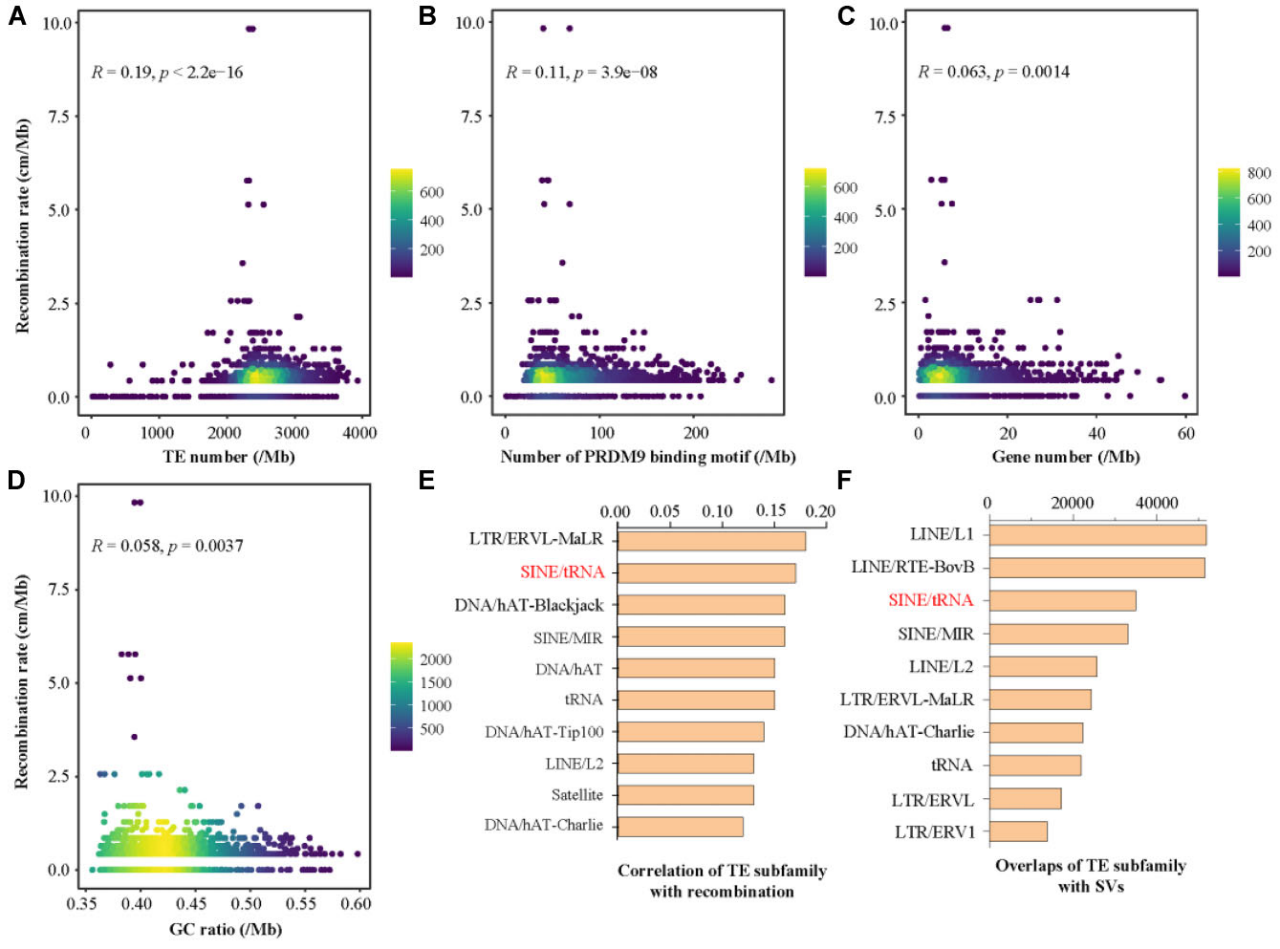
Influencing factors of recombination rate. (A–D) Spearman's rank correlation analysis of recombination rate with various genomic features, including TE density (A), PRDM9 (B), gene density (C), and GC content (D). Each point represents a bin (3 Mb length), and the color represents the number of bins as shown. (E) The top 10 TE subfamilies most associated with recombination rates. (F) The top 10 TE subfamilies contained in SV. The top-ranked SINE/tRNA in both E and F is highlighted in red.

To determine which factor(s) have the greatest impact on recombination rates, several such as PRDM9 binding, TEs, and GC content have been investigated. We performed a correlation analysis between these genomic features and the recombination rates. The effects of density and length were analyzed separately for genes and TEs. We found that gene density and length had almost equal correlations with recombination rates, but for TEs, the density was significantly more correlated than the length (Fig. 4A,C and Supplementary Figs. S6 and S7). Ultimately, among the factors analyzed, TE density was identified as the most influential factor on recombination rates in buffalo (Fig. 4A–D).

Previous studies have reported that TEs are also the main source of SVs [50]. Therefore, it is speculated that TEs may affect the formation of SVs by increasing the frequency of recombination. We further investigated the relationship between TE subfamilies and recombination rates as well as SVs and found that SINE/tRNA had a strong correlation with both recombination rates and SVs (Fig. 4E,F). SINE/tRNA was also found to be an important source of SV in pigs [51]. However, further evidence is needed to validate the functional role of SINE/tRNA in both recombination and SVs of swamp buffalo.

## Discussion

We present here the chromosome-scale genome of male buffalo, which exhibits better contiguity than published buffalo genomes [9, 14]. In addition, we conducted whole-genome sequencing of 78 sperms from the same male buffalo and constructed the first recombination map for buffalo. The high-quality genome, particularly the Y chromosome, and the recombination map provide valuable resources for evolutionary, breeding, and comparative genomic researches of swamp buffalo. Our study could have significant implications for the agricultural sector, particularly in regions where swamp buffalo are an important livestock resource. Our research may also have broader implications for the study of genome evolution and recombination, which can provide insights into the genetic mechanisms that drive species diversification and adaptation. The study has the potential to impact the daily lives of farmers through its contributions to the breeding of water buffaloes for meat and milk production. By identifying genetic variation related to desirable traits and using this information in breeding programs, farmers can improve the productivity and profitability of their herds.

The assembly of the Y chromosome presents a significant challenge due to abundant and lengthy repeats, reduced sequencing depth, and high homology with some regions of the X chromosome [16]. In this study, we overcame these challenges by performing deep long- and ultra-long-read sequencing (∼105×) for the male buffalo. We used the SRY software [17] to sort the long reads of the Y chromosome, and these reads were separately assembled to overcome the last factor. We identified the contig of the PAR through the interaction relationship of the Hi-C heatmap and phased them by combining the second- and third-generation reads and Hi-C data. Finally, we obtained the buffalo Y genome with a total length of 17.2 Mb, which is well mapped by 92% of the annotated genes in the bull Y genome (Btau_5.0.1). The assembly process for the buffalo Y chromosome can also be applied to other animals and plants containing sex-specific chromosomes or fragments.

Meiotic recombination is well studied in model species [5, 41, 42, 52] but less so in livestock. We sequenced 78 buffalo sperms and identified 1,956 recombination events with an average of 25.1 crossovers per sperm cell, which is similar to that of humans [52]. The fine-scale recombination map revealed 2 recombination hotspots on chromosome 8 with significantly higher recombination rates than elsewhere in the swamp buffalo genome. Intriguingly, genes near these hotspots were most significantly related to tooth quality. Given that buffalo's primary food source is low-quality food such as plant straw, recombination hotspots may generate genetic diversities in tooth-associated genes to better adapt to the consumption of crude-fiber diets.

Several factors, such as PRDM9 binding, TEs, and GC content, can influence recombination rates. We found that TE density had the strongest correlation with the recombination rate of swamp-type buffalo. Furthermore, SINE/tRNA, a TE subfamily, was found to have a significant effect on both recombination rate and SVs. We speculate that this SINE/tRNA subfamily may contribute to intraspecies or interspecies genetic variation by promoting recombination. Several studies have shown that the ZnF domain of PRDM9 recognizes specific DNA motifs and is responsible for the formation of recombination hotspots [43, 53–55]. However, the rapid evolution of PRDM9 results in changes in the DNA sequence it binds to [56]. The 13-bp motif (CCnCCnTnnCCnC) in humans that we used may not be optimal for the swamp buffalo PRDM9 binding requirements, which could lead to a weaker effect of the PRDM9 binding sequences on recombination frequency than TEs. Further functional assays are needed to determine the binding motif of swamp buffalo PRDM9. Nevertheless, compared with other factors except for PRDM9 binding, TE density has a relatively high correlation with the recombination rate.

In the future, the genome and recombination map of male river buffalo could be constructed, providing insights into the divergent domestication features between the 2 subspecies of water buffalo and facilitating modern breeding for meat and milk production, as well as identifying genetic variation related to traits of interest. Additionally, further functional assays need to be performed to characterize the binding motif of swamp buffalo PRDM9, which may lead to a better understanding of the factors affecting recombination rates. We plan to continue investigating the genetic basis of important traits in swamp buffalo and to explore ways to use this information to improve breeding programs and animal welfare. We also hope to develop new technologies and methodologies for studying the genetics of nonmodel organisms.

## Method

### Sample collection and sequencing

We sampled blood DNA from a local male buffalo in the Guangxi Zhuang Autonomous Region. To construct a high-quality genome of the male swamp buffalo, several platforms, including Illumina, nanopore, Bionano, and Hi-C, were used to generate a bulk of datasets. Bionano Saphyr technology was applied and DLE1 restriction enzyme was used for digestion. Illumina Hi-C technology was used in this study. For the construction of Hi-C libraries, the buffalo DNA was digested with the restriction enzyme MboI and then was sequenced on an Illumina Novoseq 6000 platform (RRID:SCR_016387) with PE100 reads. We generated about 466.1 Gb (174×) Illumina short reads, 271.9 Gb (102×) nanopore long reads, 561.4 Gb (210×) Bionano molecules, and 291.8 Gb (109×) Hi-C data (Supplementary Table S4). The Hi-C data were used to scaffold the primary genome assembly, and Bionano data were further used to manually check the order and orientation. The sperm was collected at the reproductive medical and genetic Center of the People's Hospital of Guangxi Zhuang Autonomous Region and sequenced according to the previous study [52]. We also sampled 14 tissues, including dorsal muscle, lung, liver, spleen, tongue, kidney, heart, hindleg, foreleg, adipose tissue, conarium, hypothalamus, cerebellum, medulla oblongata, and 7 rough Brodmann areas of the cerebral cortex (BA7/20, BA21/22/41/42, BA23/31/35, BA24/32, BA43, BA11/25, and BA44/45/46) of the buffalo for RNA sequencing on the Illumina Hiseq 2000 platform (RRID:SCR_020132) [57]. The details of sperm and transcriptome data are provided in Supplementary Tables S5 and S6, separately. The cortical divisions are in reference to humans [58].

### Separation of long reads belonging to the Y chromosome

We selected short-read datasets from 59 male swamp buffaloes and 62 female swamp buffaloes from our previous buffalo population study [9]. The datasets and long reads of the reference male buffalo were delivered to the SRY software (v1.5) [17] to identify Y-specific *k*-mers and separated long reads belonging to the Y chromosome.

### Genome assembly

The long reads of the Y chromosome and other chromosomes of the male swamp buffalo were assembled with nextdenovo (v2.4.0) [59], respectively. All of the assembled contigs were polished by nextpolish (v1.3.1) [60] with settings (−max_depth 270 for short-read mapping options and −min_read_len 1k and −max_depth 200 for long-read mapping options) using short reads. We used juicer (v1.5.7) [61] to align Hi-C data onto the male buffalo genome and identified a PAR region candidate contig, ctg000160, that strongly interacts with both X and Y sequences. Then, the extractHAIRS program in HapCUT2 (–indels 1) [62] was used to phase the ctg000160 contig based on the alignments of genomic short reads, nanopore reads, and Hi-C reads. The 2 haplotypes were mapped to the X chromosome sequences of the female swamp buffalo using the mummer software [63], and the more similar one was considered to belong to the PAR of the X chromosome. Finally, we used 3d-dna (v180922) [64] with Hi-C data to anchor the contigs and manually adjust their orders in Juicebox as well as check with Bionano data for generating a chromosome-level genome. The completeness and accuracy of the final assemblies were estimated using BUSCO (RRID:SCR_015008) v5.4.3 [65], Merqury (RRID:SCR_022964) v1.3 [21], and short-read alignment.

### Repeat annotation

We combined *de novo* and homology-based approaches to identify repetitive elements in the male buffalo genome. For the *de novo* approach, we used RepeatModeler (RRID:SCR_015027) v1.0.11 [66] to construct a *de novo* repeat library with default parameters. Then, RepeatMasker (RRID:SCR_012954) (v4.0.9) [66] was run on the male buffalo genome using the *de novo* library. RepeatMasker was also run against RepBase (RRID:SCR_021169) (v20181026) [66] for homologous repeat identification. The results of repeat annotation from the 2 approaches were integrated. TRF (v4.09) [67] with parameters “1 1 2 80 5 200 2000” was used to detect tandem repeats and search 6-mer vertebrate telomeric repeats (TTAGGG or alternative types, including CCCTAA, TAGGGT, ACCCTA, AGGGTT, AACCCT, GGGTTA, TAACCC, GGTTAG, CTAACC, GTTAGG, and CCTAAC). To identify centromeric regions of the male swamp buffalo, centromeric repeats of river buffalo and cattle [68] were aligned to the genome of male swamp buffalo using BLASR (RRID:SCR_000764) (v5.3.3) [69] with at least 70% identities.

### Gene annotation

Three methods, including *de novo*, homolog-based, and transcriptome-based approaches, were used to predict protein-coding genes of male buffalo. To perform *de novo* predictions, we used Augustus (RRID:SCR_008417) [70], Genscan (RRID:SCR_012902) [71], GlimmerHMM (RRID:SCR_002654) [72], and SNAP (RRID:SCR_007936) [73] in the repeat-masked genome sequences. For the homology-based predictions, we downloaded protein sequences of human, mouse, cow, sheep, and horse from the Ensembl database and cow Y chromosome from NCBI and aligned them to the male buffalo genome using tblastn (e-value <10–5). genBlastA (v1.0.138) [74] was then used to cluster the adjacent high-scoring pairs from the same protein alignments, and exonerate (v2.4.0) [75] was used to identify accurate gene structures. After quality control and filtering, reads from all RNA libraries and the testis transcriptome (NCBI accession: PRJEB25226) were mapped to the male buffalo genome using HISAT (v2.1.0) [76], and StringTie (RRID:SCR_016323) (v2.0.6) [77] was subsequently used to predict gene models. Finally, we combined all predicted genes from the 3 methods with EVidenceModeler (RRID:SCR_014659) (r2012-06-25) [78] and filtered out genes with less than 50% transcriptome coverage to generate high-confidence gene sets.

To obtain gene functional annotation, the SwissProt protein database [79] was searched with blastp (RRID:SCR_001010) (ncbi-blast-2.9.0+) (e-value <10−5). The best hits were used to assign homology-based gene functions. We used DAVID (RRID:SCR_001881) (v6.8) [80] to perform functional analysis for candidate genes under a current background (*Homo sapiens*) with Fisher's exact test.

### Detection of SVs

We utilized the nucmer program in the Mummer package (RRID:SCR_018171) (v4.0.0beta2) [63] to perform genome alignments between male swamp buffalo (or river buffalo) and cattle. The resulting delta file was delivered to Assemblytics (v1.2.1) [81] for calling SVs. We set the parameters of Assemblytics with “10000 50 1000000” corresponding to unique alignment length and minimum and maximum size of SVs, respectively. We applied the chisq.test function in the R package for the gene expression comparison of SV-inserted or unique SV-inserted genes with all genes of the male swamp buffalo. The results are listed in Supplementary Tables S7 and S8. Notably, SVs in this study refer to fixed genomic differences between swamp and river buffalo and cattle and not to variants within a population.

### Calculating dN/dS

To compute the dN/dS value of genes on the Y chromosome, we used blastp (ncbi-blast-2.9.0+) with e-value<1-E05 to generated protein alignments for genes in the PARs of the X and Y chromosomes as well as self-to-self alignments for genes in SDR. Optimal alignments other than to themselves were considered as homologous gene pairs. The yn00 in the PAML package (RRID:SCR_014932) (v4.9) [82] was further used to calculate dN/dS values of paralogs.

### SNP calling

Sequencing short reads for each sperm were mapped onto the male buffalo genome using BWA (v0.7.17-r1188) [83]. Bam files for the same sample were merged using samtools (RRID:SCR_002105) (v1.9) [84]. Duplicate reads were removed using the rmdup command in samtools with default parameters. We used samtools mpileup with settings (-C 50 –min-MQ 30 –min-BQ 30) to call SNPs of all 78 sperms together. Using the samtools mpileup and bcftools [84] filter command (-e “%QUAL<30 || DP<30 || DP>200” -g 5 -G 5), we called SNPs for the male buffalo reference. The genotype of single sperm should be consistent with that of the paternal genome, so we selected heterozygous SNPs of the sperms consistent with the reference heterozygous SNP site for the identification of recombination events. To detect crossover events in PAR, we aligned both X and Y single sperm to the PAR of the Y chromosome to identify biallelic SNPs.

### Identifying recombination events in sperm

To detect recombination events in sperm, the Hapi package [40] in R was used to process the heterozygous SNP results of sperm. We followed the operations recommended by the Hapi software step by step. First, we used the “hapiErrorFilter” function with default parameters to remove the potential genotyping errors of sperms. Second, heterozygous SNPs that were genotypes in at least 10 sperm (*n* = 10) were selected for constructing the high‐quality framework by the “hapiFrameSelection” function, separately. Imputation of missing data was performed by the “hapiImupte” function with settings (nSPT = 3, allowNA = 0). Third, we inferred and proofread draft haplotypes by “hapiPhase” and “hapiCVCluster” functions. Multiple crossovers (cv‐links ≥2) within 1 Mb were filtered. We further adopted a maximum parsimony of recombination (MPR) strategy to eliminate incorrect crossovers by the “hapiBlockMPR” function. Fourth, chromosome-level haplotype assembly was achieved by the “hapiAssemble” function, and the haplotypes located at the end of the chromosome were polished using the “hapiAssembleEnd” function with default parameters. Finally, we identified crossovers in sperm by the “hapiIdentifyCV” function based on haplotypes for each sperm. Notably, some recombination events may not be accurately identified despite strict conditions for the process of sperm genotyping and recombination event identification.

## Supplementary Material

giad063_GIGA-D-22-00319_Original_Submission

giad063_GIGA-D-22-00319_Revision_1

giad063_GIGA-D-22-00319_Revision_2

giad063_Response_to_Reviewer_Comments_Original_Submission

giad063_Response_to_Reviewer_Comments_Revision_1

giad063_Reviewer_1_Report_Original_Submission James Prendergast -- 1/4/2023 Reviewed

giad063_Reviewer_1_Report_Revision_1 James Prendergast -- 4/26/2023 Reviewed

giad063_Reviewer_2_Report_Original_Submission Giovanni Chillemi -- 1/5/2023 Reviewed

giad063_Reviewer_3_Report_Original_Submission Dina El-Khishin -- 1/26/2023 Reviewed

giad063_Supplemental_File

## Data Availability

The genomic sequencing reads were deposited in the Genome Sequence Archive in the National Genomics Data Center, with the accession number CRA007045. Genomic (PRJNA907420) and transcriptomic (PRJNA907420) raw data are also available via the ENA. The genome assembly and gene annotation of the male swamp buffalo were deposited in Figshare [85]. All additional supporting data are available in the *GigaScience* GigaDB database [86].

## Additional Files

**Supplementary Fig. S1**. The interaction between the candidate contig ctg000160 in the PAR region and the contigs of the X and Y chromosomes.

**Supplementary Fig. S2**. The heatmap (resolution: 500 kb) of the male buffalo genome. The increase in interaction signal is represented from yellow to red.

**Supplementary Fig. S3**. The distribution of closed gap lengths (bin size 5 kb).

**Supplementary Fig. S4**. Distribution of the internal sizes.

**Supplementary Fig. S5**. Distribution of distances between adjacent recombinations.

**Supplementary Fig. S6**. Correlation between SV length and recombination rate.

**Supplementary Fig. S7**. Correlation between gene length and recombination rate.

**Supplementary Table S1**. Statistics of predicted protein-coding genes in the male buffalo genome.

**Supplementary Table S2**. Analysis of transposable elements (TEs) in the male buffalo genome.

**Supplementary Table S3**. Functional enrichment of genes around recombination hotspots.

## Abbreviations

BUSCO: Benchmarking Universal Single-Copy Orthologs; Mb: megabase pairs; MPR: maximum parsimony of recombination; MSY: male-specific region of the Y chromosome; NCBI: The National Center for Biotechnology Information; PAR: pseudoautosomal region; SDR: sex differentiation region; SNP: single-nucleotide polymorphism; SV: structural variant; TE: transposable element.
