# Supplementary material for: Chromosome-level genome and recombination map of the male buffalo
Source: Gigascience. 2023 Aug 17;12:giad063. doi: 10.1093/gigascience/giad063 (PMC10433102; doi:10.1093/gigascience/giad063)
Supplement: giad063_GIGA-D-22-00319_Original_Submission [file giad063_giga-d-22-00319_original_submission.pdf]

GigaScience

Chromosome-level genome and recombination map of the male buffalo

--Manuscript Draft--

**Manuscript Number:**	GIGA-D-22-00319
**Full Title:**	Chromosome-level genome and recombination map of the male buffalo
**Article Type:**	Data Note
**Funding Information:**	National Natural Science Foundation of China (U20A2051) — Prof Qingyou Liu<br>National Natural Science Foundation of China (31760648) — Prof Qingyou Liu<br>National Natural Science Foundation of China (31860638) — Prof Qingyou Liu<br>Guangxi Science and Technology Major Project (2021AA20037) — Prof Qingyou Liu
**Abstract:**	Background<br>The swamp buffalo (Bubalus bubalis carabanesis) is an economically important livestock supplying milk, meat, leather and draft power. Several female buffalo genomes have been available, but the lack of high-quality male genomes hinders studies on chromosome evolution, especially Y, as well as meiotic recombination.<br>Results<br>Here, a chromosome-level genome with a contig N50 of 72.2Mb, and a fine-scale recombination map of male buffalo were reported. We found that transposable elements (TEs) and structure variants (SVs) may contribute to buffalo evolution by influencing adjacent gene expressions. We further found that the pseudoautosomal region (PAR) of the Y chromosome is subject to stronger purification selection. The meiotic recombination map showed that there were two obvious recombination hotspots on chromosome 8, and the genes around them were mainly related to tooth development, which helped to enhance the adaption of buffalo to inferior feed. Among several genomic features, TE has the strongest correlation with recombination rates. Moreover, the TE subfamily, SINE/tRNA, is likely to play a role in driving recombination into SVs.<br>Conclusions<br>The male genome and sperm sequencing will facilitate the understanding of the buffalo genomic evolution and functional research.
**Corresponding Author:**	Qingyou Liu<br>Foshan University<br>Foshan, GaungDong CHINA
**Corresponding Author Secondary Information:**	
**Corresponding Author's Institution:**	Foshan University
**Corresponding Author's Secondary Institution:**	
**First Author:**	Qingyou Liu
**First Author Secondary Information:**	
**Order of Authors:**	Qingyou Liu<br>Xiaobo Wang<br>Zhipeng Li<br>Tong Feng<br>Xier Luo

Powered by Editorial Manager® and ProduXion Manager® from Aries Systems Corporation

	Lintao Xue
	Chonghui Mao
	Kuiqing Cui
	Hui Li
	Jieping Huang
	Kongwei Huang
	Saif ur Rehman
	Deshun Shi
	Dongdong Wu
	Jue Ruan
**Order of Authors Secondary Information:**	
**Additional Information:**	
**Question**	**Response**
Are you submitting this manuscript to a special series or article collection?	No
**Experimental design and statistics**<br><br>Full details of the experimental design and statistical methods used should be given in the Methods section, as detailed in our Minimum Standards Reporting Checklist. Information essential to interpreting the data presented should be made available in the figure legends.<br><br>Have you included all the information requested in your manuscript?	Yes
**Resources**<br><br>A description of all resources used, including antibodies, cell lines, animals and software tools, with enough information to allow them to be uniquely identified, should be included in the Methods section. Authors are strongly encouraged to cite Research Resource Identifiers (RRIDs) for antibodies, model organisms and tools, where possible.<br><br>Have you included the information requested as detailed in our Minimum Standards Reporting Checklist?	Yes

**Availability of data and materials**<br><br>All datasets and code on which the conclusions of the paper rely must be either included in your submission or deposited in publicly available repositories (where available and ethically appropriate), referencing such data using a unique identifier in the references and in the "Availability of Data and Materials" section of your manuscript.<br><br>Have you have met the above requirement as detailed in our Minimum Standards Reporting Checklist?	Yes

Powered by Editorial Manager® and ProduXion Manager® from Aries Systems Corporation

Manuscript Click here to access/download;Manuscript;manuscript.docx

Chromosome-level genome and recombination map of the male buffalo

Xiaobo Wang[2,3#], Zhipeng Li[2#], Tong Feng[2#], Xier Luo[2], Lintao Xue[4], Chonghui Mao[3], Kuiqing Cui[1,2], Hui Li[2], Jieping Huang[2], Kongwei Huang[2], Saif-ur Rehman[2], Deshun Shi[2], Dongdong Wu[5], Jue Ruan[3*], Qingyou Liu[1,2*]

1. Guangdong Provincial Key Laboratory of Animal Molecular Design and Precise Breeding, School of Life Science and Engineering, Foshan University, Foshan, 528225, China
2. State Key Laboratory for Conservation and Utilization of Subtropical Agro-Bioresources, Guangxi University, Nanning 530005, China
3. Shenzhen Branch, Guangdong Laboratory of Lingnan Modern Agriculture, Genome Analysis Laboratory of the Ministry of Agriculture and Rural Affairs, Agricultural Genomics Institute at Shenzhen, Chinese Academy of Agricultural Sciences, Shenzhen, China
4. Reproductive Medical and Genetic Center, The People's Hospital of Guangxi Zhuang Autonomous Region, Nanning, Guangxi 530021, China
5. State Key Laboratory of Genetic Resources and Evolution, Kunming Institute of Zoology, Chinese Academy of Sciences, Kunming, Yunnan, China

[#]These authors contributed equally: Xiaobo Wang, Zhipeng Li and Tong Feng

*Correspondence author: Qingyou Liu (qyliu-gene@gxu.edu.cn) and Jue Ruan (ruanjue@cass.cn)

Abstract

Background

The swamp buffalo (*Bubalus bubalis carabanesis*) is an economically important livestock supplying milk, meat, leather and draft power. Several female buffalo genomes have been available, but the lack of high-quality male genomes hinders studies on chromosome evolution, especially Y, as well as meiotic recombination.

Results

Here, a chromosome-level genome with a contig N50 of 72.2Mb, and a fine-scale recombination map of male buffalo were reported. We found that transposable elements (TEs) and structure variants (SVs) may contribute to buffalo evolution by influencing adjacent gene expressions. We further found that the pseudoautosomal region (PAR) of the Y chromosome is subject to stronger purification selection. The meiotic recombination map showed that there were two obvious recombination hotspots on chromosome 8, and the genes around them were mainly related to tooth development, which helped to enhance the adaption of buffalo to inferior feed. Among several genomic features, TE has the strongest correlation with recombination rates. Moreover, the TE subfamily, SINE/tRNA, is likely to play a role in driving recombination into SVs.

Conclusions

The male genome and sperm sequencing will facilitate the understanding of the buffalo genomic evolution and functional research.

Background

For sexually reproducing organisms, meiotic recombination plays a vital role in generating genetic diversity and ensuring segregation of homologous chromosomes. Recombination events tend to be uneven distributed in many species and frequently occur in small genomic regions termed recombination hotspots [1, 2]. Genomic characters like transposable elements (TEs), GC contents and PRDM9 binding are reported to be associated with recombination frequency and promote the formation of recombination hotspots [3-5]. Hotspots among mammalian animals and even between relative species are poorly conserved, and crossover regions are fast-evolving and possibly facilitate adaptive evolution [6]. Therefore, the study of recombination for each individual is necessary for the further functional and evolutionary research on animal.

The domestic water buffalo is an importantly economic animal resource. The global population size of the buffalo is about 200 million, and they supply milk, meat, leather and draft power in agricultural production for more than 2 billion people [7, 8]. Water buffaloes feed the largest human population all over the world among domestic animals, and are viewed as the most exploitative potential livestock by the Food and Agriculture Organization (FAO, http://www.fao.org/faostat/) . Two kinds of water buffalo including swamp buffalo (*Bubalus bubalis carabanesis*) and river buffalo (*Bubalus bubalis bubalis*) are classified. Swamp buffaloes are mainly distribute in china and southeast asian countries, serving as the primary draft animals for rice

growing over thousands of years [9]. Their strong bodies are capable of enduring the heavy work in the field. However, high-quality food is often in short supply in its living environment[10], which may have contributed to the buffalo's higher digestibility of crude protein and fiber [11, 12]. Along with the boost of agricultural mechanization, buffaloes are optimized for meat or milk production[13, 14]. Buffalo meat contains less fat and cholesterol in comparison with beef, suggesting that it can decrease the burden on cardiovascular system and therefore increase the benefits to the human health. Moreover, buffalo meat is effective for the treatment of diabetes described in chinese medical classic “The Compendium of Materia Medica” [15].

Although several of female buffalo genomes have been finished, the genome of male buffalo, especially Y chromosome, is absent. Genome assembly of Y chromosome is a huge challenge because of its massive repeat content, half sequencing depth due to the haploid nature and highly similarity with some regions of X chromosome. Furthermore, the absence of male swamp-buffalo genome hinders the detection of meiotic recombination and the study of its influencing factors. To solve these problems, we sorted long reads of Y chromosome by a computational method and assembled them separately to generate high-quality genome of the male swamp buffalo. We further sequenced 78 single sperms from the same male buffalo to provide the first whole-genome recombination map in buffalo. The high-quality genome, fine-scale recombination map and subsequent analysis are likely to facilitate the genetic breeding of buffalo and promote the comparative genomics research.

Results

Genome assembly, evaluation and annotation

Many mammal genome projects select females (XX) over males (XY) for sequencing, because the haploid nature results to half sequencing depth that can decrease the assembled contiguity and length of Y chromosome [16]. In addition, the large number of repetitive sequences and the high similarity to part of the X chromosome increase the difficulty of the Y genome assembly. Recently, we developed a computational method based on population datasets to sort long reads and generate the genome sequences of male-specific region of Y chromosome (MSY) [17]. We applied this method to male buffalo and obtained a total of 9.3Mb length of buffalo MSY with a N50 value of 1.1Mb. The remaining reads were further assembled and all of the resulting contigs were polished with 170X (~450G) short reads. Compared to the previously published buffalo genomes [9, 14], our assembly exhibited the best continuity with a contig N50 of 72.2Mb (**Table 1**).

We further sequenced ~60X HiC data for scaffolding these contigs. Interestingly, a contig with length of 7.6Mb showed strong interaction signal with both X- and Y-contigs (**Fig. S1**), which is assumed to be pseudoautosomal region (PAR). The contig was phased by HapCUT2 using short-read, long-read and HiC data. We aligned the two haplotypes on the X chromosome of female swamp buffalo to determine their locations. Finally, we generated a chromosome-level assembly including 25 long

pesudo-chromosomes (N50 = 120.0Mb) (**Fig. 1a, c and S2**). Among them, eight chromosomes consist of only one contig (**Fig. 1a**). Eight chromosomes contain telomeric repeats at one of their ends, and two autosomes (Chr3 and Chr5) contain telomeric repeats at both ends. We identified centromeric repeats in 16 chromosomes, and all of them are acrocentric expect for chromosomes 1-5, which is consistent with karyotyping analysis [18]. Chromosomes 1-5 are homologous to two or three cattle chromosomes separately [9], and centromeric repeats located in all the junctions. Based on the comparison with the female swamp buffalo genome [9], our genome closed 287 gap (65.0Mb, max length is 2.4Mb) in female genome (totally 532 gaps) (**Fig. S3**). Additionally, we found more transposons, especially LINEs which reach several kilobases in length, and less unknown or other repeats in our assembly (**Fig. 1b**). All of these results suggest the completeness of our genome assembly of male swamp buffalo.

We further estimated the completeness and accuracy of the final assemblies and found that 95.8% of the BUSCO orthologs were captured (**Table 1**). Using Merqury [19], we obtained the QV scores of 41.3 for our genome assembly.We mapped the short reads of transcriptome on the genome, and found 98.3% of them could be aligned. The homozygous single nucleotide polymorphism (SNP) ratio is approximately 3.39 x 10^{-6} estimated on genomic short-read alignment. Besides, about 92% of the annotated Y genes in bull genome could be explicitly (>90% identity and >95% coverage) mapped on the Y chromosome. We combined three methods, including *de novo*, homology-based and transcriptome-based prediction, to perform genome annotation. In total, we predicted 22,608 protein-coding genes in the male buffalo genome (**Table S1**).

Evolution of genomic elements

TEs is ubiquitous in eukaryotic genomes and play an fundamental role in shaping genomic function and evolution [20]. In male swamp buffalo, TEs account for about half (49.39%) of the genome (**Table S2**). Among them, LINE/RTE-BovB is the most abundant TE subclass with a proportion of 17.77%. LINE/RTE-BovB repeats in ruminants are thought to be transferred horizontally from reptiles [21, 22]. We investigated six ruminant species with high quality genomes, and found that swamp LINE/RTE-BovB repeats are very active recently in swamp buffalo compared to other species (**Fig. 2a**). The kimura value of LINE/RTE-BovB burst insertion is 0.03, and the corresponding time is about 1.36Mya under a mutation rate of 1.1 x10^{-8} per generation [23]. This burst time is close to the time when the two buffaloes (swamp and river) diverged [9], indicating that it may promote the differentiation of the two buffalo species. We discovered that about 14,000 genes of swamp buffalo contained LINE/RTE-BovB repeats in intronic regions, and LINE/RTE-BovB might be involved in the regulation of many genes, which presumably contributed to the differentiation.

In addition to TEs, structural variants (SVs) offers an alternative approach to genome evolution by affecting gene expression and phenotype [24]. We mapped both swamp and river buffalo on cattle reference genome and use Assemblytics to detect SVs. We identified nearly the same number of SVs in both buffalo species (82,877 for

swamp and 82,747 for river), of which 63,352 were shared as well as 19,525 and 19,395 were unique separately. The total lengths of SVs are 160.74Mb and 144.55Mb in swamp and river buffalo, respectively. Besides deletion, the average length of all other five SV categories (including insertions, repeat expansions, repeat contractions, tandem expansions and tandem contractions) in swamp buffalo are longer than that of river buffalo (**Fig. 2b**). To investigate the impact of SVs on genes in swamp buffalo, we studied the expression of genes with SV insertions across diverse tissues. We found that these SV-inserted genes most tended to have the highest expression levels in the tissue (P=1.7E-05) (**Fig. 2c**). We investigated the condition of swamp genes with unique SV insertions, and still found the same pattern (**Fig. 2d**). Our analysis indicates that SVs in swamp buffalo may have contributed to the development and evolution of respiratory system.

The genome construction of the Y chromosome provides an opportunity for us to study sex chromosome evolution in buffalo. It was reported that abundant gene conversion occurred on the Y chromosome of mammals [25], and gene conversion could lead to sequence homogenization [26]. We showed the intrachromosomal similarities across the swamp Y chromosome in a circle map (**Fig. 2e**). Obviously, the sequence of the sex-differentiation region (SDR) is more homogeneous than that of the PAR region. We further identified paralogous genes within SDR and between PARs of X and Y chromosomes, and calculated the dN/dS value of these paralogs. We found that the dN/dS value in PAR was lower than that in SDR (**Fig. 2f**), indicating that the PAR region was subjected to stronger purification selection against possible gene damage caused by homologous recombination between X and Y chromosomes.

Identification of recombination events and hotspots

To investigate the landscape of recombination (crossover) events in buffalo, we sequenced 78 sperms from the same male buffalo with an average depth of ~5X, totally achieving 99.8% genome coverage. Combining a set of stringent filtering measurements and the donor’s heterozygous SNP information, we determined 1,934,008 high-confidence SNP loci in total. All of these SNPs were delivered to Hapi[27] software to infer chromosome-level haplotypes and identify recombination spots for each sperm (**Fig. 3a**). In total, we identified 1,956 crossovers with an average of 25.1 per sperm cell, which is similar to that of human study [28, 29]. About 74.8%, 63.2% and 42.1% of these crossovers could be arranged into the interval of 200, 100, and 30 kb, respectively, indicating that the identified recombination events have a high resolution (**Fig. S4**). The distribution of distances between adjacent crossovers is not uniform and its peaks is about 50Mb (**Fig. S5**). In addition, we identified 16,246 PRDM9 binding motif (CCnCCnTnnCCnC) around crossovers with an average of 69.2 per Mb.

Recombination hotspots play an important role in shaping recombination landscapes and creating genetic variation in offspring [30]. We calculated the recombination rate with a 3Mb sliding window and identified two prominent recombination hotspots, both of which were located on chromosome 8 (**Fig. 3b**). These hotspot regions contained 31 genes. By performing functional enrichment

analyses in DAVID database [31], we found that the most significantly functional terms is biomineral tissue development (*P*=5.6E-4), which included three tooth-related genes (IBSP, SPP1, MEPE) (**Table S3**) . Typically, MEPE is thought to be strongly positively selected in herbivorous mammalian lineages, which promotes the formation and mineralization of dentin and further makes the tooth structure stronger [32]. In fact, buffaloes make good use of coarse feed, such as straw, sunflower cakes and sprouts, and convert them into valuable animal products [10]. These genes related to tooth development around recombination hotspots may have contributed to better dietary adaptations in buffaloes.

Factors affecting the recombination rate

Several factors such as PRDM9 binding, TEs and GC contents have been found to be associate with recombination. To determine which factor or factors had the greatest impact on the recombination rate, we performed a correlation analysis between a series of genomic features and the recombination distribution. For TEs and genes, the effects of their numbers and lengths were analyzed separately. We found that gene numbers and lengths correlated almost equally with recombination distribution, but for TEs, their numbers were significantly more correlated than their lengths (**Fig. 4a, c, S6** and **S7**). Ultimately, the number of TEs, among the factors we analyzed, was identified as the most influential factor on recombination rate of buffalo. (**Fig. 4a-d**).

Previous studies have reported that TE is also the main source of SV [33], so it is speculated that TE may affect the formation of SV by increasing the frequency of recombination. We further investigated the relationship between TE subfamilies and recombination rates as well as SVs, and found that SINE/tRNA had strong correlations with both recombination rates and SVs (**Fig. 4e-f**). SINE/tRNA was also found to be an important source of SV in pigs [34]. However, more evidences are needed to validate the functional role of SINE/tRNA in both recombination and SVs of swamp buffalo.

Discussion

Here we report the chromosome-scale genome of male buffalo, which shows the best contiguity as compared to published buffalo genomes. We also conducted whole-genome sequencing for 78 sperms from the male buffalo and constructed the first recombination map in buffalo. The high-quality genome, especially Y chromosome, and the recombination map provide valuable resources for evolutional, breeding and comparative genomic researches of the buffalo.

Owing to abundant and long repeats, reduced sequencing depth and highly homology to some region of X chromosome, the genome assembly of Y chromosome is a huge challenge [16]. In this study, we performed deep long- and ultra-long read sequencing (~105X) for the male buffalo to overcome the first two factors impacting the Y genome assembly. The SRY software [17] was used to sort long reads of the Y chromosome, and these long reads were separately assembled to overcome the last factor. The PAR of the X and Y chromosomes are regions where recombination occurs,

and the similarity between them is very high, which causes the three generations of high-error sequences to be unable to phase them well. We identified the contig of PAR through the interaction relationship of the HiC heatmap, and phased them by combining the second- and third-generation reads and HiC data. Finally, we obtained the buffalo Y genome with a total length of 17.2 Mb, which is well mapped by 92% of the annotated genes in the bull Y genome. The assembly process of the buffalo Y chromosome can also be applied to other animals, as well as to plants containing sex-specific chromosomes or fragments.

Meiotic recombination is widely reported in model species [5, 28, 29, 35], but less studied in economic livestock. We sequenced 78 buffalo sperms and subsequently identified 1,956 recombination events with an average of 25.1 crossovers per sperm cell, which is analogous to that of human [35]. The fine-scale recombination map showed two recombination hotspots located on chromosome 8 with significantly higher recombination rates than elsewhere in the swamp buffalo genome. Interestingly, genes near these hotspots were most significantly related to tooth quality. Buffalo's food source is mainly low-quality food such as plant straw, so recombination hotspots may create genetic diversities in tooth-associated genes to better adapt to the consumption of crude fiber diets.

There are many factors that affect the recombination distribution, such as PRDM9 binding, TEs, and GC content. In this study, we found that the number of TEs had the strongest correlation with the recombination rate of swamp-type buffalo. The number of SINE/tRNA, a TE subfamily, was further found to have a great effect on both recombination rate and SVs. We speculate that this SINE/tRNA subfamily may drive intra-species or inter-species genetic variation by promoting the occurrence of recombination. Several studies revealed that the ZnF domain of PRDM9 recognized specific DNA motif and was responsible for the formation of recombination hotspots [30, 36-38]. However, the fast evolution of PRDM9 caused changes in the DNA sequence it binds to [39]. The 13bp motif (CCnCCnTnnCCnC) in human we used may not be optimal for the buffalo PRDM9 binding requirements, which may lead to a smaller effect of the PRDM9 binding sequences on recombination frequency than TEs. Further functional assays need to be performed to characterize the binding motif of swamp buffalo PRDM9. Nevertheless, compared with other factors expect for PRDM9 binding, TE has a relatively high correlation with the recombination rate.

In the future, the genome and recombination map of male river buffalo are promising to be constructed. Further comparisons will gain insight into the divergent domestication features between the two sub-species of water buffaloes and facilitate the modern breeding for meat and milk production.

Method

Sample collection and sequencing

We sampled blood DNA from a local male buffalo in Guangxi Zhuang Autonomous Region. To construct a high-quality genome of the male swamp buffalo, several platforms including Illumina, nanopore , Bionano and HiC were used to generate a bulk of datasets. The sperms were collected at reproductive medical and genetic Center of the people's hospital of guangxi zhuang autonomous region and sequenced according to the previous study [35]. We also sampled 14 tissues including dorsal muscle, lung, liver, spleen, tongue, kidney, heart, hind leg, fore leg, adipose tissue, conarium, hypothalamus, cerebellum, medulla oblongata and 7 rough Brodmann areas of cerebral cortex (BA7/20, BA21/22/41/42, BA23/31/35, BA24/32, BA43, BA11/25 and BA44/45/46) of the buffalo for RNA sequencing on Illumina 2000 platform. The cortical divisions are in reference to human (https://www.simplypsychology.org/brodmann-areas.html).

Separation of Long reads belonging to Y chromosome

We selected short-read datasets including 59 male swamp buffaloes and 62 female swamp buffaloes from our previous buffalo population study [9]. The datasets and long reads of the reference male buffalo were delivered to SRY software (v1.5) [17] to identify Y-specific kmers and separated long reads belonging to Y chromosome.

Genome assembly

The long reads of Y chromosome and other chromosomes of the male swamp buffalo were assembled with nextdenovo (v2.4.0) (https://github.com/Nextomics/NextDenovo), respectively. All of the assembling contigs were polished by nextpolish (v1.3.1) [40] with settings (-max_depth 270 for short-read mapping options, and -min_read_len 1k and -max_depth 200 for long-read mapping options) using short reads. We used juicer (v1.5.7) [41] to align HiC data on the male buffalo genome, and identified a PAR region candidate contig, ctg000160, which strongly interacts with both X and Y sequences. Then, the extractHAIRS program in HapCUT2 (--indels 1) [42] was used to phase the ctg000160 contig based on the alignments of genomic short reads, nanopore reads and HiC reads. The two haplotypes were mapped to the X chromosome sequences of the female swamp buffalo using mummer software [43], and the more similar one was considered to belong to the PAR of the X chromosome. Finally, we used 3d-dna (v180922) [44] to anchor the contigs and manually adjust their orders in Juicebox for generating a chromosome-level genome. The completeness and accuracy of the final assemblies were estimated using both BUSCO v5.4.3 [45], Merqury (v1.3) [19] and short read alignment.

Repeat annotation

We combined de novo and homology-based approaches to identify repetitive elements in the male buffalo genome. For de novo approach, we used RepeatModeler (v1.0.11)

(http://www.repeatmasker.org/RepeatModeler/) to construct a de novo repeat library with default parameters. Then, RepeatMasker (v4.0.9) (http://www.repeatmasker.org/) was run on the male buffalo genome using the de novo library. RepeatMasker was also run against the RepBase (v20181026) (https://www.girinst.org/repbase/) for homologous repeat identification. The results of repeat annotation from the two approaches were integrated. TRF (v4.09) [46] with parameters "1 1 2 80 5 200 2000" was used to detect tandem repeats and search 6-mer vertebrate telomeric repeats (TTAGGG or alternative types including CCCTAA, TAGGGT, ACCCTA, AGGGTT, AACCCT, GGGTTA, TAACCC, GGTTAG, CTAACC, GTTAGG and CCTAAC). To identify centromeric regions of the male swamp buffalo, centromeric repeats of river buffalo and cattle [47] were aligned to the genome of male swamp buffalo using BLASR (v5.3.3) [48] with at least 70% identities.

Gene annotation

Three methods including de novo, homolog-based and transcriptome-based approaches were used to predict protein-coding genes of male buffalo. To perform de novo predictions, we used Augustus [49], Genscan [50], GlimmerHMM [51] and SNAP [52] in the repeat-masked genome sequences. For the homology-based predictions, we downloaded protein sequences of human, mouse, cow, sheep and horse from the Ensembl database and cow Y chromosome from NCBI, and aligned them to the male buffalo genome using tblastn (e-value < 10-5). genBlastA (v1.0.138) [53] was then used to cluster the adjacent HSPs (high-scoring pairs) from the same protein alignments, and exonerate (v2.4.0) [54] was used to identify accurate gene structures. After QC and filtering, reads from all RNA libraries and testis transcriptome (NCBI accession: PRJEB25226) were mapped to the male buffalo genome using HISAT (v2.1.0) [55], and StringTie (v2.0.6) [56] was subsequently used to predict gene models. Finally, we combined all predicted genes from the three methods with EVidenceModeler (EVM) (r2012-06-25) [57] and filtered out genes with less than 50% transcriptome coverage to generate high-confidence gene sets.

To obtain gene functional annotation, SwissProt protein databases (https://www.uniprot.org/) were searched with blastp (ncbi-blast-2.9.0+) (e-value<10−5). The best hits were used to assign homology-based gene functions. We used DAVID (v6.8) database (https://david.ncifcrf.gov/summary.jsp) to perform functional analysis for candidate genes under a current background (*Homo sapiens*) with the Fisher's test.

Detection of SVs

We utilized nucmer program in Mummer package (v4.0.0beta2) [43] to perform genome alignments between male swamp buffalo (or river buffalo) and cattle. The resulting delta file was delivered to Assemblytics (v1.2.1) [58] for calling SVs. We set the parameters of Assemblytics with "10000 50 1000000" corresponding to unique alignment length, minimum and maximum size of SVs, respectively.

Calculating dN/dS

To compute the dN/dS value of genes in Y chromosome, we used blastp (ncbi-blast-2.9.0+) with e-value<1-E05 to generated protein alignments for genes in PAR of the X and Y as well as self-to-self alignments for genes in SDR. Optimal alignments other than themselves were considered as homologous gene pairs. The yn00 in PAML package (v4.9) [59] was further used to calculate dN/dS values of paralogs.

SNP calling

Sequencing short reads for each sperm were mapped on the male buffalo genome using BWA (v0.7.17-r1188)[60]. Bam files for the same sample were merged using samtools (v1.9) [61]. Duplicate reads were removed using rmdup command in samtools with default parameters. We used samtools mpileup with settings (-C 50 --min-MQ 30 --min-BQ 30) to call SNPs of all 78 sperms together. To acquire high-quality SNP sets, we used bcftools [61] filter command (-e "%QUAL<30 || DP<300 || DP>1800") and view command (-i 'F_missing <0.1' -m2 -M2 -g het -q 0.05 -g 5 -G 5). We further called the heterogenous SNPs for the male buffalo reference, and filtered the sperm SNPs that are not in accordance with heterogenous SNPs. In order to accommodate the biallelic requirements of Hapi software, we aligned both X- and Y-single sperm to the PAR of the Y chromosome to identify SNPs.

Identifying recombination events in sperms

To detect recombination events in sperms, the Hapi package [27] in R was used. Firstly, we used the ‘hapiErrorFilter’ function with default parameters to remove the potential genotyping errors. Secondly, heterozygous SNPs that were genotype in at least 10 gametes (n=10) were selected for constructing the high- quality framework by the ‘hapiFrameSelection’ function, separately. Imputation of missing data was performed by the ‘hapiImupte’ function with settings (nSPT=3, allowNA=0). Thirdly, we inferred and proofread draft haplotypes by ‘hapiPhase’ and ‘hapiCVCluster’ functions. Multiple crossovers (cv- links ≥ 2) within 1 Mb were filtered. We further adopted a Maximum Parsimony of Recombination (MPR) strategy to eliminate incorrect crossovers by the ‘hapiBlockMPR’ function. Fourthly, chromosome-level haplotype assembly was achieved by the ‘hapiAssemble’ function and the haplotypes located at the end of the chromosome were polished using the ‘hapiAssembleEnd’ function with default parameters. Finally, we identified crossovers in sperms by the ‘hapiIdentifyCV’ function based on haplotypes for each sperm.

Tables

	Species	Contig		Scaffold		Busco
		Total length (Mb)	N50 (Mb)	Total length (Mb)	N50 (Mb)	
**This study**	**Swamp buffalo (male)**	2,675	72.2	2,675	120.0	95.8%
**Low et al. (2020)**	**River buffalo (female)**	2,654	18.8	2,654	117.2	94.0%
**Luo et al. (2020)**	**Swamp buffalo (female)**	2,609	8.8	2,631	117.3	95.2%
**Luo et al. (2020)**	**River buffalo (female)**	2,626	3.1	2,646	116.1	95.7%

Table 1. Comparison of the genome assemblies of three buffaloes. The orthologous gene dataset used for busco evaluation is mammalia_odb10 (v2021-02-19).

Figure legend

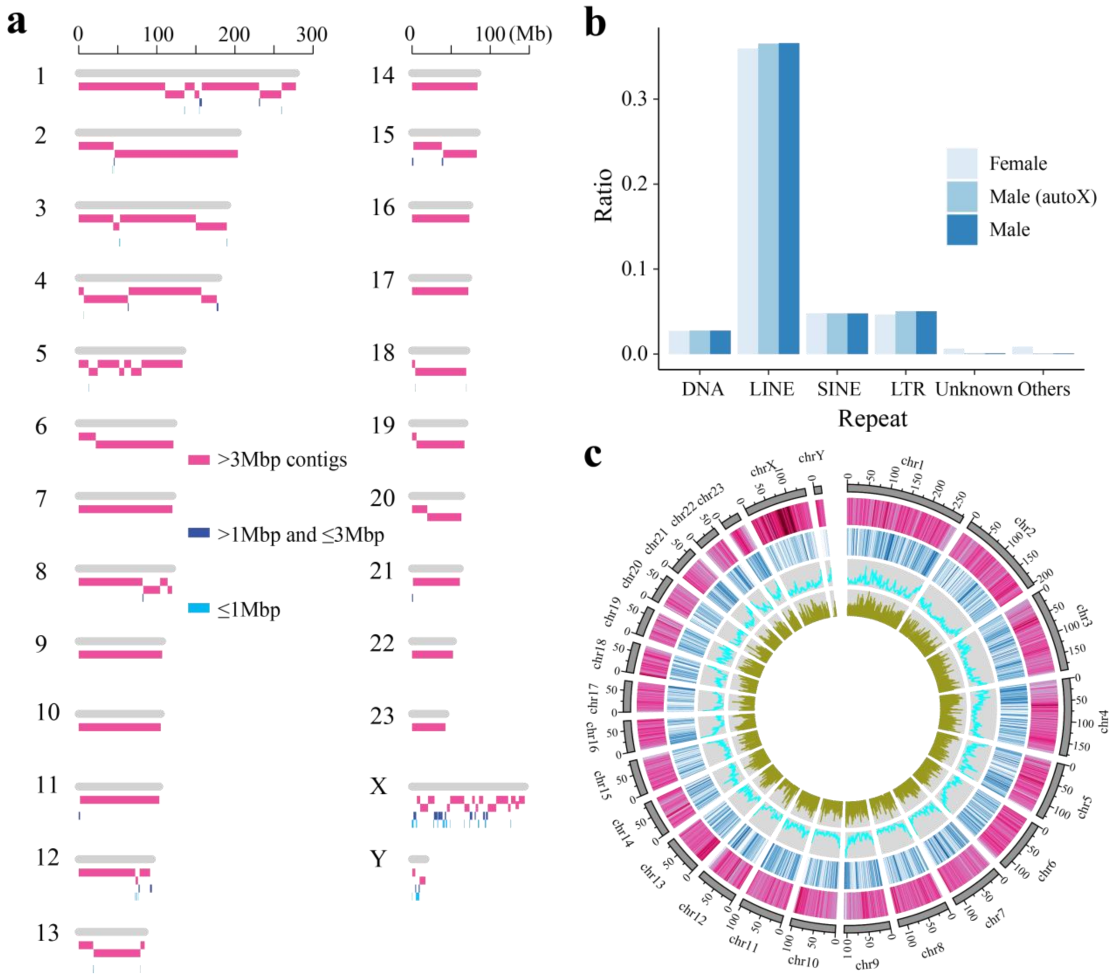

Fig. 1: Chromosome-scale assembly of the male swamp buffalo. a The distribution of contigs on chromosomes. The assembled results were divided into three types of contigs larger than 3Mb (pink), between 1Mb and 3Mb (dark blue) and smaller than 1Mb (light blue) according to their lengths. **b** Comparison of repetitive content between male and female buffaloes. Male buffalo genome containing only X and autosomes was labeled as "Male (autox)". **c** Circos plot of male buffalo genome. The tracks from outer to inner circles (a-d) indicated the following: chromosomes, TE coverage, gene coverage, GC contents, and gene expression, respectively.

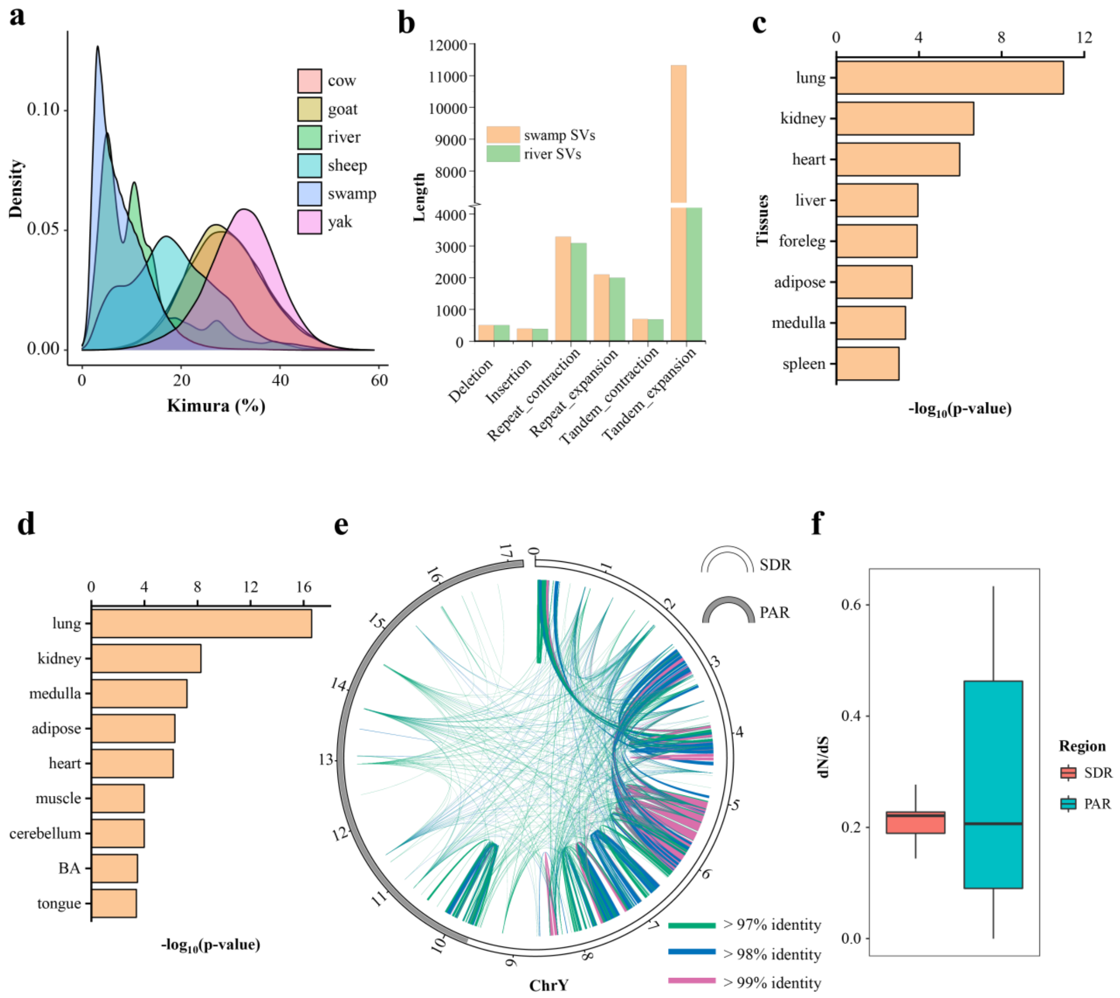

Fig. 2: Genomic features of the male buffalo genome. a Kimura divergence of TE subfamily LINE/RTE-BovB. The kimura values were calculated by RepeatMasker. **b** Distribution of the SV lengths of male swamp buffalo and river buffalo. **c-d** Tissue distributions of SV-inserted (**c**) and unique SV-inserted (**d**) genes with the highest expression levels. Only tissues that are significantly enriched (p-value < 0.05) for genes within SVs compared to all swamp buffalo genes are shown. **e** Intrachromosomal similarities in Y chromosome of the male buffalo. As shown, line colors represent the minimum identities (only hits >500bp are plotted). **f** Comparison of the dN/dS values in two regions of Y chromosome.

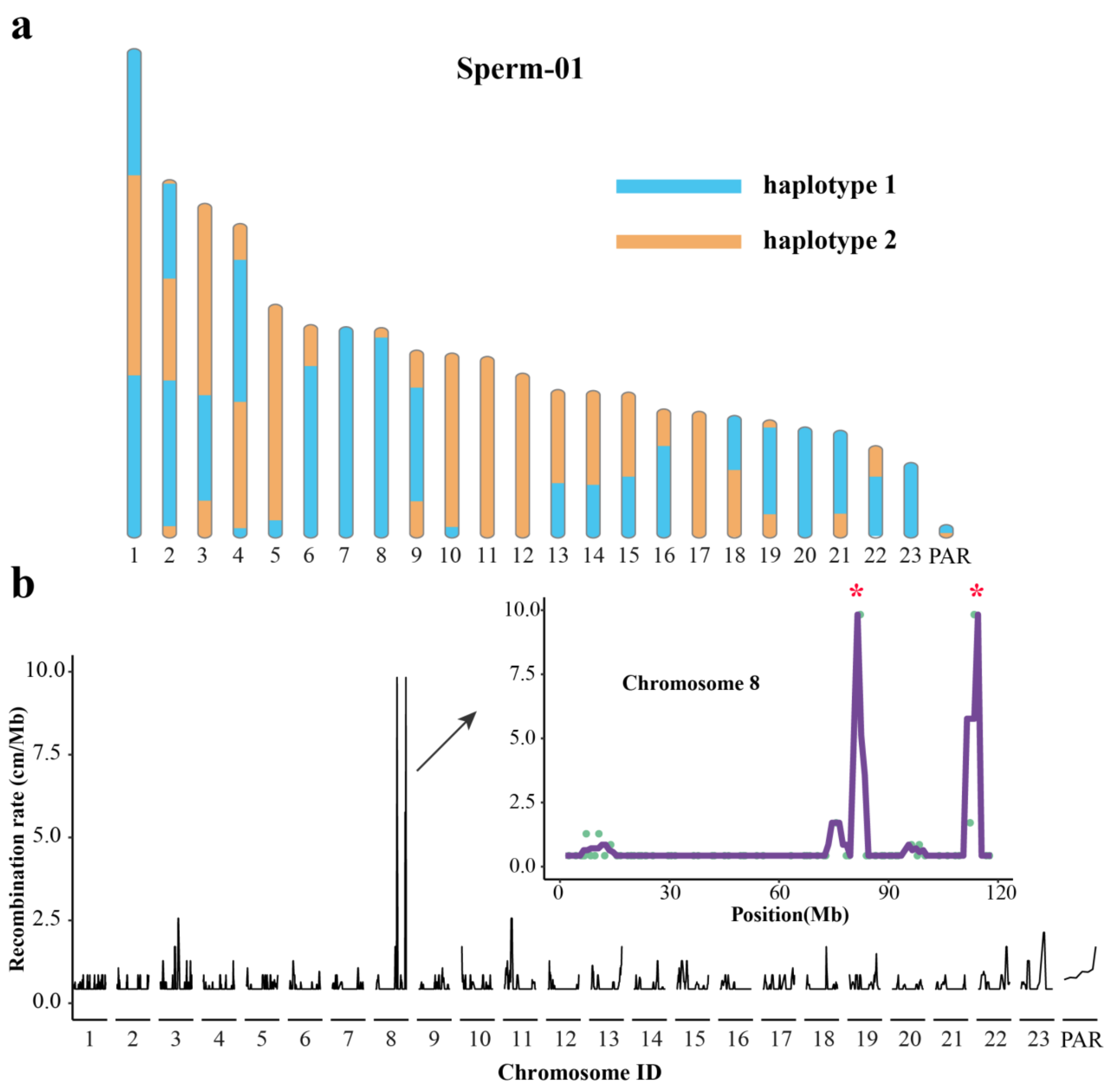

Fig. 3: Detecting position of recombination and hotspots. a An example of identified recombination maps for the single sperm with ID "Sperm-01". **b** Distribution of recombination rates across all chromosomes in male swamp buffalo. The distribution of recombination rates on chromosome 8 is amplified. Green circles represent the recombination rate for each bin (3Mb length), and asterisks represent the locations of recombination hotspots.

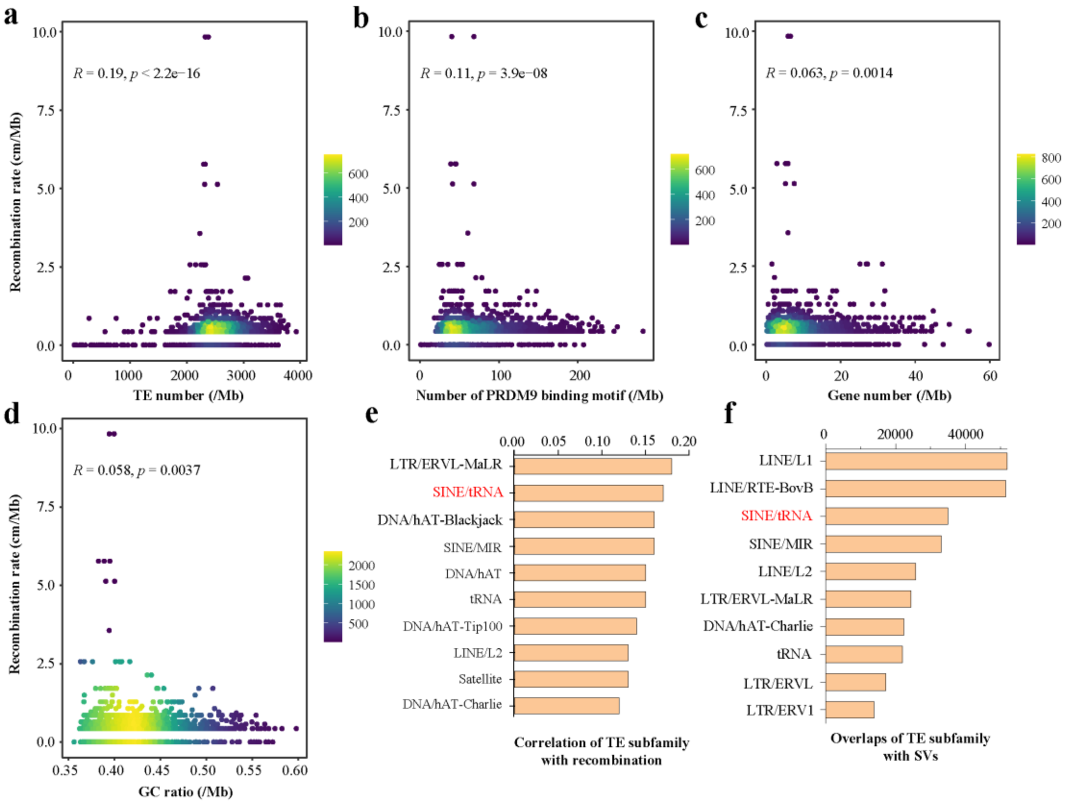

Fig. 4: Influencing factors of recombination rate. a-d Spearman's rank correlation analysis of recombination rate with various genomic features, including TE number (**a**), PRDM9 (**b**), gene number (**c**) and GC content (**d**). Each point represents a bin (3Mb length), and the color represents the number of bins as shown. **e** The top ten TE subfamilies most associated with recombination rates. **f** The top ten TE subfamilies contained in SV. The top-ranked SINE/tRNA in both **e** and **f** is highlighted in red.

Availability of Data and Materials

The genomic sequencing reads were deposited in Genome Sequence Archive in National Genomics Data Center (https://ngdc.cncb.ac.cn/gsa), with the accession number CRA007045. The genome assembly and gene annotation of the male swamp buffalo were deposited in figshare website: https://figshare.com/articles/dataset/Male_swamp_buffalo/19885720.

Additional Files

Supplementary Figure S1. The interaction between the candidate contig ctg000160 in the PAR region and the contigs of the X and Y chromosomes.
Supplementary Figure S2. The heatmap (resolution: 500kb) of the male buffalo genome. The increase in interaction signal is represented from yellow to red color.
Supplementary Figure S3. The distribution of closed gap lengths (bin size 5kb).
Supplementary Figure S4. Distribution of the internal sizes.
Supplementary Figure S5. Distribution of distances between adjacent recombinations.
Supplementary Figure S6. Correlation between SV length and recombination rate.
Supplementary Figure S7. Correlation between gene length and recombination rate.
Supplementary Table S1. Statistics of predicted protein-coding genes in the male buffalo genome.
Supplementary Table S2. Analysis of transposable elements (TEs) in the male buffalo genome.
Supplementary Table S3. Functional enrichment of genes around recombination hotspots.

Abbreviations

BUSCO: Benchmarking Universal Single-Copy Orthologs; Mb: megabase pairs; KEGG: Kyoto Encyclopedia of Genes and Genomes; GO: gene ontology; NCBI: The National Center for Biotechnology Information; QV: quality value.

Competing Interests

The authors declare no competing interests.

Authors' Contributions

Q.L., J.R. and Z.L. designed and leaded the project. X.W. analyzed genome and sperm data and drafted the paper. X.W., T.F., X.L. and R.S. analyzed the transcriptome data. L.X. and K.H. sampled and processed the experimental materials. Q.L. revised the manuscript. K.C., H.L., J.H., C.M., D.W. and D.S. provided suggestions and helped with the checking.

ACKNOWLEDGEMENTS

This research is supported by the National Natural Science Foundation of China

(U20A2051, 31760648 and 31860638), and Guangxi Science and Technology Major Project (2021AA20037).

References

1. Petes and Thomas D. Meiotic recombination hot spots and cold spots. *Nat Rev Genet.* 2001;2(5):360-369.
2. Paigen K and Petkov P. Mammalian recombination hot spots: properties, control and evolution. *Nat Rev Genet.* 2010;11(3):221-233.
3. Capilla L, Caldés MG and Ruiz-Herrera A. Mammalian meiotic recombination: a toolbox for genome evolution. *Cytogenet Genome Res*. 2016;150(1):1-16.
4. Fuentes RR, de Ridder D, van Dijk AD and Peters SA. Domestication shapes recombination patterns in tomato. *Mol Biol Evol*. 2022;39(1):msab287.
5. Hinch AG, Zhang G, Becker PW, Moralli D, Hinch R, Davies B, et al. Factors influencing meiotic recombination revealed by whole-genome sequencing of single sperm. *Science*. 2019;363(6433):eaau8861.
6. Cavassim MIA, Andersen SU, Bataillon T and Schierup M. Recombination facilitates adaptive evolution in rhizobial soil bacteria. bioRxiv. 2021.
7. Scherf BD. *World watch list for domestic animal diversity*. Food and Agriculture Organization (FAO); 2000.
8. Cockrill WR, Fao R and AGA. The husbandry and health of the domestic buffalo. *Trop Anim Health Pro*. 1975;7(1).
9. Luo X, Zhou Y, Zhang B, Zhang Y, Wang X, Feng T, et al. Understanding divergent domestication traits from the whole-genome sequencing of swamp- and river-buffalo populations. *Natl Sci Rev*. 2020;7(3):686-701.
10. Ivanova S and Markov N. Investigation of the feed resource for buffalo. *Acta Scientiarum Animal Sciences*. 2021;43.
11. Ranjhan S. Nutrition of river buffaloes in Southern Asia. *Buffalo Production*. 1992:111-134.
12. O'Brien B and Hennessy D. Scientific appraisal of the Irish grass-based milk production system as a sustainable source of premium quality milk and dairy products. *Irish J Agr Food Res*. 2017;56(1):120-129.
13. Pisano MB, Scano P, Murgia A, Cosentino S and Caboni P. Metabolomics and microbiological profile of Italian mozzarella cheese produced with buffalo and cow milk. *Food Chem*. 2016;192:618-624.
14. Low WY, Tearle R, Bickhart DM, Rosen BD, Kingan SB, Swale T, et al. Chromosome-level assembly of the water buffalo genome surpasses human and goat genomes in sequence contiguity. *Nature Commun*. 2019;10(1):1-11.
15. Li H, Huang K, Wang P, Feng T, Shi D, Cui K, et al. Comparison of long non-coding RNA expression profiles of cattle and buffalo differing in muscle characteristics. *Front Genet*. 2020;11:98.
16. Tomaszkiewicz M, Medvedev P and Makova KD. Y and W Chromosome Assemblies: Approaches and Discoveries. *Trends Genet*. 2017;33(4):266-282. doi:10.1016/j.tig.2017.01.008.

17. Wang X-B, Liu Q-Y, Li A-L and Ruan J. SRY: an effective method for sorting long reads of sex-limited chromosome. *bioRxiv*. 2020.
18. Shaari NAL, Jaoi-Edward M, Loo SS, Salisi MS, Yusoff R, Ab Ghani NI, et al. Karyotypic and mtDNA based characterization of Malaysian water buffalo. *BMC Genet*. 2019;20(1):1-6.
19. Rhie A, Walenz BP, Koren S and Phillippy AM. Merqury: reference-free quality, completeness, and phasing assessment for genome assemblies. *Genome Biol*. 2020;21(1):1-27.
20. Chuong EB, Elde NC and Feschotte C. Regulatory activities of transposable elements: from conflicts to benefits. *Nat Rev Genet*. 2017;18(2):71-86.
21. Kordišs D and Gubenšsek F. Horizontal transfer of non-LTR retrotransposons in vertebrates. *Genetica*. 1999;107(1):121-128.
22. Kordis D and Gubensek F. Unusual horizontal transfer of a long interspersed nuclear element between distant vertebrate classes. *Proc Natl Acad Sci*. 1998;95(18):10704-10709.
23. Roach JC, Glusman G, Smit AF, Huff CD, Hubley R, Shannon PT, et al. Analysis of genetic inheritance in a family quartet by whole-genome sequencing. *Science*. 2010;328(5978):636-639.
24. Kronenberg ZN, Fiddes IT, Gordon D, Murali S, Cantsilieris S, Meyerson OS, et al. High-resolution comparative analysis of great ape genomes. *Science*. 2018;360(6393):eaar6343.
25. Rozen S, Skaletsky H, Marszalek JD, Minx PJ, Cordum HS, Waterston RH, et al. Abundant gene conversion between arms of palindromes in human and ape Y chromosomes. *Nature*. 2003;423(6942):873-876.
26. Hughes JF, Skaletsky H, Pyntikova T, Koutseva N, Raudsepp T, Brown LG, et al. Sequence analysis in Bos taurus reveals pervasiveness of X–Y arms races in mammalian lineages. *Genome Res*. 2020;30(12):1716-1726.
27. Li R, Qu H, Chen J, Wang S, Chater JM, Zhang L, et al. Inference of chromosome-length haplotypes using genomic data of three or a few more single gametes. *Mol Biol Evol*. 2020;37(12):3684-3698.
28. Wang J, Fan HC, Behr B and Quake SR. Genome-wide single-cell analysis of recombination activity and de novo mutation rates in human sperm. *Cell*. 2012;150(2):402-412.
29. Bell AD, Mello CJ, Nemesh J, Brumbaugh SA and Mccarroll SA. Insights into variation in meiosis from 31,228 human sperm genomes. *Nature*. 2020;583(7815):1-6.
30. Parvanov ED, Petkov PM and Paigen K. Prdm9 controls activation of mammalian recombination hotspots. *Science*. 2010;327(5967):835-835.
31. Huang DW, Sherman BT and Lempicki RA. Bioinformatics enrichment tools: paths toward the comprehensive functional analysis of large gene lists. *Nucleic Acids Res*. 2009;37(1):1-13.
32. Mu Y, Tian R, Xiao L, Sun D, Zhang Z, Xu S, et al. Molecular Evolution of Tooth-Related Genes Provides New Insights into Dietary Adaptations of Mammals. *J Mol Evol*. 2021;89(7):458-471.

33. Muotri AR, Marchetto MC, Coufal NG and Gage FH. The necessary junk: new functions for transposable elements. *Hum Mol Genet*. 2007;16(R2):R159-R167.
34. Ai H, Fang X, Yang B, Huang Z, Chen H, Mao L, et al. Adaptation and possible ancient interspecies introgression in pigs identified by whole-genome sequencing. *Nat Genet*. 2015;47(3):217-225.
35. Lu S, Zong C, Fan W, Yang M, Li J, Chapman AR, et al. Probing Meiotic Recombination and Aneuploidy of Single Sperm Cells by Whole-Genome Sequencing. *Science*. 2012;338(6114):1627-1630. doi:10.1126/science.1229112.
36. Myers S, Bowden R, Tumian A, Bontrop RE, Freeman C, MacFie TS, et al. Drive against hotspot motifs in primates implicates the PRDM9 gene in meiotic recombination. *Science*. 2010;327(5967):876-879.
37. Baudat F, Buard J, Grey C, Fledel-Alon A, Ober C, Przeworski M, et al. PRDM9 is a major determinant of meiotic recombination hotspots in humans and mice. *Science*. 2010;327(5967):836-40.
38. Brick K, Smagulova F, Khil P, Camerini-Otero RD and Petukhova GV. Genetic recombination is directed away from functional genomic elements in mice. *Nature*. 2012;485(7400):642-645.
39. Ahlawat S, De S, Sharma P, Sharma R, Arora R, Kataria R, et al. Evolutionary dynamics of meiotic recombination hotspots regulator PRDM9 in bovids. *Mol Genet Genomics*. 2017;292(1):117-131.
40. Hu J, Fan J, Sun Z and Liu S. NextPolish: a fast and efficient genome polishing tool for long-read assembly. *Bioinformatics*. 2020.
41. Durand NC, Shamim MS, Machol I, Rao SS, Huntley MH, Lander ES, et al. Juicer provides a one-click system for analyzing loop-resolution Hi-C experiments. *Cell systems*. 2016;3(1):95-98.
42. Edge P, Bafna V and Bansal V. HapCUT2: robust and accurate haplotype assembly for diverse sequencing technologies. *Genome Res*. 2017;27(5):801-812.
43. Delcher AL, Salzberg SL and Phillippy AMJCpib. Using MUMmer to identify similar regions in large sequence sets. *Curr Protoc Bioinformatics.* 2003;1:10.3.1-10.3.8.
44. Dudchenko O, Batra SS, Omer AD, Nyquist SK, Hoeger M, Durand NC, et al. De novo assembly of the Aedes aegypti genome using Hi-C yields chromosome-length scaffolds. *Science*. 2017;356(6333):92-95.
45. Simao FA, Waterhouse RM, Ioannidis P, Kriventseva EV and Zdobnov EM. BUSCO: assessing genome assembly and annotation completeness with single-copy orthologs. *Bioinformatics*. 2015;31(19):3210-3212. doi:10.1093/bioinformatics/btv351.
46. Benson G. Tandem repeats finder: a program to analyze DNA sequences. *Nucleic Acids Res*. 1999;27(2):573-580.

47. Melters DP, Bradnam KR, Young HA, Telis N, May MR, Ruby JG, et al. Comparative analysis of tandem repeats from hundreds of species reveals unique insights into centromere evolution. *Genome Biol*. 2013;14(1):1-20.
48. Chaisson MJ and Tesler G. Mapping single molecule sequencing reads using basic local alignment with successive refinement (BLASR): application and theory. *BMC Bioinformatics*. 2012;13(1):1-18.
49. Stanke M, Diekhans M, Baertsch R and Haussler D. Using native and syntenically mapped cDNA alignments to improve de novo gene finding. *Bioinformatics*. 2008;24(5):637-644. doi:10.1093/bioinformatics/btn013.
50. Burge C and Karlin S. Prediction of complete gene structures in human genomic DNA. *J Mol Biol*. 1997;268(1):78-94.
51. Majoros WH, Pertea M and Salzberg SL. TigrScan and GlimmerHMM: two open source ab initio eukaryotic gene-finders. *Bioinformatics*. 2004;20(16):2878-2879.
52. Bromberg Y and Rost B. SNAP: predict effect of non-synonymous polymorphisms on function. *Nucleic Acids Res*. 2007;35(11):3823-35.
53. She R, Chu JS, Wang K, Pei J and Chen N. GenBlastA: enabling BLAST to identify homologous gene sequences. *Genome Res*. 2009;19(1):143-149. doi:10.1101/gr.082081.108.
54. Slater GSC and Birney E. Automated generation of heuristics for biological sequence comparison. *BMC Bioinformatics*. 2005;6(1):1-11.
55. Kim D, Paggi JM, Park C, Bennett C and Salzberg SL. Graph-based genome alignment and genotyping with HISAT2 and HISAT-genotype. *Nat Biotechnol*. 2019;37(8):907-915.
56. Pertea M, Pertea GM, Antonescu CM, Chang T-C, Mendell JT and Salzberg SL. StringTie enables improved reconstruction of a transcriptome from RNA-seq reads. *Nat Biotechnol*. 2015;33(3):290-295.
57. Haas BJ, Salzberg SL, Zhu W, Pertea M, Allen JE, Orvis J, et al. Automated eukaryotic gene structure annotation using EVidenceModeler and the Program to Assemble Spliced Alignments. *Genome Biol*. 2008;9(1):R7. doi:10.1186/gb-2008-9-1-r7.
58. Nattestad M and Schatz MC. Assemblytics: a web analytics tool for the detection of variants from an assembly. Bioinformatics. 2016;32 19:3021-3.
59. Yang Z. PAML 4: phylogenetic analysis by maximum likelihood. *Mol Biol Evol*. 2007;24(8):1586-1591. doi:10.1093/molbev/msm088.
60. Li H and Durbin R. Fast and accurate short read alignment with Burrows–Wheeler transform. *Bioinformatics*. 2009;25(14):1754-1760.
61. Li H, Handsaker B, Wysoker A, Fennell T, Ruan J, Homer N, et al. The Sequence Alignment/Map format and SAMtools. *Bioinformatics*. 2009;25(16):2078-2079.

figure1

Click here to access/download;Figure;Figure 1.pdf

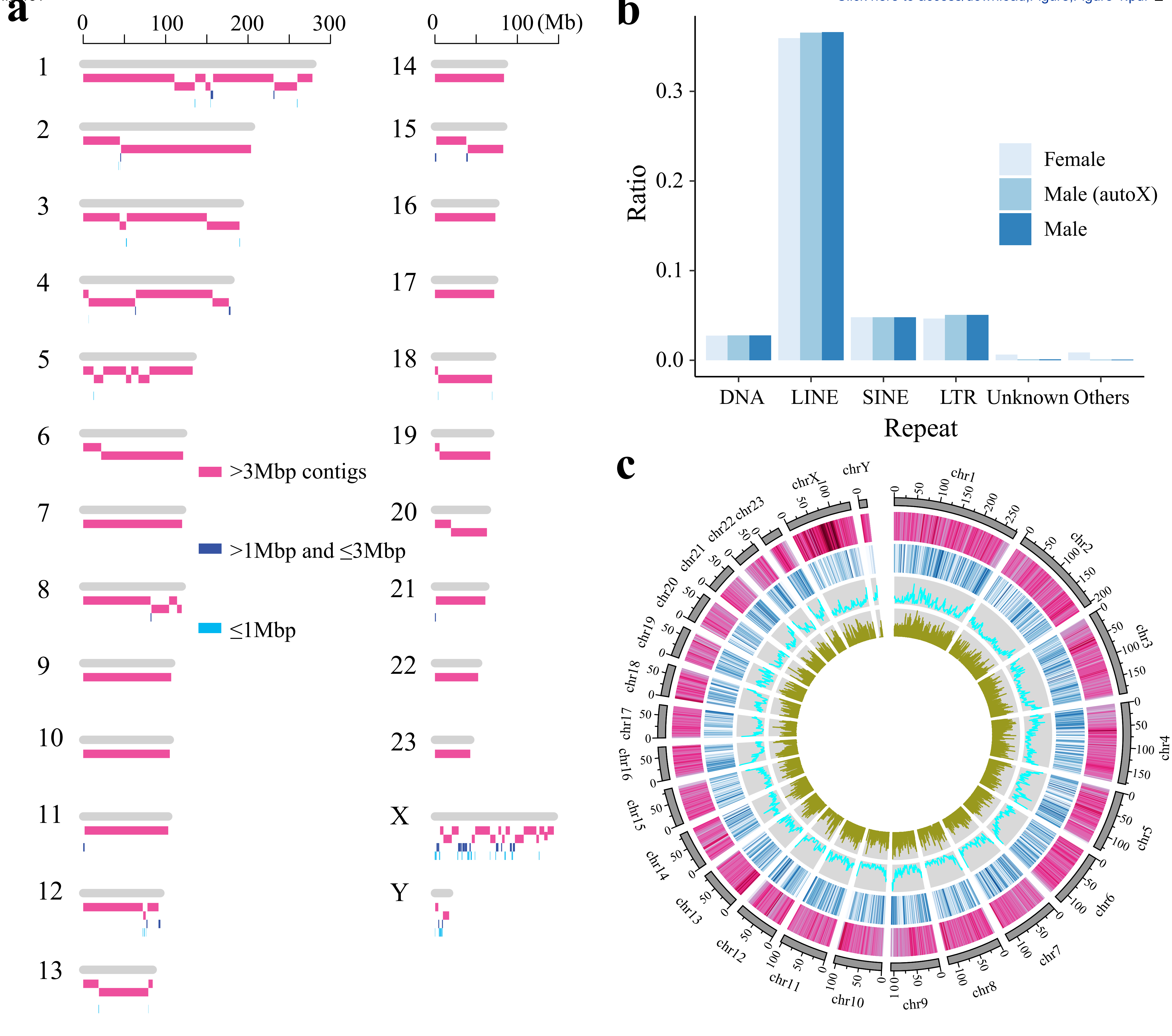

Click here to access/download;Figure;Figure 2.pdf

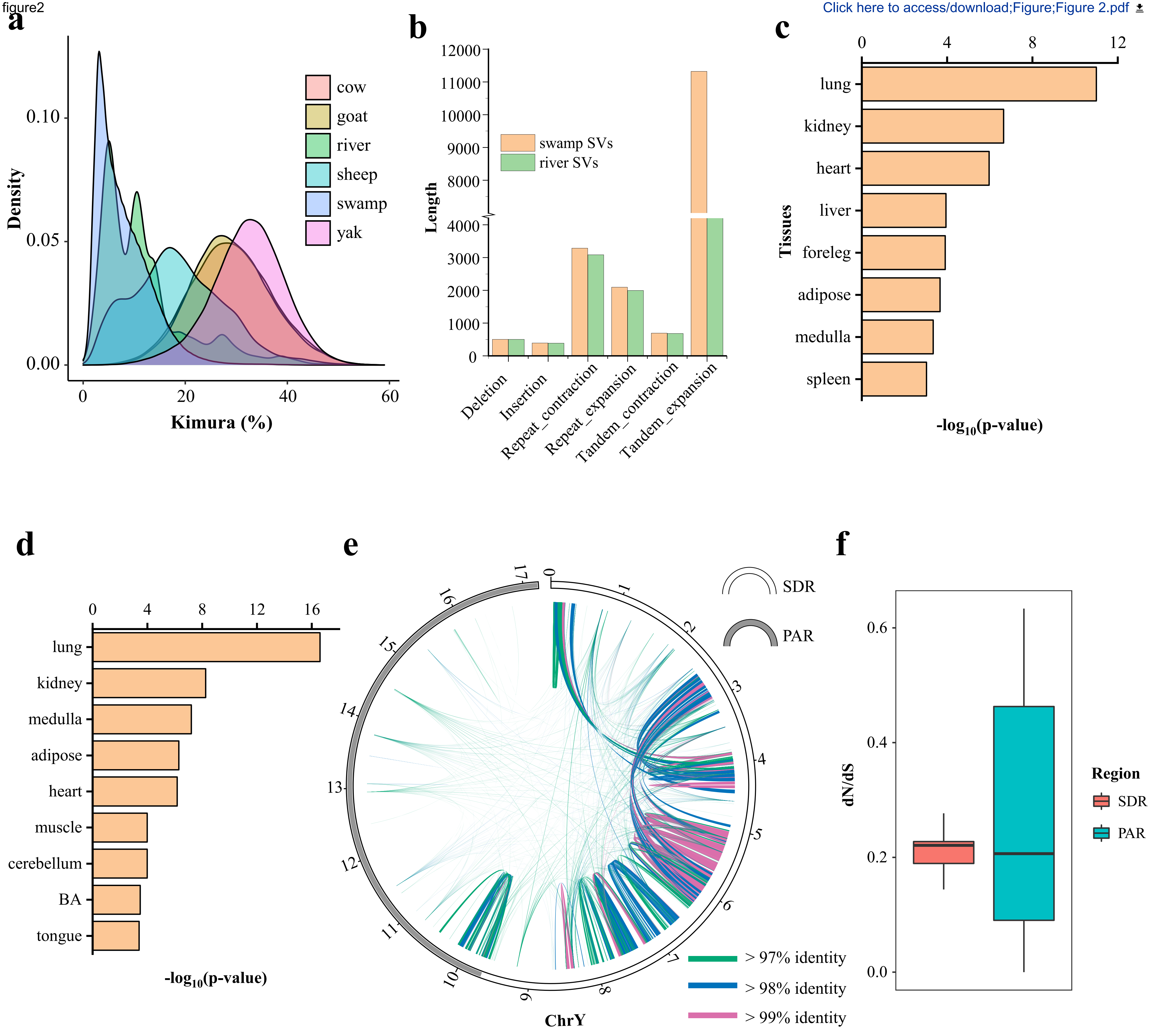

Click here to access/download;Figure;Figure 3.pdf

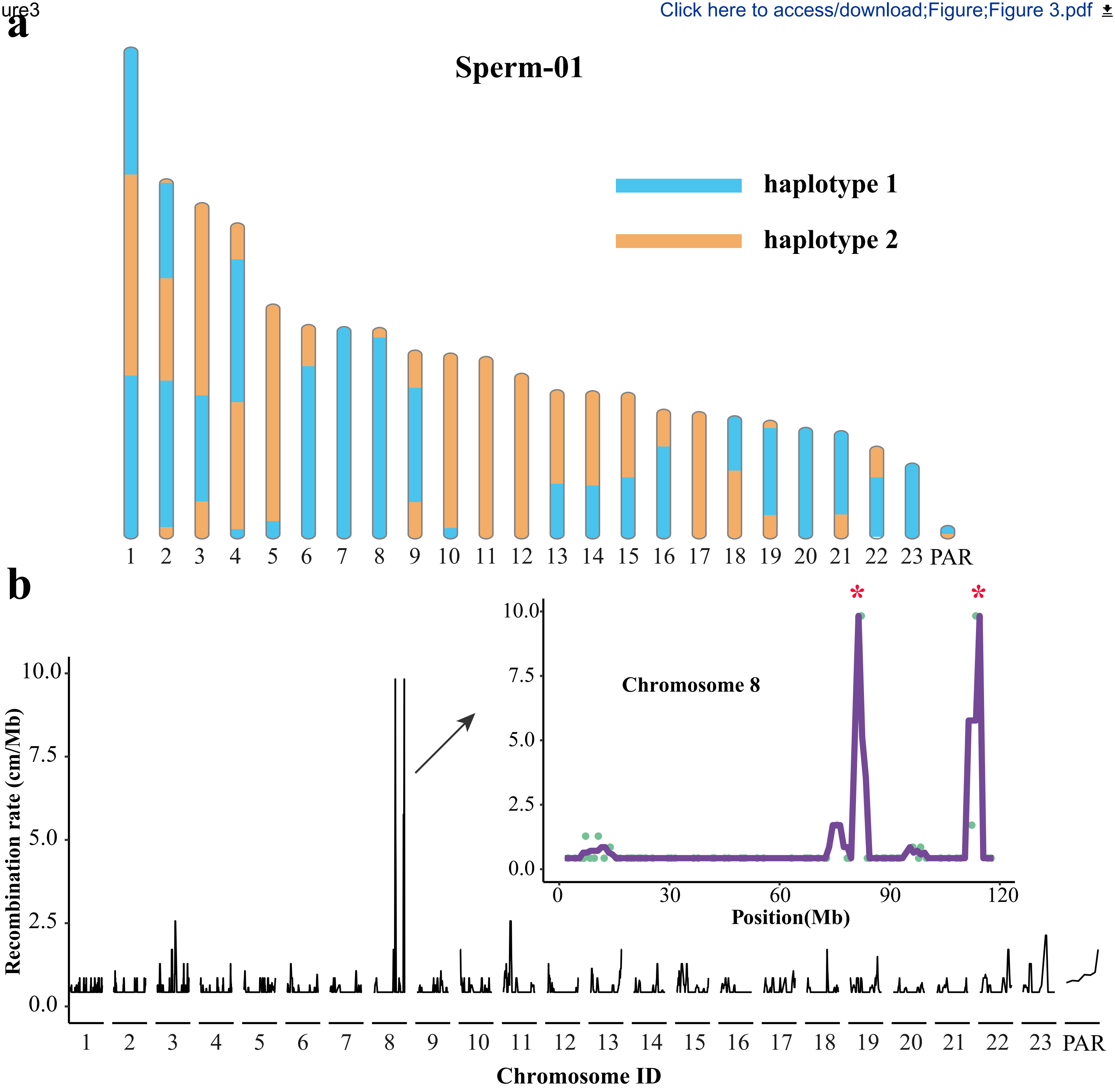

figure4
Click here to access/download;Figure;Figure 4.pdf

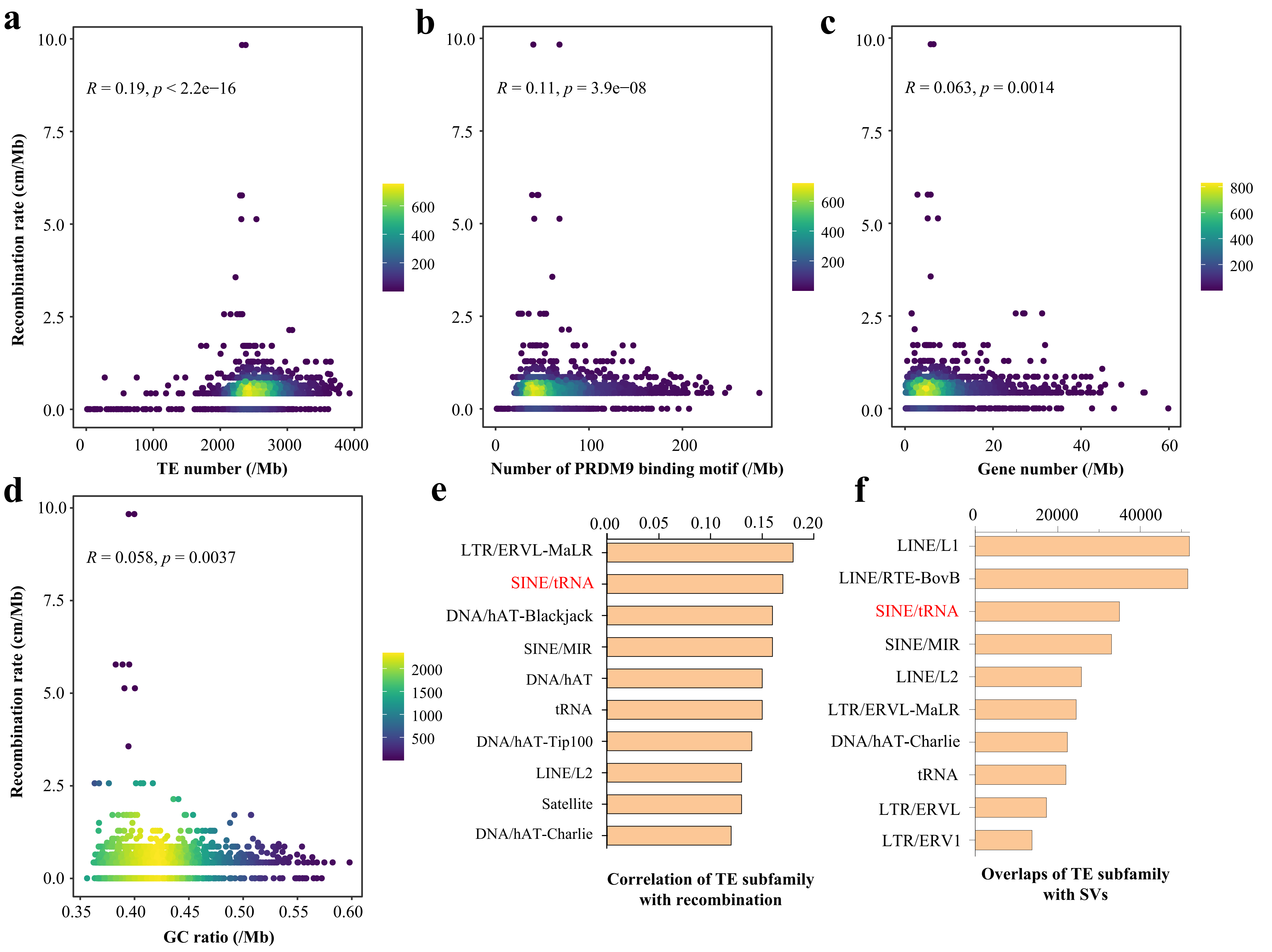

Click here to access/download
Supplementary Material
Supplement.docx

Personal Cover Click here to access/download;Personal Cover;Coverletter.docx

Nov. 24th, 2022

Dear editor,

Please find our manuscript entitled "**Chromosome-level genome and recombination map of the male buffalo**", which we would like to be considered for possible publication in *GigaScience*. We believe the paper may be of particular interest to the readers and will be cited by genomics researchers as well as mammal researchers for both functional genomics and breeding research.

Buffalo is an importantly economic animal resource. The global population size of the buffalo is about 200 million, and they supply milk, meat, leather and draft power in agricultural production for more than 2 billion people. They feed the largest human population all over the world among domestic animals, and are viewed as the most exploitative potential livestock by the FAO (Food and Agriculture Organization). Swamp buffaloes have strong body, and they have been served as the primary draft animals for rice cultivation over thousands of years.

As an important economic livestock, little is known about its Y chromosome and its recombination. While some female buffalo genomes have been published, high-quality male buffalo genomes are still lacking. Here, we generated a high-quality, chromosome-scale genome assembly for a male swamp buffalo, as well as genomic data for 78 sperms of the male buffalo, to understand the evolution of buffalo genomics.

Some of our most exciting findings are: We have assembled the most complete buffalo genome to date (contig N50=72.2Mb), with no gaps in the sequences of eight chromosomes; We provided the first chromosome-level buffalo Y chromosome; transposable elements (TEs), especially the LINE/RTE-BovB subfamily, possibly accelerated the differentiation of swamp- and river- buffalo; The genes inserted with structural variants (SVs) tended to show the highest expression in the lung, suggesting that SVs may provide evolutionary material for the respiratory system of buffalo; The pseudoautosomal regions (PAR) of the Y chromosome were subject to stronger purification selection; There are two obvious meiotic recombination hotspots on chromosome 8, and the genes around them are mainly related to tooth development, which may provide adaptation for crude feed.

In short, the male genome and sperm sequencing provide a very important new resource for understanding the evolution of buffalo. We are convinced that these genomic datasets and the results obtained will be of great interest to a very broad audience.

This paper has not been published or accepted for publication. It is not under consideration at another journal. All of the authors have approved the contents of this

paper and have agreed to the *GigaScience* submission policies.

Thank you for your consideration.

Sincerely,

Qingyou Liu
Corresponding author: Prof. Qingyou Liu, Foshan University;
E-mail: qyliu-gene@gxu.edu.cn
