# Supplementary material for: Chromosome-level genome and recombination map of the male buffalo
Source: Gigascience. 2023 Aug 17;12:giad063. doi: 10.1093/gigascience/giad063 (PMC10433102; doi:10.1093/gigascience/giad063)
Supplement: giad063_GIGA-D-22-00319_Revision_2 [file giad063_giga-d-22-00319_revision_2.pdf]

GigaScience

Chromosome-level genome and recombination map of the male buffalo

--Manuscript Draft--

**Manuscript Number:**	GIGA-D-22-00319R2
**Full Title:**	Chromosome-level genome and recombination map of the male buffalo
**Article Type:**	Data Note
**Funding Information:**	Guangxi Science and Technology Major Project (2021AA20037) — Prof Qingyou Liu<br>Guangxi Science and Technology Major Project (AA22068099) — Prof Qingyou Liu<br>National Natural Science Foundation of China (U20A2051) — Prof Qingyou Liu<br>National Natural Science Foundation of China (31760648) — Prof Qingyou Liu<br>National Natural Science Foundation of China (31860638) — Prof Qingyou Liu
**Abstract:**	Background<br>The swamp buffalo (Bubalus bubalis carabanesis) is an economically important livestock supplying milk, meat, leather and draft power. Several female buffalo genomes have been available, but the lack of high-quality male genomes hinders studies on chromosome evolution, especially Y, as well as meiotic recombination.<br>Results<br>Here, a chromosome-level genome with a contig N50 of 72.2Mb, and a fine-scale recombination map of male buffalo were reported. We found that transposable elements (TEs) and structural variants (SVs) may contribute to buffalo evolution by influencing adjacent gene expression. We further found that the pseudoautosomal region (PAR) of the Y chromosome is subject to stronger purification selection. The meiotic recombination map showed that there were two obvious recombination hotspots on chromosome 8, and the genes around them were mainly related to tooth development, which may have helped to enhance the adaption of buffalo to inferior feed. Among several genomic features, TE density has the strongest correlation with recombination rates. Moreover, the TE subfamily, SINE/tRNA, is likely to play a role in driving recombination into SVs.<br>Conclusions<br>The male genome and sperm sequencing will facilitate the understanding of the buffalo genomic evolution and functional research.
**Corresponding Author:**	Qingyou Liu<br>Foshan University<br>Foshan, GaungDong CHINA
**Corresponding Author Secondary Information:**	
**Corresponding Author's Institution:**	Foshan University
**Corresponding Author's Secondary Institution:**	
**First Author:**	Qingyou Liu
**First Author Secondary Information:**	
**Order of Authors:**	Qingyou Liu<br>Xiaobo Wang<br>Zhipeng Li

Powered by Editorial Manager® and ProduXion Manager® from Aries Systems Corporation

	Tong Feng
	Xier Luo
	Lintao Xue
	Chonghui Mao
	Kuiqing Cui
	Hui Li
	Jieping Huang
	Kongwei Huang
	Saif ur Rehman
	Deshun Shi
	Dongdong Wu
	Jue Ruan
**Order of Authors Secondary Information:**	
**Response to Reviewers:**	Reviewer #1: Thanks to the authors for their response. The manuscript looks good but I think there still are a couple of minor revisions needed. One is there are quite a few English errors so I have attached a tracked version of the manuscript where I have tried to fix/flag some of these in case useful. But think it could likely benefit from some further editing.<br>Response: Thank you for your feedback and suggestions. We have carefully reviewed and revised the manuscript according to your comments. We have also paid close attention to the English language errors and made necessary corrections. We appreciate your help in improving the quality of our manuscript.<br><br>Regarding the details of the sequencing technologies used. It is good to see the details the authors have added. But they still, for example, havnt specified things such as what HiC technology was used (Dovetail?), how was sequenced (Illumina? Read length?). Also for example was it Bionano Saphyr? I think this kind of information should be included in a genome assembly paper.<br>Response: Thank you for your comment. We apologize for any confusion caused by the lack of clarity in our manuscript. Illumina Hi-C technology was used in this study. For the construction of Hi-C libraries, the buffalo DNA was digested with the restriction enzyme MboI and then was sequenced on a Novoseq 6000 platform with PE100 reads. Bionano Saphyr technology was applied and DLE1 restriction enzyme was used for digestion. We have update our manuscript to include this information.<br><br>Also references are missing e.g. at line "Although several of female buffalo genomes have been finished " but they dont cite any. Or for example say "Besides, about 92% of the annotated Y genes in the bull genome could be explicitly..." and "which is well mapped by 92% of the annotated genes in the bull Y genome" but dont specify which bull or which annotations. Or where say "which exhibits better contiguity than published buffalo genomes ". But none cited.<br>Response:Thank you for your valuable feedback on our manuscript. We apologize for the oversight in not including the necessary references in the mentioned sections. We have now revised the manuscript and included the appropriate citations.<br><br>Authors still dont say what this number represents "The homozygous single nucleotide polymorphism (SNP) ratio was approximately 3.39 x 10- 6 based on genomic short-read alignment". From the author response it sounds like this is homozygous variant calls per basepair. If so the authors should specify this (or whatever other unit it is if this is not correct).<br>Response:Yes, the number represents the homozygous variant calls per basepair.We have revised the sentence to read as follows: 'The homozygous single nucleotide polymorphism (SNP) ratio was approximately 3.39 x 10- 6 per basepair based on genomic short-read alignment.'<br><br>"We mapped both swamp and river buffalo to the cattle reference genome and used

	Assemblytics to detect SVs ". If are talking about differences between species I dont think can refer to them as SVs. The term SV is typically used to refer to variants within a species, not across them. (including this is how is defined on wikipedia https://en.wikipedia.org/wiki/Structural_variation). Here seem to be referring to what are likely fixed genomic differences.<br>Response:Thank you for the suggestion. While the term "SV" is typically used to describe variants within a species, some studies do use it to describe genomic differences between different species. For example, Li et.al have aligned the gayal genome to the cattle genome to identify SVs. Therefore, in our study, we have also used the term "SV" to describe the genomic differences between swamp and river buffalo and cattle. However, we appreciate the reviewer's suggestion and will emphasize the point in the Method in our article.<br>Li, Y., Wang, S., Zhang, Z., Luo, J., Lin, G. L., Deng, W. D., et al. (2023). Large-scale chromosomal changes lead to genome-level expression alterations, environmental adaptation, and speciation in the Gayal (Bos frontalis). Molecular Biology and Evolution, 40(1), msad006.<br><br>"In addition, we identified an average of 69.2 PRDM9 binding motif (CCnCCnTnnCCnC) per Mb around crossovers ." How does this compare to regions not around crossovers? Otherwise figure is a bit meaningless.<br>Response: The density of PRDM9 binding motifs in the non-crossover regions is 66.3 per Mb. We have revised the description in the article to "Compared to non-crossover regions with a density of 66.3 PRDM9 binding motifs per Mb, we found a higher density of 69.2 binding motifs per Mb around crossovers, indicating a potential role of PRDM9 in regulating meiotic recombination hotspots."<br><br>"to perform functional analysis for candidate genes under a current background (Homo sapiens) with the Fisher's exact test" should you not restrict the background to genes with an orthologue in buffalo.<br>Response:DAVID requires selecting a species as background for functional enrichment analysis. As human genes have been studied more extensively, we first identified the human orthologs of the buffalo genes and then used human as the background species in DAVID for functional enrichment analysis.<br><br>"We found that gene numbers and lengths had..." think the authors mean gene density, not gene number.<br>Response: Thank you for your comment.We have revised the language to reflect that we are discussing gene density, not gene number. Additionally, we have also changed "TE number" to "TE density" to ensure clarity and accuracy in our analysis. Thank you for bringing this to our attention.
**Additional Information:**	
**Question**	**Response**
Are you submitting this manuscript to a special series or article collection?	No
**Experimental design and statistics**<br><br>Full details of the experimental design and statistical methods used should be given in the Methods section, as detailed in our Minimum Standards Reporting Checklist. Information essential to interpreting the data presented should be made available in the figure legends.<br><br>Have you included all the information	Yes

Powered by Editorial Manager® and ProduXion Manager® from Aries Systems Corporation

requested in your manuscript?	
**Resources**<br><br>A description of all resources used, including antibodies, cell lines, animals and software tools, with enough information to allow them to be uniquely identified, should be included in the Methods section. Authors are strongly encouraged to cite Research Resource Identifiers (RRIDs) for antibodies, model organisms and tools, where possible.<br><br>Have you included the information requested as detailed in our Minimum Standards Reporting Checklist?	Yes
**Availability of data and materials**<br><br>All datasets and code on which the conclusions of the paper rely must be either included in your submission or deposited in publicly available repositories (where available and ethically appropriate), referencing such data using a unique identifier in the references and in the “Availability of Data and Materials” section of your manuscript.<br><br>Have you have met the above requirement as detailed in our Minimum Standards Reporting Checklist?	Yes

Powered by Editorial Manager® and ProduXion Manager® from Aries Systems Corporation

Manuscript
Click here to access/download;Manuscript;manuscript-July 13.docx

Chromosome-level genome and recombination map of the male buffalo

Xiaobo Wang[1,2,3#], Zhipeng Li[2#], Tong Feng[2#], Xier Luo[2], Lintao Xue[4], Chonghui Mao[3], Kuiqing Cui[1,2], Hui Li[2], Jieping Huang[2], Kongwei Huang[2], Saif-ur Rehman[2], Deshun Shi[2], Dongdong Wu[5], Jue Ruan[3*], Qingyou Liu[1,2*]

1. Guangdong Provincial Key Laboratory of Animal Molecular Design and Precise Breeding, School of Life Science and Engineering, Foshan University, Foshan, 528225, China
2. State Key Laboratory for Conservation and Utilization of Subtropical Agro-Bioresources, Guangxi University, Nanning 530005, China
3. Shenzhen Branch, Guangdong Laboratory of Lingnan Modern Agriculture, Genome Analysis Laboratory of the Ministry of Agriculture and Rural Affairs, Agricultural Genomics Institute at Shenzhen, Chinese Academy of Agricultural Sciences, Shenzhen, China
4. Reproductive Medical and Genetic Center, The People's Hospital of Guangxi Zhuang Autonomous Region, Nanning, Guangxi 530021, China
5. State Key Laboratory of Genetic Resources and Evolution, Kunming Institute of Zoology, Chinese Academy of Sciences, Kunming, Yunnan, China
[#]These authors contributed equally: Xiaobo Wang, Zhipeng Li and Tong Feng
*Correspondence author: Qingyou Liu (qyliu-gene@fosu.edu.cn) and Jue Ruan (ruanjue@cass.cn)
Qingyou Liu [0000-0003-3265-540X];
Xiaobo Wang [0000-0001-6754-7404];
Zhipeng Li [0000-0003-3190-2253];
Tong Feng [0000-0002-6056-0590];
Kuiqing Cui [0000-0002-9777-1084];
Saif ur Rehman [0000-0002-5407-3112];
Jue Ruan [0000-0003-3713-3192].

Abstract

Background

The swamp buffalo (*Bubalus bubalis carabanesis*) is an economically important livestock supplying milk, meat, leather and draft power. Several female buffalo genomes have been available, but the lack of high-quality male genomes hinders studies on chromosome evolution, especially Y, as well as meiotic recombination.

Results

Here, a chromosome-level genome with a contig N50 of 72.2Mb, and a fine-scale recombination map of male buffalo were reported. We found that transposable elements (TEs) and structural variants (SVs) may contribute to buffalo evolution by influencing adjacent gene expression. We further found that the pseudoautosomal region (PAR) of

the Y chromosome is subject to stronger purification selection. The meiotic recombination map showed that there were two obvious recombination hotspots on chromosome 8, and the genes around them were mainly related to tooth development, which may have helped to enhance the adaption of buffalo to inferior feed. Among several genomic features, TE density has the strongest correlation with recombination rates. Moreover, the TE subfamily, SINE/tRNA, is likely to play a role in driving recombination into SVs.

Conclusions

The male genome and sperm sequencing will facilitate the understanding of the buffalo genomic evolution and functional research.

Background

For sexually reproducing organisms, meiotic recombination plays a vital role in generating genetic diversity and ensuring segregation of homologous chromosomes. Recombination events tend to be unevenly distributed in many species and frequently occur in small genomic regions termed recombination hotspots [1, 2]. Genomic characters like transposable elements (TEs), GC contents and PRDM9 binding are reported to be associated with recombination frequency and promote the formation of recombination hotspots [3-5]. Hotspots among mammals and even between relative species are poorly conserved, and crossover regions are fast-evolving and possibly facilitate adaptive evolution [6]. Therefore, the study of recombination for each individual is necessary for the further functional and evolutionary research on animal.

The domestic water buffalo is an importantly economic animal resource. The global population size of the buffalo is about 200 million, and they supply milk, meat, leather and draft power in agricultural production for more than 2 billion people [7, 8]. Water buffaloes feed the largest human population all over the world among domestic animals, and are viewed as the most exploitative potential livestock by the Food and Agriculture Organization (FAO). Two kinds of water buffalo including swamp buffalo (*Bubalus bubalis carabanesis*; NCBI:txid346063) and river buffalo (*Bubalus bubalis bubalis*) are classified. Swamp buffaloes are mainly distribute in China and southeast Asian countries, serving as the primary draft animals for rice growing over thousands of years [9]. Their strong bodies are capable of enduring the heavy work in the field. However, high-quality food is often in short supply in its living environment[10], which may have contributed to the buffalo's higher digestibility of crude protein and fiber [11, 12]. Along with the boost of agricultural mechanization, buffaloes are optimized for meat or milk production [13, 14]. Buffalo meat contains less fat and cholesterol in comparison with beef, suggesting that it can decrease the burden on the cardiovascular system and therefore increase the benefits to the human health. Moreover, buffalo meat is effective for the treatment of diabetes described in the Chinese medical classic “The Compendium of Materia Medica” [15].

Although several of female buffalo genomes have been finished [9, 14], the genome of a male buffalo, including the Y chromosome, is absent. Genome assembly of the Y chromosome is a huge challenge because of its massive repeat content, half the sequencing depth due to its haploid nature and highly similarity with some regions of

the X chromosome. Furthermore, the absence of a male swamp-buffalo genome hinders the detection of sperm meiotic recombination on the Y chromosome and the study of its influencing factors. To solve these problems, we sorted long reads from the Y chromosome by a computational method and assembled them separately to generate a high-quality genome of the male swamp buffalo. We further sequenced 78 single sperms from the same male buffalo to provide the first whole-genome recombination map in buffalo. The high-quality genome, fine-scale recombination map and subsequent analyses are likely to facilitate the genetic breeding of buffalo and promote the comparative genomics research.

Results

Genome assembly, evaluation and annotation

Many mammalian genome projects prioritize sequencing female individuals (XX) over males (XY), as the haploid nature of the Y chromosome results in half its sequencing depth. This can decrease the assembled contiguity and length of Y chromosome [16]. Additionally, the high number of repetitive sequences and the similarity to parts of the X chromosome make the Y genome assembly more challenging. Recently, a computational method based on population datasets was developed to sort long reads and generate genome sequences of the male-specific region of Y chromosome (MSY) [17]. This method was applied to male buffalo, resulting in a total length of 9.3Mb of buffalo MSY with an N50 value of 1.1Mb. The remaining reads were further assembled, and all resulting contigs were polished with 170X (~450G) short reads. Compared to the previously published buffalo genomes [9, 14], our assembly exhibited the best continuity with a contig N50 of 72.2Mb (**Table 1**).

We further sequenced ~60X HiC data to scaffold these contigs. Interestingly, a contig with a length of 7.6Mb showed a strong interaction signal with both X- and Y-contigs (**Fig. S1**), which is assumed to be the pseudoautosomal region (PAR). The contig was phased by HapCUT2 using short-read, long-read and HiC data. We aligned the two haplotypes onto the X chromosome of a female swamp buffalo [9] to determine their locations. Finally, we generated a chromosome-level assembly including 25 long pesudo-chromosomes (N50 = 120.0Mb) (**Fig. 1a, c and S2**). Among them, eight chromosomes consist of only one contig (**Fig. 1a**). Eight chromosomes contain telomeric repeats at one of their ends, and two autosomes (Chr3 and Chr5) contain telomeric repeats at both ends. We identified centromeric repeats in 16 chromosomes, and all of them are acrocentric except for chromosomes 1-5, which is consistent with karyotyping analysis [18, 19]. Chromosomes 1-5 are homologous to two or three cattle chromosomes separately [9, 20], and centromeric repeats are located in all the junctions. Based on the comparison with the female swamp buffalo genome [9], our genome closed 287 gaps (65.0Mb, max length is 2.4Mb) in the female genome (total 532 gaps) (**Fig. S3**). Additionally, we found more transposons, especially LINEs that reach several kilobases in length, and fewer unknown or other repeats in our assembly (**Fig. 1b**). All of these results suggest the completeness of our genome assembly of male swamp buffalo.

We further estimated the completeness and accuracy of the final assembly and found that it captured 95.8% of the BUSCO orthologs (**Table 1**). Using Merqury [21], we obtained a QV score of 41.3 for our genome assembly.We mapped the short reads of the transcriptome on the genome, and found 98.3% of them could be aligned. The homozygous single nucleotide polymorphism (SNP) ratio was approximately 3.39 x 10^{-6} per basepair based on genomic short-read alignment. Besides, about 92% of the annotated Y genes in the bull genome (Btau_5.0.1) could be explicitly (>90% identity and >95% coverage) mapped to the Y chromosome. To perform genome annotation, We combined three methods, including *de novo*, homology-based and transcriptome-based prediction. In total, we predicted 22,608 protein-coding genes in the male buffalo genome (**Table S1**).

Evolution of genomic elements

TEs are ubiquitous in eukaryotic genomes and play a fundamental role in shaping genomic function and evolution [22-27]. In male swamp buffalo, TEs account for approximately half (49.39%) of the genome (**Table S2**). Among them, the LINE/RTE-BovB subclass is the most abundant TE, with a proportion of 17.77%. LINE/RTE-BovB repeats in ruminants are believed to be transferred horizontally from reptiles [28, 29]. We investigated six ruminant species with high quality genomes, and found that swamp buffalo LINE/RTE-BovB repeats are more active recently in swamp buffalo than in other species (**Fig. 2a**). The kimura value of LINE/RTE-BovB burst insertion is 0.03, and the corresponding time is about 1.36Mya under a mutation rate of 1.1 x10^{-8} per generation [30]. This burst time is close to the time when the two buffaloes (swamp and river) diverged [9], indicating that it may promote the differentiation of the two buffalo species. We discovered that about 14,000 genes of swamp buffalo contained LINE/RTE-BovB repeats in their intronic regions, and LINE/RTE-BovB might be involved in the regulation of many genes, which presumably contributed to the differentiation.

In addition to TEs, structural variants (SVs) offer an alternative approach for genome evolution by influencing gene expression and phenotypes [31-37]. We mapped both swamp and river buffalo to the cattle reference genome (ARS-UCD1.3) and used Assemblytics to detect SVs. We identified a similar number of SVs in both buffalo species (82,877 for swamp and 82,747 for river), of which 63,352 were shared and 19,525 and 19,395 were unique to swamp and river buffalo, separately. The total lengths of SVs were 160.74Mb and 144.55Mb in swamp and river buffalo, respectively. Apart from deletions, the average length of all other five SV categories (including insertions, repeat expansions, repeat contractions, tandem expansions and tandem contractions) in swamp buffalo was longer than that in river buffalo (**Fig. 2b**). To investigate the impact of SVs on genes in swamp buffalo, we studied the expression of genes with SV insertions across diverse tissues. We found that genes with SV insertions tended to have the highest expression levels in the lung tissue (P=1.7E-05) (**Fig. 2c**). We investigated the condition of swamp buffalo genes with unique SV insertions, and still found the same trend (**Fig. 2d**). Our analysis indicates that SVs in swamp buffalo may have contributed to the development and evolution of the respiratory system.

The genome construction of the Y chromosome provides an opportunity to study the evolution of the sex chromosome in buffalo. It has been reported that mammals' Y chromosome undergoes abundant gene conversion [38], which lead to sequence homogenization [39]. We illustrated the intrachromosomal similarities across the swamp Y chromosome in a circle map (**Fig. 2e**). It is evident that the sex-differentiation region (SDR) sequence is more homogeneous than that of the PAR. Furthermore, We identified paralogous genes within the SDR and between PARs of the X and Y chromosomes, and calculated the dN/dS value of these paralogs. The dN/dS value in the PAR was lower than that in the SDR (**Fig. 2f**), indicating that the PAR was subjected to stronger purification selection against possible gene damage caused by homologous recombination between X and Y chromosomes.

Identification of recombination events and hotspots

To investigate the landscape of recombination events in buffalo, we sequenced 78 sperms from the same male buffalo with an average depth of ~5X, in total achieving 99.8% genome coverage. By employing a set of stringent filtering measurements and the donor's heterozygous SNP information, we identified a total of 1,934,008 high-confidence SNP loci.Using Hapi [40] software, we inferred chromosome-level haplotypes and identify recombination spots for each sperm (**Fig. 3a**). In total, we identified 1,956 crossovers with an average of 25.1 per sperm cell, which is similar to that in human studies [41, 42]. Approximately 74.8%, 63.2% and 42.1% of these crossovers were arranged into the interval of 200, 100, and 30 kb, respectively, indicating a high level of resolution (**Fig. S4**). The distribution of distances between adjacent crossovers was not uniform, with a peak at approximately 50Mb (**Fig. S5**). Compared to non-crossover regions with a density of 66.3 PRDM9 binding motifs (CCnCCnTnnCCnC) per Mb, we found a higher density of 69.2 binding motifs per Mb around crossovers, indicating a potential role of PRDM9 in regulating meiotic recombination hotspots.

Recombination hotspots are crucial for ensuring the proper segregation of meiotic chromosomes and generating genetic diversity in offspring [43-45]. We calculated the recombination rate with a 3Mb sliding window and identified two distinct recombination hotspots, both located on chromosome 8 (**Fig. 3b**). These hotspot regions contained 31 genes. By performing functional enrichment analyses in using DAVID [46], we found that the most significant functional terms was biomineral tissue development (P=5.6E-4), which included three tooth-related genes (IBSP, SPP1, MEPE) (**Table S3**). MEPE, in particular, is thought to be strongly positively selected in herbivorous mammalian lineages and plays a crucial role in promoting the formation and mineralization of dentin, thus contributing to the strength of tooth structure [47]. Notably, buffaloes are known to efficiently utilize coarse feed, such as straw, sunflower cakes and sprouts, and convert them into valuable animal products [10, 48, 49]. Recombination hotspots may provide genetic diversity to these tooth-related genes, but further experimental validation is required to confirm their functional roles.

Factors affecting the recombination rate

To determine which factor(s) have the greatest impact on recombination rates, several such as PRDM9 binding, TEs and GC content have been investigated. We performed a correlation analysis between these genomic features and the recombination rates. The effects of density and length were analyzed separately for genes and TEs. We found that gene density and length had almost equal correlations with recombination rates, but for TEs, the density was significantly more correlated than the length (**Fig. 4a, c, S6** and **S7**). Ultimately, among the factors analyzed, TE density was identified as the most influential factor on recombination rates in buffalo (**Fig. 4a-d**).

Previous studies have reported that TEs are also the main source of SV [50]. Therefore, it is speculated that TE may affect the formation of SV by increasing the frequency of recombination. We further investigated the relationship between TE subfamilies and recombination rates as well as SVs, and found that SINE/tRNA had a strong correlation with both recombination rates and SVs (**Fig. 4e-f**). SINE/tRNA was also found to be an important source of SV in pigs [51]. However, further evidence is needed to validate the functional role of SINE/tRNA in both recombination and SVs of swamp buffalo.

Discussion

We present here the chromosome-scale genome of male buffalo, which exhibits better contiguity than published buffalo genomes [9, 14]. In addition, We conducted whole-genome sequencing of 78 sperms from the same male buffalo and constructed the first recombination map for buffalo. The high-quality genome, particularly the Y chromosome, and the recombination map provide valuable resources for evolutionary, breeding and comparative genomic researches of swamp buffalo. Our study could have significant implications for the agricultural sector, particularly in regions where swamp buffalo are an important livestock resource. Our research may also have broader implications for the study of genome evolution and recombination, which can provide insights into the genetic mechanisms that drive species diversification and adaptation. The study has the potential to impact the daily lives of farmers through its contributions to the breeding of water buffaloes for meat and milk production. By identifying genetic variation related to desirable traits and using this information in breeding programs, farmers can improve the productivity and profitability of their herds.

The assembly of the Y chromosome presents a significant challenge due to abundant and lengthy repeats, reduced sequencing depth and high homology with some regions of the X chromosome [16]. In this study, we overcame these challenges by performing deep long- and ultra-long read sequencing (~105X) for the male buffalo. We used the SRY software [17] to sort the long reads of the Y chromosome, and these reads were separately assembled to overcome the last factor. We identified the contig of the PAR through the interaction relationship of the HiC heatmap, and phased them by combining the second- and third-generation reads and HiC data. Finally, we obtained the buffalo Y genome with a total length of 17.2 Mb, which is well mapped by 92% of the annotated genes in the bull Y genome (Btau_5.0.1). The assembly process for the buffalo Y chromosome can also be applied to other animals and plants containing sex-specific chromosomes or fragments.

Meiotic recombination is well-studied in model species [5, 41, 42, 52], but less so in livestock. We sequenced 78 buffalo sperms and identified 1,956 recombination events with an average of 25.1 crossovers per sperm cell, which is similar to that of humans [52]. The fine-scale recombination map revealed two recombination hotspots on chromosome 8 with significantly higher recombination rates than elsewhere in the swamp buffalo genome. Intriguingly, genes near these hotspots were most significantly related to tooth quality. Given that buffalo's primary food source is low-quality food such as plant straw, recombination hotspots may generate genetic diversities in tooth-associated genes to better adapt to the consumption of crude fiber diets.

Several factors, such as PRDM9 binding, TEs, and GC content, can influence recombination rates. We found that TE density had the strongest correlation with the recombination rate of swamp-type buffalo. Furthermore, SINE/tRNA, a TE subfamily, was found to have a significant effect on both recombination rate and SVs. We speculate that this SINE/tRNA subfamily may contribute to intra-species or inter-species genetic variation by promoting recombination. Several studies have shown that the ZnF domain of PRDM9 recognizes specific DNA motifs and is responsible for the formation of recombination hotspots [43, 53-55]. However, the rapid evolution of PRDM9 results in changes in the DNA sequence it binds to [56]. The 13bp motif (CCnCCnTnnCCnC) in human we used may not be optimal for the swamp buffalo PRDM9 binding requirements, which could lead to a weaker effect of the PRDM9 binding sequences on recombination frequency than TEs. Further functional assays are needed to determine the binding motif of swamp buffalo PRDM9. Nevertheless, compared with other factors except for PRDM9 binding, TE density has a relatively high correlation with the recombination rate.

In the future, the genome and recombination map of male river buffalo could be constructed, providing insights into the divergent domestication features between the two sub-species of water buffalo and facilitating modern breeding for meat and milk production, and identify genetic variation related to traits of interest. Additionally, further functional assays need to be performed to characterize the binding motif of swamp buffalo PRDM9, which may lead to a better understanding of the factors affecting recombination rates. We plan to continue investigating the genetic basis of important traits in swamp buffalo, and to explore ways to use this information to improve breeding programs and animal welfare. We also hope to develop new technologies and methodologies for studying the genetics of non-model organisms.

Method

Sample collection and sequencing

We sampled blood DNA from a local male buffalo in Guangxi Zhuang Autonomous Region. To construct a high-quality genome of the male swamp buffalo, several platforms including Illumina, nanopore, Bionano and HiC were used to generate a bulk of datasets. Bionano Saphyr technology was applied and DLE1 restriction enzyme was used for digestion. Illumina Hi-C technology was used in this study. For the construction of Hi-C libraries, the buffalo DNA was digested with the restriction enzyme MboI and then was sequenced on Illumina Novoseq 6000 platform ((RRID:SCR_016387)) with PE100 reads. We generated about 466.1Gb (174X) Illumina short reads, 271.9Gb (102X) nanopore long reads, 561.4Gb (210X) bionano molecules and 291.8Gb (109X) HiC data (Table S4). The HiC data was used to scaffold the primary genome assembly, and Bionano data was further used to manually check the order and orientation. The sperm was collected at reproductive medical and genetic Center of the people's hospital of guangxi zhuang autonomous region and sequenced according to the previous study [52]. We also sampled 14 tissues including dorsal muscle, lung, liver, spleen, tongue, kidney, heart, hind leg, fore leg, adipose tissue, conarium, hypothalamus, cerebellum, medulla oblongata and 7 rough Brodmann areas of the cerebral cortex (BA7/20, BA21/22/41/42, BA23/31/35, BA24/32, BA43, BA11/25 and BA44/45/46) of the buffalo for RNA sequencing on the Illumina Hiseq 2000 platform (RRID:SCR_020132) [57]. The details of sperm and transcriptome data are provided in Table S5 and S6, separately. The cortical divisions are in reference to human [58].

Separation of Long reads belonging to Y chromosome

We selected short-read datasets from 59 male swamp buffaloes and 62 female swamp buffaloes from our previous buffalo population study [9]. The datasets and long reads of the reference male buffalo were delivered to the SRY software (v1.5) [17] to identify Y-specific kmers and separated long reads belonging to Y chromosome.

Genome assembly

The long reads of the Y chromosome and other chromosomes of the male swamp buffalo were assembled with nextdenovo (v2.4.0) [59], respectively. All of the assembled contigs were polished by nextpolish (v1.3.1) [60] with settings (-max_depth 270 for short-read mapping options, and -min_read_len 1k and -max_depth 200 for long-read mapping options) using short reads. We used juicer (v1.5.7) [61] to align HiC data onto the male buffalo genome, and identified a PAR region candidate contig, ctg000160, which strongly interacts with both X and Y sequences. Then, the extractHAIRS program in HapCUT2 (--indels 1) [62] was used to phase the ctg000160 contig based on the alignments of genomic short reads, nanopore reads and HiC reads. The two haplotypes were mapped to the X chromosome sequences of the female swamp buffalo using the mummer software [63], and the more similar one was considered to belong to the PAR of the X chromosome. Finally, we used 3d-dna (v180922) [64] with

HiC data to anchor the contigs and manually adjust their orders in Juicebox as well as check with Bionano data for generating a chromosome-level genome. The completeness and accuracy of the final assemblies were estimated using both BUSCO (RRID:SCR_015008) v5.4.3 [65], Merqury (RRID:SCR_022964) v1.3 [21] and short read alignment.

Repeat annotation

We combined *de novo* and homology-based approaches to identify repetitive elements in the male buffalo genome. For the *de novo* approach, we used RepeatModeler (RRID:SCR_015027) v1.0.11 [66] to construct a de novo repeat library with default parameters. Then, RepeatMasker (RRID:SCR_012954 (v4.0.9) [66] was run on the male buffalo genome using the de novo library. RepeatMasker was also run against RepBase (RRID:SCR_021169) (v20181026) [66] for homologous repeat identification. The results of repeat annotation from the two approaches were integrated. TRF (v4.09) [67] with parameters "1 1 2 80 5 200 2000" was used to detect tandem repeats and search 6-mer vertebrate telomeric repeats (TTAGGG or alternative types including CCCTAA, TAGGGT, ACCCTA, AGGGTT, AACCCT, GGGTTA, TAACCC, GGTTAG, CTAACC, GTTAGG and CCTAAC). To identify centromeric regions of the male swamp buffalo, centromeric repeats of river buffalo and cattle [68] were aligned to the genome of male swamp buffalo using BLASR (RRID:SCR_000764) (v5.3.3) [69] with at least 70% identities.

Gene annotation

Three methods including *de novo*, homolog-based and transcriptome-based approaches were used to predict protein-coding genes of male buffalo. To perform de novo predictions, we used Augustus (RRID:SCR_008417) [70], Genscan (RRID:SCR_012902) [71], GlimmerHMM (RRID:SCR_002654) [72] and SNAP (RRID:SCR_007936) [73] in the repeat-masked genome sequences. For the homology-based predictions, we downloaded protein sequences of human, mouse, cow, sheep and horse from the Ensembl database and cow Y chromosome from NCBI, and aligned them to the male buffalo genome using tblastn (e-value < 10-5). genBlastA (v1.0.138) [74] was then used to cluster the adjacent HSPs (high-scoring pairs) from the same protein alignments, and exonerate (v2.4.0) [75] was used to identify accurate gene structures. After QC and filtering, reads from all RNA libraries and the testis transcriptome (NCBI accession: PRJEB25226) were mapped to the male buffalo genome using HISAT (v2.1.0) [76], and StringTie (RRID:SCR_016323) (v2.0.6) [77] was subsequently used to predict gene models. Finally, we combined all predicted genes from the three methods with EVidenceModeler (RRID:SCR_014659) (r2012-06-25) [78] and filtered out genes with less than 50% transcriptome coverage to generate high-confidence gene sets.

To obtain gene functional annotation, the SwissProt protein database [79] was searched with blastp (RRID:SCR_001010) (ncbi-blast-2.9.0+) (e-value<10−5). The best hits were used to assign homology-based gene functions. We used DAVID (RRID:SCR_001881) (v6.8) [80] to perform functional analysis for candidate genes

under a current background (*Homo sapiens*) with the Fisher's exact test.

Detection of SVs

We utilized the nucmer program in Mummer package (RRID:SCR_018171) (v4.0.0beta2) [63] to perform genome alignments between male swamp buffalo (or river buffalo) and cattle. The resulting delta file was delivered to Assemblytics (v1.2.1) [81] for calling SVs. We set the parameters of Assemblytics with "10000 50 1000000" corresponding to unique alignment length, minimum and maximum size of SVs, respectively. We applied the chisq.test function in the R package for the gene-expression comparison of SV-inserted or unique SV-inserted genes with all genes of the male swamp buffalo. The results are listed in Table S7 and S8. Notably, SVs in this study refer to fixed genomic differences between swamp and river buffalo and cattle, and not to variants within a population.

Calculating dN/dS

To compute the dN/dS value of genes on the Y chromosome, we used blastp (ncbi-blast-2.9.0+) with e-value<1-E05 to generated protein alignments for genes in the PARs of the X and Y chromosomes as well as self-to-self alignments for genes in SDR. Optimal alignments other than to themselves were considered as homologous gene pairs. The yn00 in the PAML package (RRID:SCR_014932) (v4.9) [82] was further used to calculate dN/dS values of paralogs.

SNP calling

Sequencing short reads for each sperm were mapped onto the male buffalo genome using BWA (v0.7.17-r1188)[83]. Bam files for the same sample were merged using samtools (RRID:SCR_002105) (v1.9) [84]. Duplicate reads were removed using the rmdup command in samtools with default parameters. We used samtools mpileup with settings (-C 50 --min-MQ 30 --min-BQ 30) to call SNPs of all 78 sperms together. Using the samtools mpileup and bcftools [84] filter command (-e "%QUAL<30 || DP<30 || DP>200" -g 5 -G 5), we called SNPs for the male buffalo reference. The genotype of single sperm should be consistent with that of the paternal genome, so we selected heterozygous SNPs of the sperms consistent with the reference heterozygous SNP site for the identification of recombination events. To detect crossover events in PAR, we aligned both X- and Y-single sperm to the PAR of the Y chromosome to identify bi-allelic SNPs.

Identifying recombination events in sperms

To detect recombination events in sperm, the Hapi package [40] in R was used to process the heterozygous SNP results of sperm. We followed the operations recommended by the Hapi software step by step. Firstly, we used the 'hapiErrorFilter' function with default parameters to remove the potential genotyping errors of sperms. Secondly, heterozygous SNPs that were genotype in at least 10 sperm (n=10) were selected for constructing the high□quality framework by the 'hapiFrameSelection' function, separately. Imputation of missing data was performed by the 'hapiImupte'

function with settings (nSPT=3, allowNA=0). Thirdly, we inferred and proofread draft haplotypes by ‘hapiPhase’ and ‘hapiCVCluster’ functions. Multiple crossovers (cv□links ≥ 2) within 1 Mb were filtered. We further adopted a Maximum Parsimony of Recombination (MPR) strategy to eliminate incorrect crossovers by the ‘hapiBlockMPR’ function. Fourthly, chromosome-level haplotype assembly was achieved by the ‘hapiAssemble’ function and the haplotypes located at the end of the chromosome were polished using the ‘hapiAssembleEnd’ function with default parameters. Finally, we identified crossovers in sperm by the ‘hapiIdentifyCV’ function based on haplotypes for each sperm. Notably, some recombination events may not be accurately identified despite strict conditions for the process of sperm genotyping and recombination event identification.

Tables

	Species	Contig		Scaffold		Busco
		Total length (Mb)	N50 (Mb)	Total length (Mb)	N50 (Mb)	
**This study**	**Swamp buffalo (male)**	2,675	72.2	2,675	120.0	95.8%
**Low et al. (2020)**	**River buffalo (female)**	2,654	18.8	2,654	117.2	94.0%
**Luo et al. (2020)**	**Swamp buffalo (female)**	2,609	8.8	2,631	117.3	95.2%
**Luo et al. (2020)**	**River buffalo (female)**	2,626	3.1	2,646	116.1	95.7%

Table 1. Comparison of the genome assemblies of three buffaloes. The orthologous gene dataset used for busco evaluation is mammalia_odb10 (v2021-02-19).

Figure legend

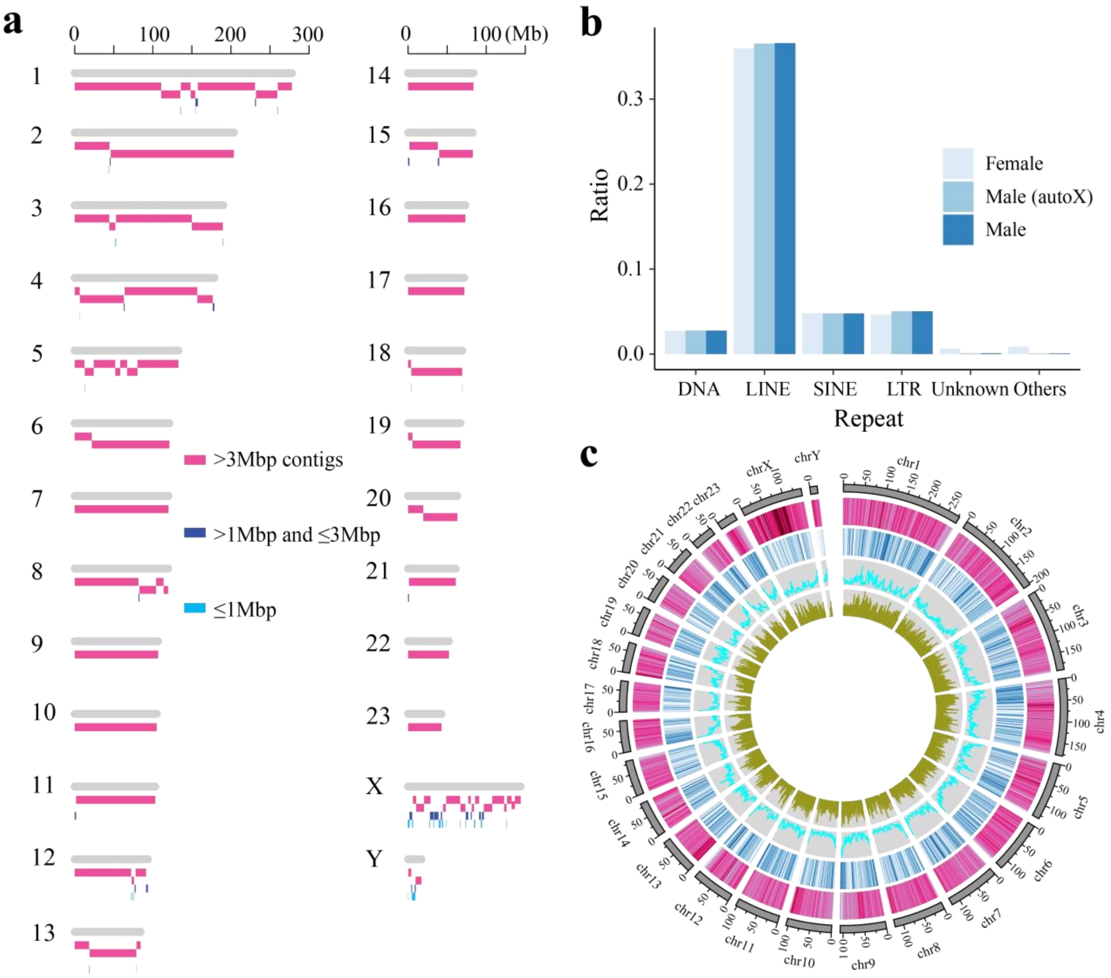

Fig. 1: Chromosome-scale assembly of the male swamp buffalo. a The distribution of contigs on chromosomes. The assembled results were divided into three types of contigs larger than 3Mb (pink), between 1Mb and 3Mb (dark blue) and smaller than 1Mb (light blue) according to their lengths. **b** Comparison of repetitive content between male and female buffaloes. Male buffalo genome containing only X and autosomes was labeled as “Male (autox)”. **c** Circos plot of male buffalo genome. The tracks from outer to inner circles (a-d) indicated the following: chromosomes, TE coverage, gene coverage, GC contents, and gene expression, respectively.

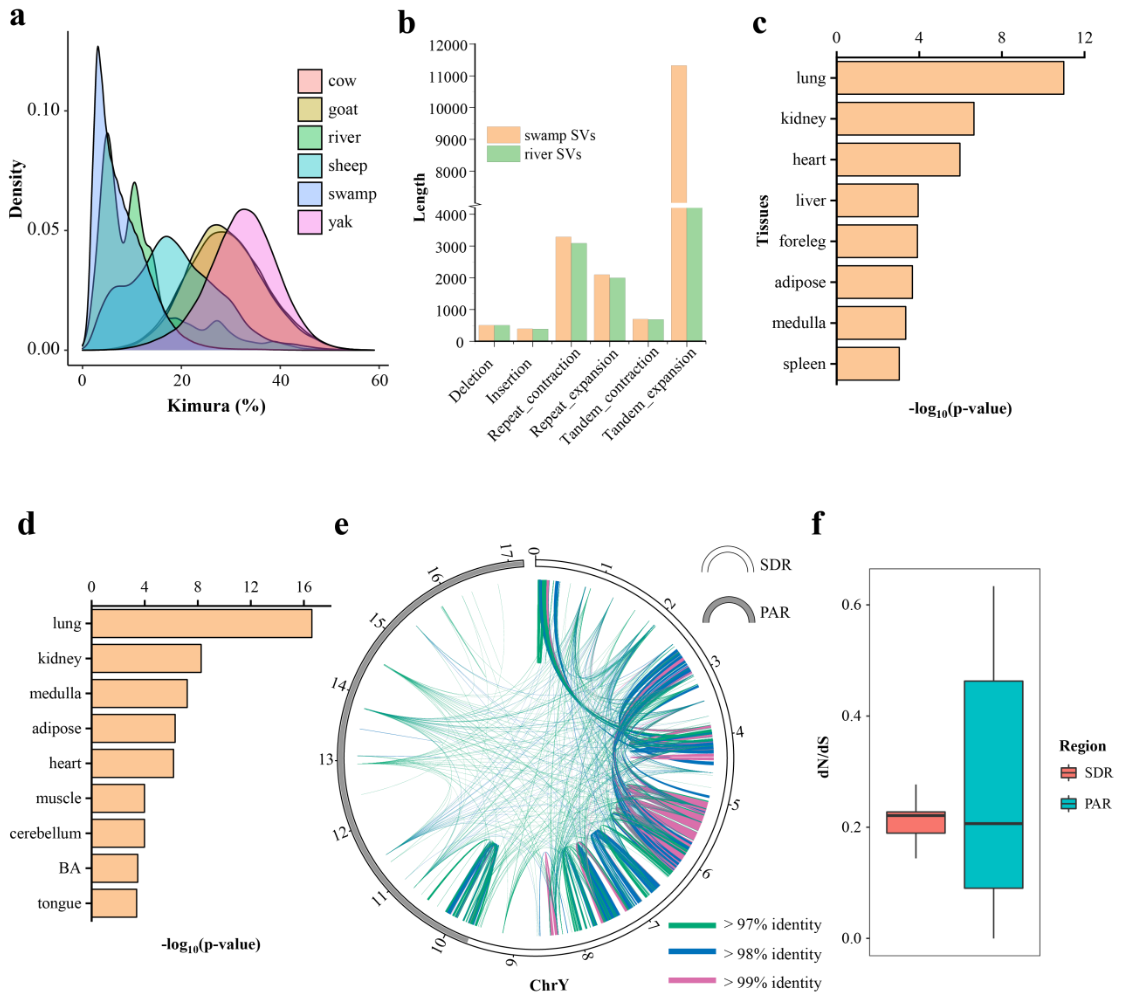

Fig. 2: Genomic features of the male buffalo genome. a Kimura divergence of TE subfamily LINE/RTE-BovB. The kimura values were calculated by RepeatMasker. **b** Distribution of the SV lengths of male swamp buffalo and river buffalo. **c-d** Tissue distributions of SV-inserted (**c**) and unique SV-inserted (**d**) genes with the highest expression levels. Only tissues that are significantly enriched (p-value < 0.05) for genes within SVs compared to all swamp buffalo genes are shown. **e** Intrachromosomal similarities in Y chromosome of the male buffalo. As shown, line colors represent the minimum identities (only hits >500bp are plotted). **f** Comparison of the dN/dS values in two regions of Y chromosome.

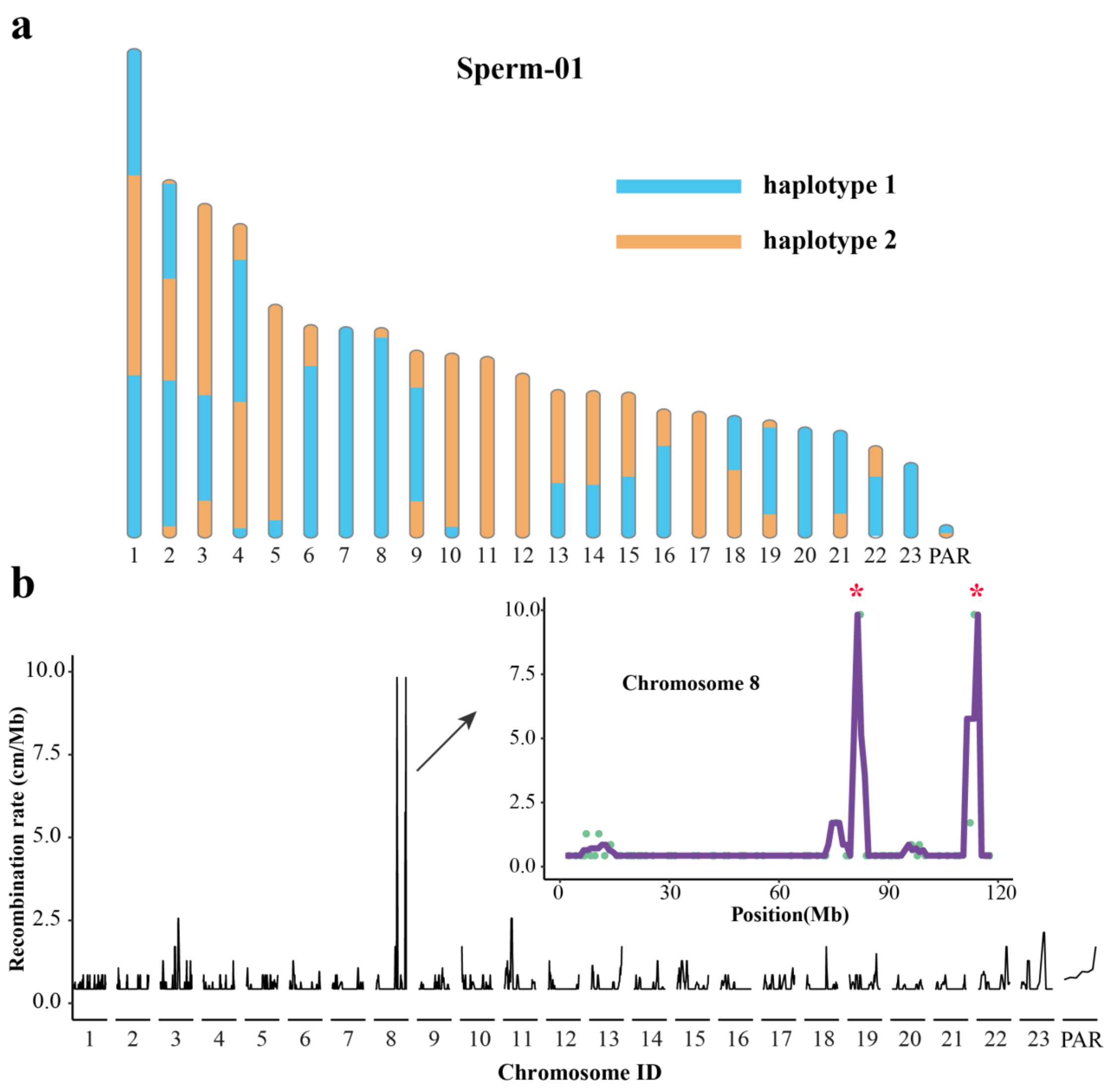

Fig. 3: Detecting position of recombination and hotspots. a An example of identified recombination maps for the single sperm with ID "Sperm-01". **b** Distribution of recombination rates across all chromosomes in male swamp buffalo. The distribution of recombination rates on chromosome 8 is amplified. Green circles represent the recombination rate for each bin (3Mb length), and asterisks represent the locations of recombination hotspots.

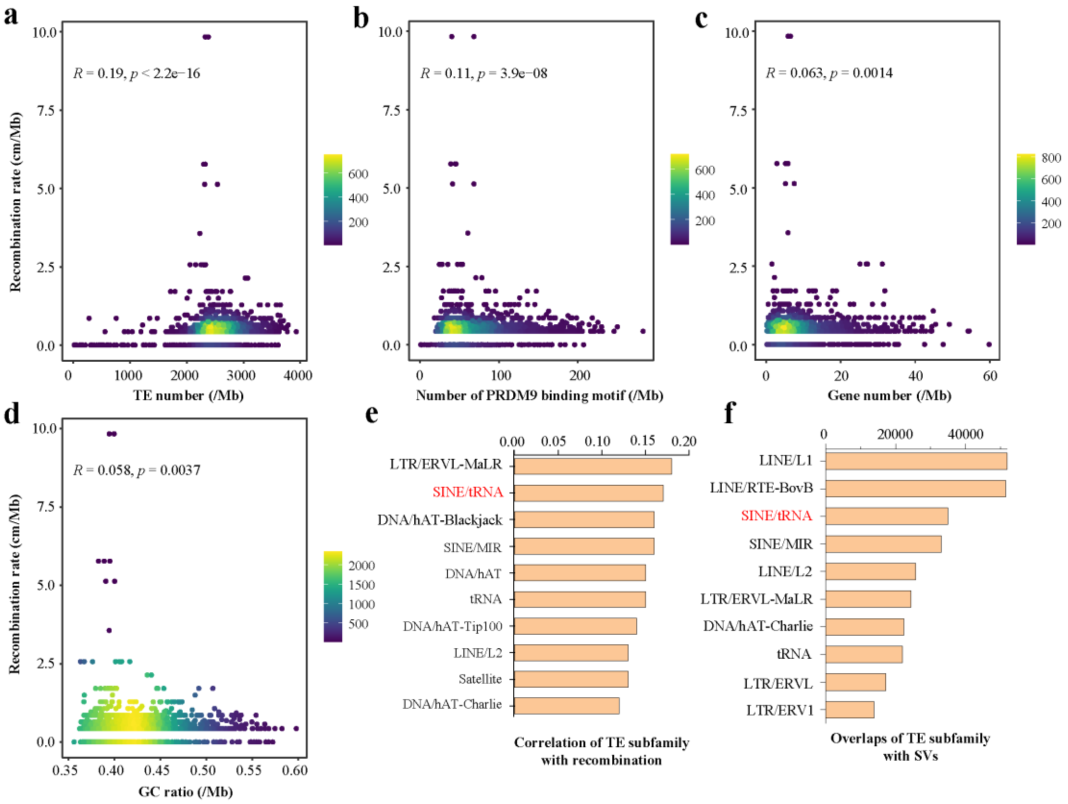

Fig. 4: Influencing factors of recombination rate. a-d Spearman's rank correlation analysis of recombination rate with various genomic features, including TE density (**a**), PRDM9 (**b**), gene density (**c**) and GC content (**d**). Each point represents a bin (3Mb length), and the color represents the number of bins as shown. **e** The top ten TE subfamilies most associated with recombination rates. **f** The top ten TE subfamilies contained in SV. The top-ranked SINE/tRNA in both **e** and **f** is highlighted in red.

Data Availability

The genomic sequencing reads were deposited in Genome Sequence Archive in National Genomics Data Center, with the accession number CRA007045. The genome assembly and gene annotation of the male swamp buffalo were deposited in figshare [85]. All supporting data are available in the GigaScience GigaDB database [86].

Additional Files

Supplementary Figure S1. The interaction between the candidate contig ctg000160 in the PAR region and the contigs of the X and Y chromosomes.
Supplementary Figure S2. The heatmap (resolution: 500kb) of the male buffalo genome. The increase in interaction signal is represented from yellow to red color.
Supplementary Figure S3. The distribution of closed gap lengths (bin size 5kb).
Supplementary Figure S4. Distribution of the internal sizes.
Supplementary Figure S5. Distribution of distances between adjacent recombinations.
Supplementary Figure S6. Correlation between SV length and recombination rate.
Supplementary Figure S7. Correlation between gene length and recombination rate.
Supplementary Table S1. Statistics of predicted protein-coding genes in the male buffalo genome.
Supplementary Table S2. Analysis of transposable elements (TEs) in the male buffalo genome.
Supplementary Table S3. Functional enrichment of genes around recombination hotspots.

Abbreviations

BUSCO: Benchmarking Universal Single-Copy Orthologs; Mb: megabase pairs; KEGG: Kyoto Encyclopedia of Genes and Genomes; GO: gene ontology; NCBI: The National Center for Biotechnology Information; QV: quality value.

Ethics Statement

Samples were provided from collaborators for research that was undertaken at Foshan University, permit FOSU2023001 from the School of Life Science and Engineering.

Competing Interests

The authors declare no competing interests.

Authors' Contributions

Q.L., J.R. and Z.L. designed and leaded the project. X.W. analyzed genome and sperm data and drafted the paper. X.W., T.F., X.L. and R.S. analyzed the transcriptome data. L.X. and K.H. sampled and processed the experimental materials. Q.L. revised the manuscript. K.C., H.L., J.H., C.M., D.W. and D.S. provided suggestions and helped with the checking.

ACKNOWLEDGEMENTS

This research is supported by Guangxi Science and Technology Major Project (2021AA20037, AA22068099) and the National Natural Science Foundation of China (U20A2051, 31760648 and 31860638),.

References

1. Petes and Thomas D. Meiotic recombination hot spots and cold spots. *Nat Rev Genet*. 2001;2(5):360-369.
2. Paigen K and Petkov P. Mammalian recombination hot spots: properties, control and evolution. *Nat Rev Genet*. 2010;11(3):221-233.
3. Capilla L, Caldés MG and Ruiz-Herrera A. Mammalian meiotic recombination: a toolbox for genome evolution. *Cytogenet Genome Res*. 2016;150(1):1-16.
4. Fuentes RR, de Ridder D, van Dijk AD and Peters SA. Domestication shapes recombination patterns in tomato. *Mol Biol Evol*. 2022;39(1):msab287.
5. Hinch AG, Zhang G, Becker PW, Moralli D, Hinch R, Davies B, et al. Factors influencing meiotic recombination revealed by whole-genome sequencing of single sperm. *Science*. 2019;363(6433):eaau8861.
6. Cavassim MIA, Andersen SU, Bataillon T and Schierup M. Recombination Facilitates Adaptive Evolution in Rhizobial Soil Bacteria. Molecular Biology and Evolution. 2021, 38(12): 5480–5490. https://doi.org/10.1093/molbev/msab247.
7. Scherf BD. *World watch list for domestic animal diversity*. Food and Agriculture Organization (FAO); 2000.
8. Cockrill WR, Fao R and AGA. The husbandry and health of the domestic buffalo. *Trop Anim Health Pro*. 1975;7(1).
9. Luo X, Zhou Y, Zhang B, Zhang Y, Wang X, Feng T, et al. Understanding divergent domestication traits from the whole-genome sequencing of swamp-and river-buffalo populations. *Natl Sci Rev*. 2020;7(3):686-701.
10. Ivanova S and Markov N. Investigation of the feed resource for buffalo. *Acta Scientiarum Animal Sciences*. 2021;43.
11. Ranjhan S. Nutrition of river buffaloes in Southern Asia. *Buffalo Production*. 1992:111-134.
12. O'Brien B and Hennessy D. Scientific appraisal of the Irish grass-based milk production system as a sustainable source of premium quality milk and dairy products. *Irish J Agr Food Res*. 2017;56(1):120-129.
13. Pisano MB, Scano P, Murgia A, Cosentino S and Caboni P. Metabolomics and microbiological profile of Italian mozzarella cheese produced with buffalo and cow milk. *Food Chem*. 2016;192:618-624.
14. Low WY, Tearle R, Bickhart DM, Rosen BD, Kingan SB, Swale T, et al. Chromosome-level assembly of the water buffalo genome surpasses human and goat genomes in sequence contiguity. *Nature Commun*. 2019;10(1):1-11.
15. Li H, Huang K, Wang P, Feng T, Shi D, Cui K, et al. Comparison of long non-coding RNA expression profiles of cattle and buffalo differing in muscle characteristics. *Front Genet*. 2020;11:98.

16. Tomaszkiewicz M, Medvedev P and Makova KD. Y and W Chromosome Assemblies: Approaches and Discoveries. *Trends Genet*. 2017;33(4):266-282.
17. Wang X-B, Liu Q-Y, Li A-L and Ruan J. SRY: an effective method for sorting long reads of sex-limited chromosome. bioRxiv. 2020.
18. Shaari NAL, Jaoi-Edward M, Loo SS, Salisi MS, Yusoff R, Ab Ghani NI, et al. Karyotypic and mtDNA based characterization of Malaysian water buffalo. BMC Genet. 2019;20 1:1-6.
19. Iannuzzi L. Standard karyotype of the river buffalo (Bubalus bubalis L., 2n= 50). Report of the committee for the standardization of banded karyotypes of the river buffalo. *Cytogenet Cell Genet*. 1994;67 2:102-13.
20. Rehman SU, Hassan F-u, Luo X, Li Z and Liu Q. Whole-genome sequencing and characterization of buffalo genetic resources: recent advances and future challenges. *Animals*. 2021;11(3):904.
21. Rhie A, Walenz BP, Koren S and Phillippy AM. Merqury: reference-free quality, completeness, and phasing assessment for genome assemblies. *Genome Biol*. 2020;21(1):1-27.
22. Chuong EB, Elde NC and Feschotte C. Regulatory activities of transposable elements: from conflicts to benefits. *Nat Rev Genet*. 2017;18(2):71-86.
23. Dougan G and Sherratt D. The transposon Tn 1 as a probe for studying ColE1 structure and function. *Mol Gen Genet*. 1977;151(2):151-160.
24. Kleckner N. Transposable elements in prokaryotes. Annu Rev Genet. 1981;15 1:341-404.
25. Hutchison III CA, Peterson SN, Gill SR, Cline RT, White O, Fraser CM, et al. Global transposon mutagenesis and a minimal Mycoplasma genome. *Science*. 1999;286(5447):2165-2169.
26. Studer A, Zhao Q, Ross-Ibarra J and Doebley J. Identification of a functional transposon insertion in the maize domestication gene tb1. *Nat Genet*. 2011;43(11):1160-1163.
27. Emera D and Wagner GP. Transformation of a transposon into a derived prolactin promoter with function during human pregnancy. *Proc Natl Acad Sci*. 2012;109(28):11246-11251.
28. Kordišs D and Gubenšsek F. Horizontal transfer of non-LTR retrotransposons in vertebrates. *Genetica*. 1999;107(1):121-128.
29. Kordis D and Gubensek F. Unusual horizontal transfer of a long interspersed nuclear element between distant vertebrate classes. *Proc Natl Acad Sci*. 1998;95(18):10704-10709.
30. Roach JC, Glusman G, Smit AF, Huff CD, Hubley R, Shannon PT, et al. Analysis of genetic inheritance in a family quartet by whole-genome sequencing. *Science*. 2010;328(5978):636-639.
31. Kronenberg ZN, Fiddes IT, Gordon D, Murali S, Cantsilieris S, Meyerson OS, et al. High-resolution comparative analysis of great ape genomes. *Science*. 2018;360(6393):eaar6343.
32. Hurles ME, Dermitzakis ET and Tyler-Smith C. The functional impact of structural variation in humans. *Trends Genet*. 2008;24(5):238-245.
33. Zhang L, Reifová R, Halenková Z and Gompert Z. How important are structural variants for speciation? *Genes*. 2021;12(7):1084.
34. Anderson JE, Kantar MB, Kono TY, Fu F, Stec AO, Song Q, et al. A roadmap for functional structural variants in the soybean genome. *G3: Genes, Genomes, Genetics*. 2014;4(7):1307-1318.

35. Yalcin B, Wong K, Agam A, Goodson M, Keane TM, Gan X, et al. Sequence-based characterization of structural variation in the mouse genome. *Nature*. 2011;477(7364):326-329.
36. Sudmant PH, Rausch T, Gardner EJ, Handsaker RE, Abyzov A, Huddleston J, et al. An integrated map of structural variation in 2,504 human genomes. *Nature*. 2015;526(7571):75-81.
37. Fuentes RR, Chebotarov D, Duitama J, Smith S, De la Hoz JF, Mohiyuddin M, et al. Structural variants in 3000 rice genomes. *Genome Res*. 2019;29(5):870-880.
38. Rozen S, Skaletsky H, Marszalek JD, Minx PJ, Cordum HS, Waterston RH, et al. Abundant gene conversion between arms of palindromes in human and ape Y chromosomes. *Nature*. 2003;423(6942):873-876.
39. Hughes JF, Skaletsky H, Pyntikova T, Koutseva N, Raudsepp T, Brown LG, et al. Sequence analysis in Bos taurus reveals pervasiveness of X–Y arms races in mammalian lineages. *Genome Res*. 2020;30(12):1716-1726.
40. Li R, Qu H, Chen J, Wang S, Chater JM, Zhang L, et al. Inference of chromosome-length haplotypes using genomic data of three or a few more single gametes. *Mol Biol Evol*. 2020;37(12):3684-3698.
41. Wang J, Fan HC, Behr B and Quake SR. Genome-wide single-cell analysis of recombination activity and de novo mutation rates in human sperm. *Cell*. 2012;150(2):402-412.
42. Bell AD, Mello CJ, Nemesh J, Brumbaugh SA and Mccarroll SA. Insights into variation in meiosis from 31,228 human sperm genomes. *Nature*. 2020;583(7815):1-6.
43. Parvanov ED, Petkov PM and Paigen K. Prdm9 controls activation of mammalian recombination hotspots. *Science*. 2010;327(5967):835-835.
44. Singhal S, Leffler EM, Sannareddy K, Turner I, Venn O, Hooper DM, et al. Stable recombination hotspots in birds. *Science*. 2015;350(6263):928-932.
45. Gerton JL, DeRisi J, Shroff R, Lichten M, Brown PO and Petes TD. Global mapping of meiotic recombination hotspots and coldspots in the yeast Saccharomyces cerevisiae. *Proc Natl Acad Sci*. 2000;97(21):11383-11390.
46. Huang DW, Sherman BT and Lempicki RA. Bioinformatics enrichment tools: paths toward the comprehensive functional analysis of large gene lists. *Nucleic Acids Res*. 2009;37(1):1-13.
47. Mu Y, Tian R, Xiao L, Sun D, Zhang Z, Xu S, et al. Molecular Evolution of Tooth-Related Genes Provides New Insights into Dietary Adaptations of Mammals. *J Mol Evol*. 2021;89(7):458-471.
48. Kandeepan G, Biswas S and Rajkumar R. Buffalo as a potential food animal. *Int J Livest Prod*. 2009;1(1):1-5.
49. Kandeepan G, Mendiratta S, Shukla V and Vishnuraj M. Processing characteristics of buffalo meat-a review. *J Meat Sci Technol*. 2013;1(1):1-11.
50. Muotri AR, Marchetto MC, Coufal NG and Gage FH. The necessary junk: new functions for transposable elements. *Hum Mol Genet*. 2007;16(R2):R159-R167.
51. Ai H, Fang X, Yang B, Huang Z, Chen H, Mao L, et al. Adaptation and possible ancient interspecies introgression in pigs identified by whole-genome sequencing. *Nat Genet*. 2015;47(3):217-25.

52. Lu S, Zong C, Fan W, Yang M, Li J, Chapman AR, et al. Probing Meiotic Recombination and Aneuploidy of Single Sperm Cells by Whole-Genome Sequencing. *Science*. 2012;338(6114):1627-1630.
53. Myers S, Bowden R, Tumian A, Bontrop RE, Freeman C, MacFie TS, et al. Drive against hotspot motifs in primates implicates the PRDM9 gene in meiotic recombination. *Science*. 2010;327(5967):876-879.
54. Baudat F, Buard J, Grey C, Fledel-Alon A, Ober C, Przeworski M, et al. PRDM9 is a major determinant of meiotic recombination hotspots in humans and mice. *Science*. 2010;327(5967):836-840.
55. Brick K, Smagulova F, Khil P, Camerini-Otero RD and Petukhova GV. Genetic recombination is directed away from functional genomic elements in mice. *Nature*. 2012;485(7400):642-645.
56. Ahlawat S, De S, Sharma P, Sharma R, Arora R, Kataria R, et al. Evolutionary dynamics of meiotic recombination hotspots regulator PRDM9 in bovids. *Mol Genet Genomics*. 2017;292(1):117-131.
57. Xiaobo W, Hassan F-u, Liu S, Yang S, Ahmed M, Ahmed I, et al. De Novo Transcriptome Dataset Generation of the Swamp Buffalo Brain and Non-Brain Tissues. *BioMed Res Int.* 2022.
58. Carter R. *The human brain book: An illustrated guide to its structure, function, and disorders*. Penguin; 2019.
59. Hu J, Wang Z, Sun Z, Hu B, Ayoola AO, Liang F, et al. An efficient error correction and accurate assembly tool for noisy long reads. bioRxiv. 2023.
60. Hu J, Fan J, Sun Z and Liu S. NextPolish: a fast and efficient genome polishing tool for long-read assembly. *Bioinformatics*. 2020.
61. Durand NC, Shamim MS, Machol I, Rao SS, Huntley MH, Lander ES, et al. Juicer provides a one-click system for analyzing loop-resolution Hi-C experiments. Cell systems. 2016;3 1:95-8.
62. Edge P, Bafna V and Bansal V. HapCUT2: robust and accurate haplotype assembly for diverse sequencing technologies. *Genome Res*. 2017;27(5):801-812.
63. Delcher AL, Salzberg SL and Phillippy AMJCpib. Using MUMmer to identify similar regions in large sequence sets. *Curr Protoc Bioinformatics.* 2003;1:10.3.1-10.3.8.
64. Dudchenko O, Batra SS, Omer AD, Nyquist SK, Hoeger M, Durand NC, et al. De novo assembly of the Aedes aegypti genome using Hi-C yields chromosome-length scaffolds. *Science*. 2017;356(6333):92-95.
65. Simao FA, Waterhouse RM, Ioannidis P, Kriventseva EV and Zdobnov EM. BUSCO: assessing genome assembly and annotation completeness with single-copy orthologs. *Bioinformatics*. 2015;31(19):3210-3212.
66. Chen N. Using Repeat Masker to identify repetitive elements in genomic sequences. *Curr Protoc Bioinformatics*. 2004;5(1):4-10.
67. Benson G. Tandem repeats finder: a program to analyze DNA sequences. *Nucleic Acids Res*. 1999;27(2):573-580.
68. Melters DP, Bradnam KR, Young HA, Telis N, May MR, Ruby JG, et al. Comparative analysis of tandem repeats from hundreds of species reveals unique insights into centromere evolution. *Genome Biol*. 2013;14(1):1-20.

69. Chaisson MJ and Tesler G. Mapping single molecule sequencing reads using basic local alignment with successive refinement (BLASR): application and theory. *BMC Bioinformatics*. 2012;13(1):1-18.
70. Stanke M, Diekhans M, Baertsch R and Haussler D. Using native and syntenically mapped cDNA alignments to improve de novo gene finding. *Bioinformatics*. 2008;24(5):637-44.
71. Burge C and Karlin S. Prediction of complete gene structures in human genomic DNA. *J Mol Biol*. 1997;268(1):78-94.
72. Majoros WH, Pertea M and Salzberg SL. TigrScan and GlimmerHMM: two open source ab initio eukaryotic gene-finders. *Bioinformatics*. 2004;20(16):2878-2879.
73. Bromberg Y and Rost B. SNAP: predict effect of non-synonymous polymorphisms on function. *Nucleic Acids Res*. 2007;35(11):3823-3835.
74. She R, Chu JS, Wang K, Pei J and Chen N. GenBlastA: enabling BLAST to identify homologous gene sequences. *Genome Res*. 2009;19(1):143-149.
75. Slater GSC and Birney E. Automated generation of heuristics for biological sequence comparison. *BMC Bioinformatics*. 2005;6(1):1-11.
76. Kim D, Paggi JM, Park C, Bennett C and Salzberg SL. Graph-based genome alignment and genotyping with HISAT2 and HISAT-genotype. *Nat Biotechnol*. 2019;37(8):907-915.
77. Pertea M, Pertea GM, Antonescu CM, Chang T-C, Mendell JT and Salzberg SL. StringTie enables improved reconstruction of a transcriptome from RNA-seq reads. *Nat Biotechnol*. 2015;33(3):290-295.
78. Haas BJ, Salzberg SL, Zhu W, Pertea M, Allen JE, Orvis J, et al. Automated eukaryotic gene structure annotation using EVidenceModeler and the Program to Assemble Spliced Alignments. *Genome Biol*. 2008;9(1):R7.
79. Bairoch A, Apweiler R, Wu CH, Barker WC, Boeckmann B, Ferro S, et al. The universal protein resource (UniProt). *Nucleic Acids Res*. 2005;33(suppl_1):D154-D159.
80. Dennis G, Sherman BT, Hosack DA, Yang J, Gao W, Lane HC, et al. DAVID: database for annotation, visualization, and integrated discovery. *Genome Biol*. 2003;4(9):1-11.
81. Nattestad M and Schatz MC. Assemblytics: a web analytics tool for the detection of variants from an assembly. *Bioinformatics*. 2016;32(19):3021-3023.
82. Yang Z. PAML 4: phylogenetic analysis by maximum likelihood. *Mol Biol Evol*. 2007;24(8):1586-1591.
83. Li H and Durbin R. Fast and accurate short read alignment with Burrows–Wheeler transform. *Bioinformatics*. 2009;25(14):1754-1760.
84. Danecek P, Bonfield JK, Liddle J, Marshall J, Ohan V, Pollard MO, et al. Twelve years of SAMtools and BCFtools. Gigascience. 2021,10(2):giab008. doi: 10.1093/gigascience/giab008
85. Wang XB. The genome and annotation of the male swamp buffalo. Figshare. 2023. https://doi.org/10.6084/m9.figshare.19885720.v2.
86. Liu Q, Wang X, Li Z, Feng T, Luo X, Xue L et al. Supporting data for "Chromosome-level genome and recombination map of the male buffalo" GigaScience Database. 2023. http://dx.doi.org/10.5524/102407.

Click here to access/download
Supplementary Material
Supplement-r.docx

Personal Cover Click here to access/download;Personal Cover;comments-response.docx

Dear Editor,

Thank you for your feedback and suggestions on our article (GIGA-D-22-00319R1). We have carefully considered your comments and made the necessary revisions accordingly. Additionally, we have also incorporated the suggestions provided by the reviewer. We hope that the changes we have made have addressed all concerns and improved the overall quality of the article.

Thank you for your time and consideration.

Sincerely,
Qingyou Liu
qyliu-gene@fosu.edu.cn

Response to reviewer

Reviewer reports:
Reviewer #1: Thanks to the authors for their response. The manuscript looks good but I think there still are a couple of minor revisions needed. One is there are quite a few English errors so I have attached a tracked version of the manuscript where I have tried to fix/flag some of these in case useful. But think it could likely benefit from some further editing.
Response: Thank you for your feedback and suggestions. We have carefully reviewed and revised the manuscript according to your comments. We have also paid close attention to the English language errors and made necessary corrections. We appreciate your help in improving the quality of our manuscript.

Regarding the details of the sequencing technologies used. It is good to see the details the authors have added. But they still, for example, havnt specified things such as what HiC technology was used (Dovetail?), how was sequenced (Illumina? Read length?). Also for example was it Bionano Saphyr? I think this kind of information should be included in a genome assembly paper.
Response: Thank you for your comment. We apologize for any confusion caused by the lack of clarity in our manuscript. Illumina Hi-C technology was used in this study. For the construction of Hi-C libraries, the buffalo DNA was digested with the restriction enzyme MboI and then was sequenced on a Novoseq 6000 platform with PE100 reads. Bionano Saphyr technology was applied and DLE1 restriction enzyme was used for digestion. We have update our manuscript to include this information.

Also references are missing e.g. at line "Although several of female buffalo genomes have been finished " but they dont cite any. Or for example say "Besides, about 92% of the annotated Y genes in the bull genome could be explicitly..." and "which is well mapped by 92% of the annotated genes in the bull Y genome" but dont specify which bull or which annotations. Or where say "which exhibits better contiguity than

published buffalo genomes ". But none cited.

Response: Thank you for your valuable feedback on our manuscript. We apologize for the oversight in not including the necessary references in the mentioned sections. We have now revised the manuscript and included the appropriate citations.

Authors still dont say what this number represents "The homozygous single nucleotide polymorphism (SNP) ratio was approximately 3.39 x 10- 6 based on genomic short-read alignment". From the author response it sounds like this is homozygous variant calls per basepair. If so the authors should specify this (or whatever other unit it is if this is not correct).

Response: Yes, the number represents the homozygous variant calls per basepair.We have revised the sentence to read as follows: 'The homozygous single nucleotide polymorphism (SNP) ratio was approximately 3.39 x 10- 6 per basepair based on genomic short-read alignment.'

"We mapped both swamp and river buffalo to the cattle reference genome and used Assemblytics to detect SVs ". If are talking about differences between species I dont think can refer to them as SVs. The term SV is typically used to refer to variants within a species, not across them. (including this is how is defined on wikipedia https://en.wikipedia.org/wiki/Structural_variation). Here seem to be referring to what are likely fixed genomic differences.

Response: Thank you for the suggestion. While the term "SV" is typically used to describe variants within a species, some studies do use it to describe genomic differences between different species. For example, Li et.al have aligned the gayal genome to the cattle genome to identify SVs. Therefore, in our study, we have also used the term "SV" to describe the genomic differences between swamp and river buffalo and cattle. However, we appreciate the reviewer's suggestion and will emphasize the point in the Method in our article.

Li, Y., Wang, S., Zhang, Z., Luo, J., Lin, G. L., Deng, W. D., et al. (2023). Large-scale chromosomal changes lead to genome-level expression alterations, environmental adaptation, and speciation in the Gayal (*Bos frontalis*). *Molecular Biology and Evolution*, 40(1), msad006.

"In addition, we identified an average of 69.2 PRDM9 binding motif (CCnCCnTnnCCnC) per Mb around crossovers ." How does this compare to regions not around crossovers? Otherwise figure is a bit meaningless.

Response: The density of PRDM9 binding motifs in the non-crossover regions is 66.3 per Mb. We have revised the description in the article to “Compared to non-crossover regions with a density of 66.3 PRDM9 binding motifs per Mb, we found a higher density of 69.2 binding motifs per Mb around crossovers, indicating a potential role of PRDM9 in regulating meiotic recombination hotspots.”

"to perform functional analysis for candidate genes under a current

background (Homo sapiens) with the Fisher's exact test" should you not restrict the background to genes with an orthologue in buffalo.

Response:DAVID requires selecting a species as background for functional enrichment analysis. As human genes have been studied more extensively, we first identified the human orthologs of the buffalo genes and then used human as the background species in DAVID for functional enrichment analysis.

"We found that gene numbers and lengths had..." think the authors mean gene density, not gene number.

Response: Thank you for your comment.We have revised the language to reflect that we are discussing gene density, not gene number. Additionally, we have also changed "TE number" to "TE density" to ensure clarity and accuracy in our analysis. Thank you for bringing this to our attention.
