# Supplementary material for: Chromosome-level genome and recombination map of the male buffalo
Source: Gigascience. 2023 Aug 17;12:giad063. doi: 10.1093/gigascience/giad063 (PMC10433102; doi:10.1093/gigascience/giad063)
Supplement: giad063_Supplemental_File [file giad063_supplemental_file.docx]

**
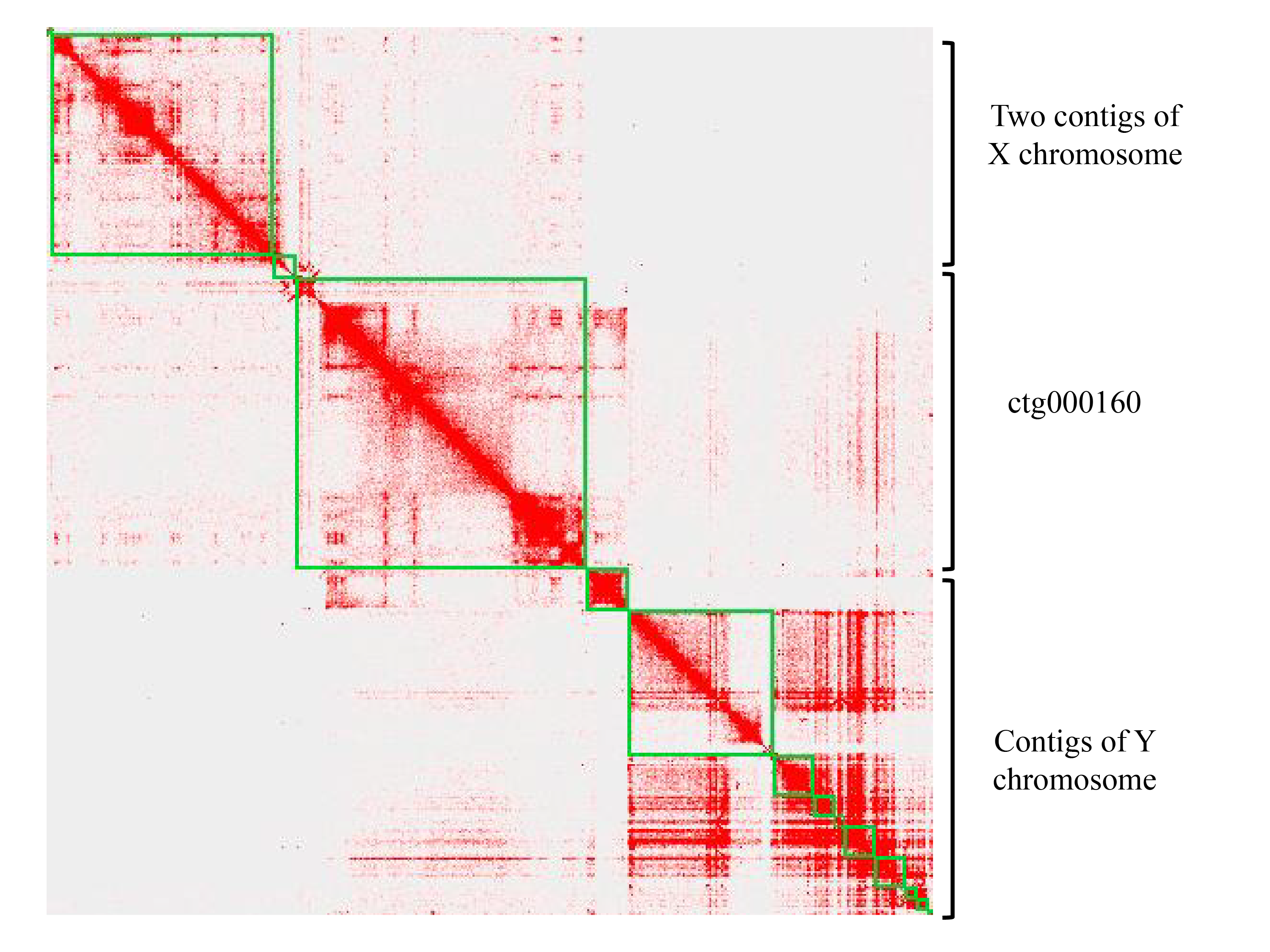
**

**Figure S1.** The interaction between the candidate contig ctg000160 in the PAR region and the contigs of the X and Y chromosomes.

**
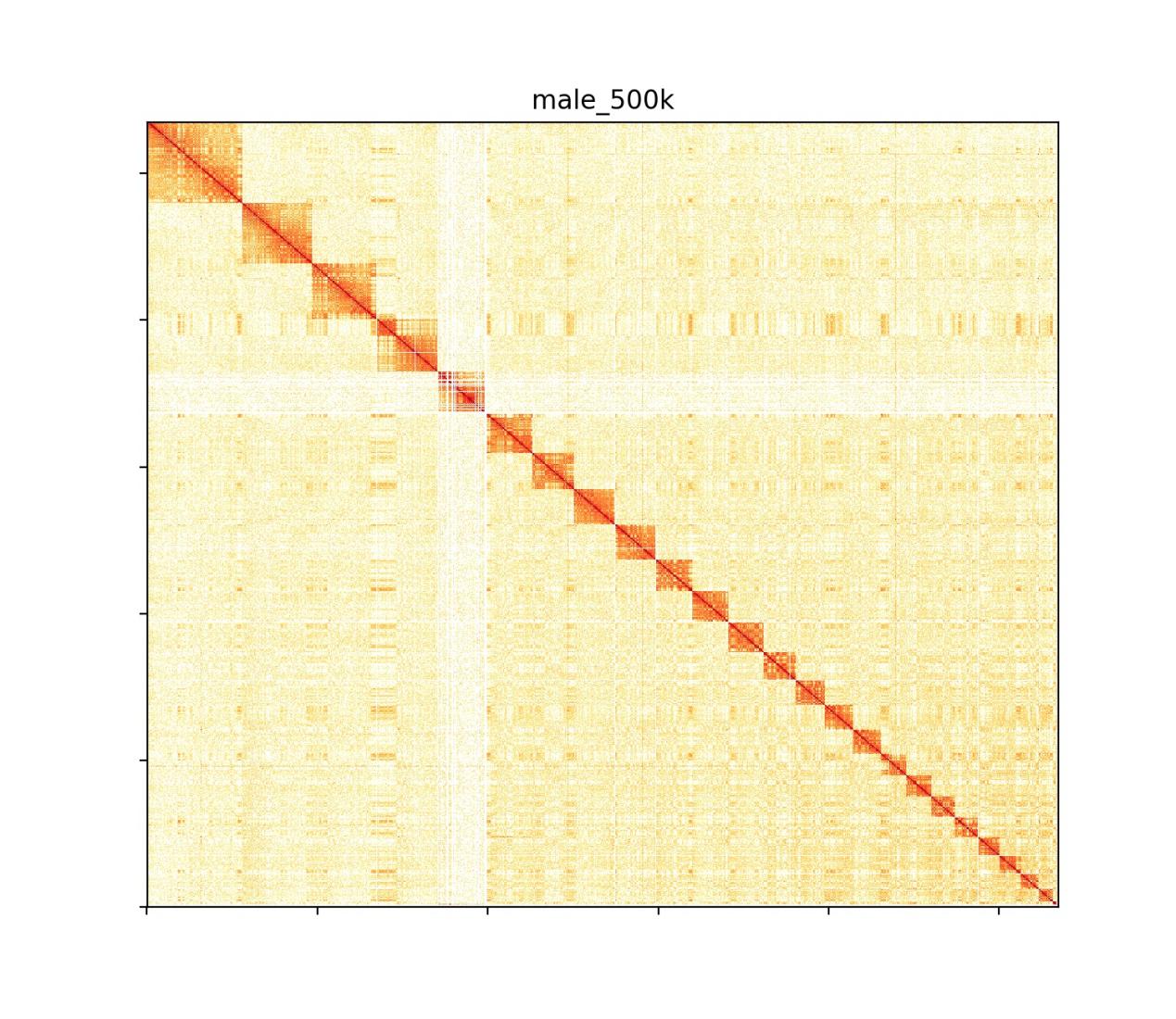
**

**Figure S2.** The heatmap (resolution: 500kb) of the male buffalo genome. The increase in interaction signal is represented from yellow to red color.


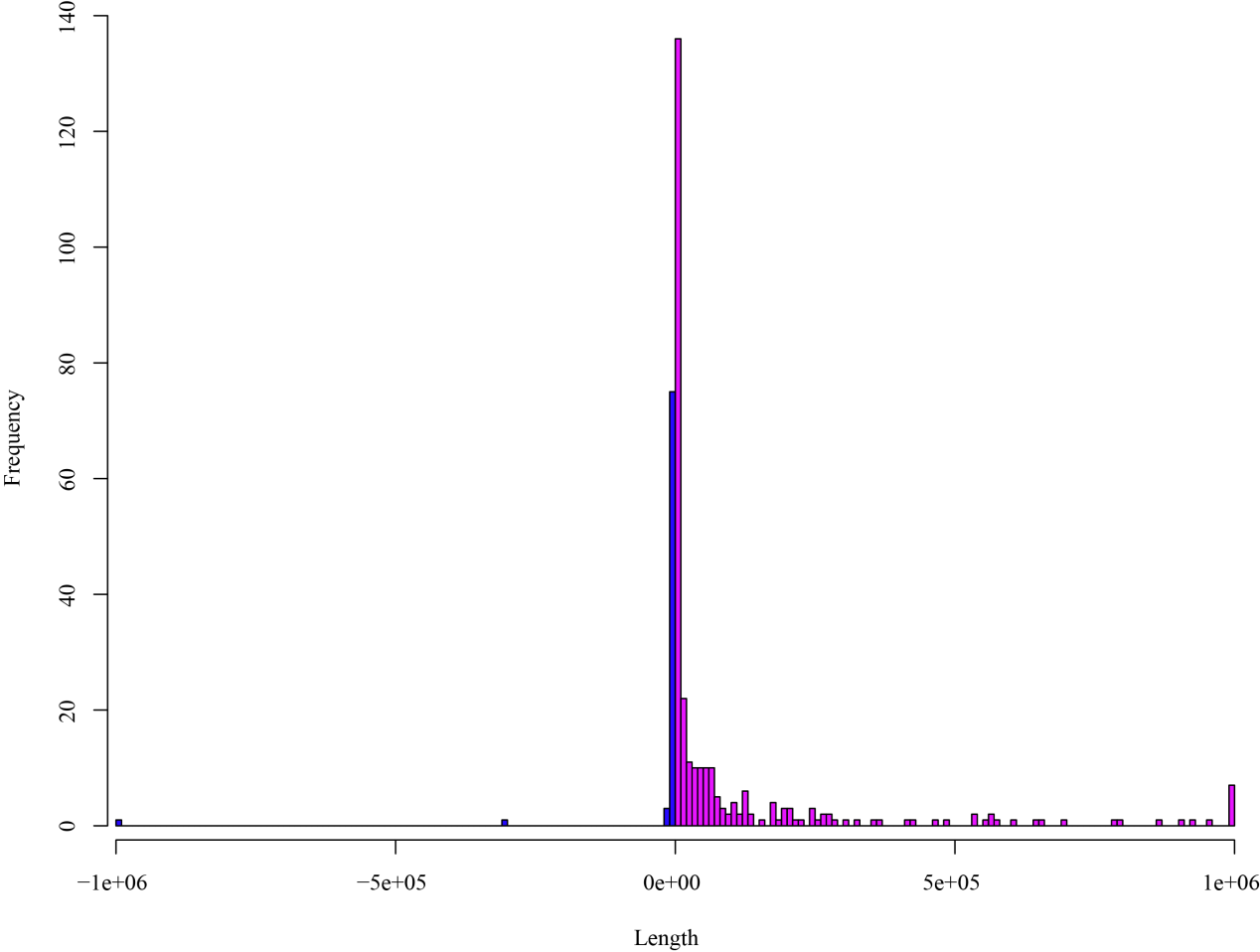


**Figure S3.** The distribution of closed gap lengths (bin size 5kb). We extracted the 5kb sequences at both ends of the gaps and aligned them to the male buffalo genome to determine the positions and lengths of the gaps. The true gaps are colored in pink (size >=0bp), while the false gaps are colored in blue (size <0bp). False gap sizes indicate that the sequences at both ends of gaps are in opposite directions in male and female buffalo, and the two sides of gaps overlap in female buffalo.


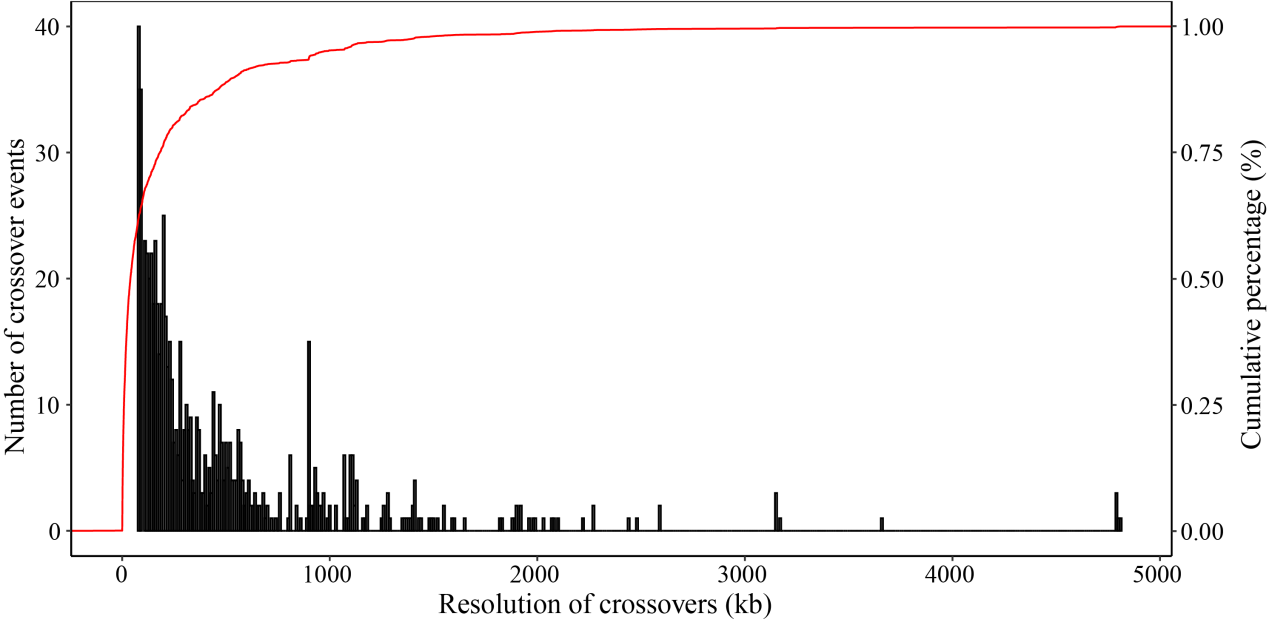


**Figure S4.** Distribution of the internal sizes. The red line indicates the cumulative proportion


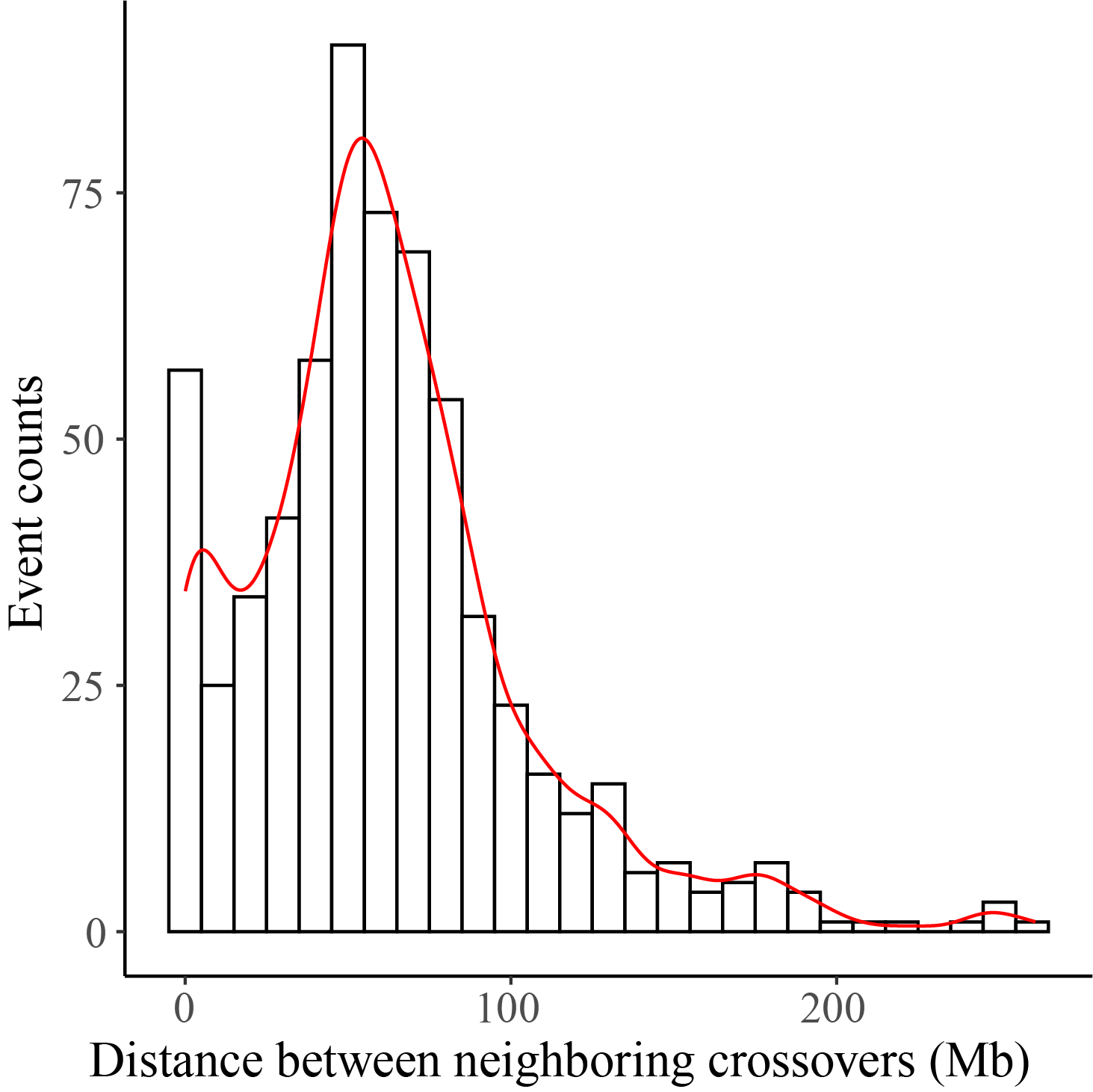


**Figure S5.** Distribution of distances between adjacent recombinations.


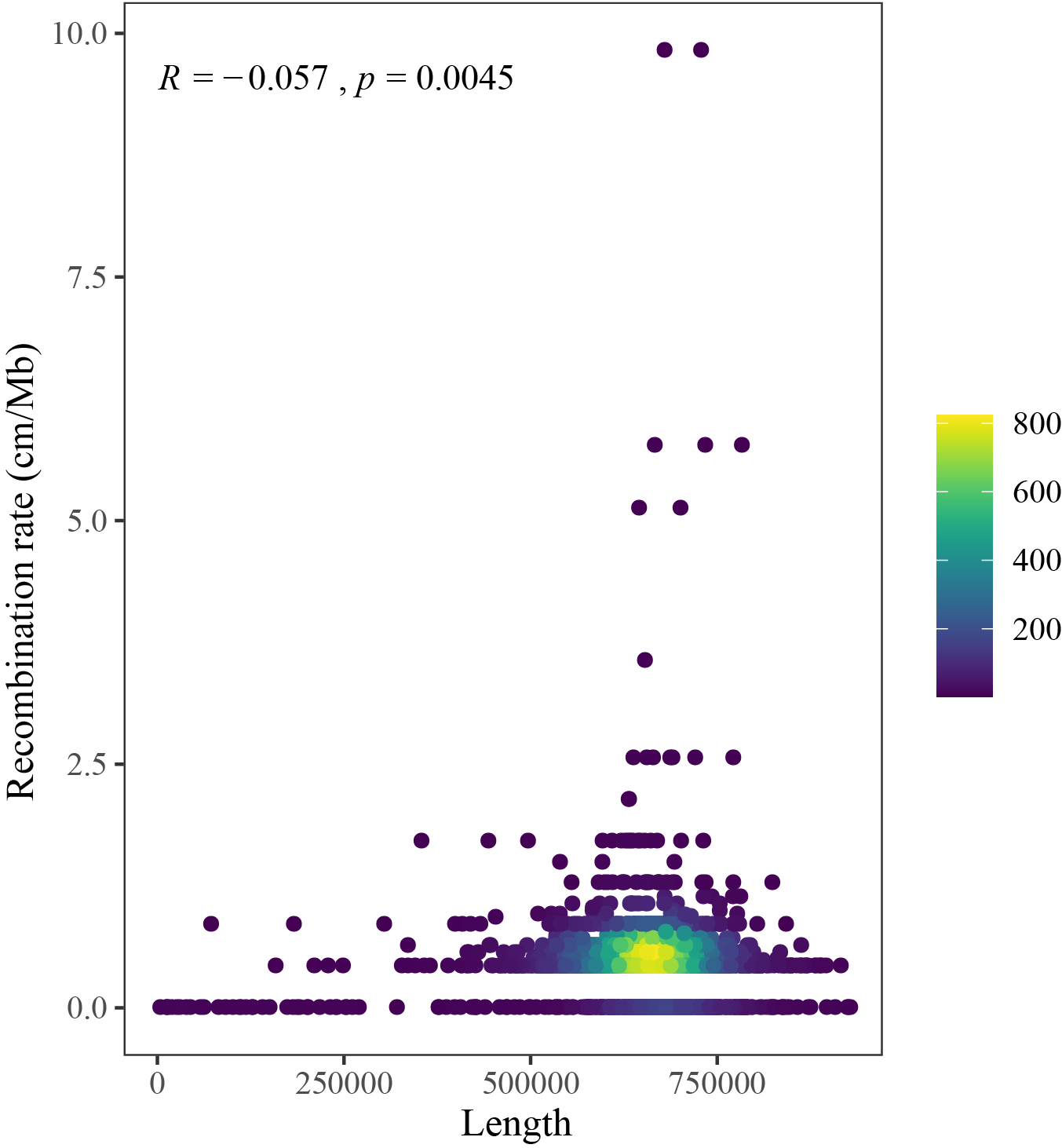


**Figure S6.** Correlation between SV length and recombination rate.


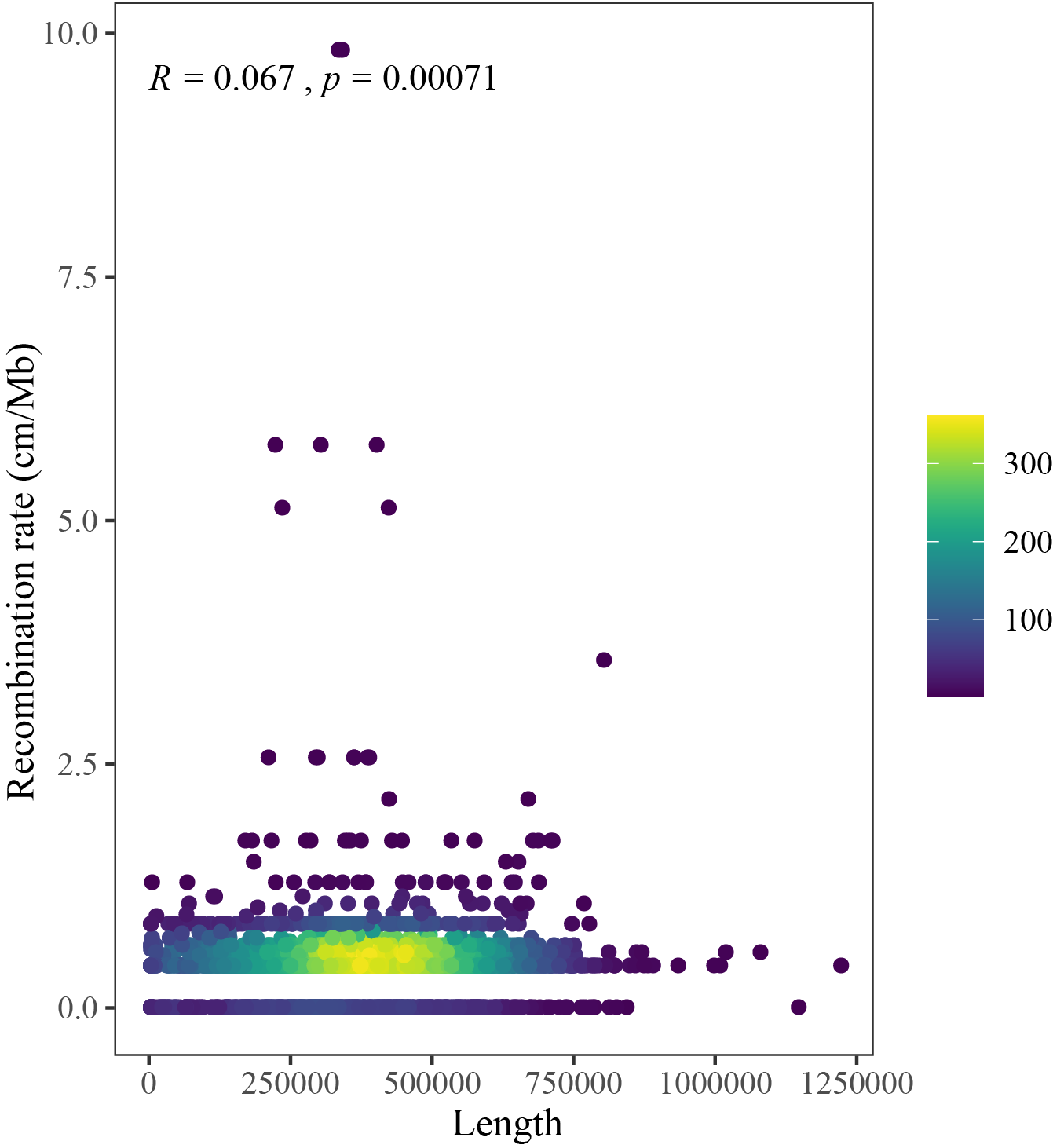


**Figure S7.** Correlation between gene length and recombination rate.

| **Approach** | | **Gene number** | **Total CDS length (Mb)** | **Average CDS length (bp)** | **Average exons per gene** | **Average exon length (bp)** | **Average intron length (bp)** |
| --- | --- | --- | --- | --- | --- | --- | --- |
| **De novo** | Augustus | 21,695 | 33.22 | 1,531 | 8.85 | 173.03 | 5,920 |
|  | SNAP | 148,110 | 207.50 | 1,400 | 9.77 | 143.43 | 1,614 |
|  | GlimmerHMM | 110,722 | 72.55 | 655 | 2.92 | 224.14 | 4,702 |
|  | Genscan | 41,531 | 56.74 | 1,366 | 8.05 | 169.73 | 6,115 |
| **Homology** | Human | 19,961 | 31.48 | 1,577 | 8.81 | 179.00 | 3,468 |
|  | Mouse | 18,109 | 27.49 | 1,518 | 8.30 | 182.88 | 3,612 |
|  | Cow | 23,035 | 34.14 | 1,482 | 7.81 | 189.66 | 3,670 |
|  | Sheep | 20,761 | 31.09 | 1,497 | 8.64 | 173.43 | 3,333 |
|  | Horse | 19,127 | 30.92 | 1,616 | 8.49 | 190.37 | 3,704 |
| **Transcriptome** | StringTie | 61,522 | 241.93 | 3,932 | 11.63 | 337.98 | 5,743 |
| **EVM** |  | 22,608 | 36.49 | 1,614 | 9.30 | 173.52 | 4,701 |

**Table S1.** Statistics of predicted protein-coding genes in the male buffalo genome.

|  | **TEs** | |
| --- | --- | --- |
|  | **Length** | **Ratio** |
| **DNA** | 74,472,647 | 2.78% |
| **LINE** | 979,067,036 | 36.60% |
| **LTR** | 135,165,291 | 5.05% |
| **SINE** | 127,909,601 | 4.78% |
| **Unknown** | 2,587,646 | 0.10% |
| **Others** | 2,258,545 | 0.08% |
| **Total** | 1,321,460,766 | 49.39% |

**Table S2.** Analysis of transposable elements (TEs) in the male buffalo genome.

| **Category** | **Term** | **Count** | **P-value** |
| --- | --- | --- | --- |
| GOTERM_BP_DIRECT | GO:0031214~biomineral tissue development | 3 | 5.6E-05 |
| UP_KW_BIOLOGICAL_PROCESS | KW-0091~Biomineralization | 3 | 8.2E-04 |
| UP_SEQ_FEATURE | MOTIF:Cell attachment site | 3 | 4.8E-03 |
| BIOCARTA | h_npp1Pathway:Regulators of Bone Mineralization | 2 | 6.2E-03 |
| GOTERM_MF_DIRECT | GO:0005515~protein binding | 22 | 6.8E-03 |
| GOTERM_MF_DIRECT | GO:0051371~muscle alpha-actinin binding | 2 | 1.8E-02 |
| UP_SEQ_FEATURE | COMPBIAS:Polar residues | 14 | 2.9E-02 |
| UP_KW_LIGAND | KW-0730~Sialic acid | 2 | 3.4E-02 |

**Table S3.** Functional enrichment of genes around recombination hotspots.

| **Platform** | **Number** | **Length (Gb)** | **Coverage** |
| --- | --- | --- | --- |
| Illumina | 3,144,762,308 | 466.1 | 174X |
| Nanopore | 17,326,651 | 271.9 | 102X |
| Bionano | 2,729,520 | 561.4 | 210X |
| HiC | 2,963,360,680 | 291.8 | 109X |

**Table S4.** Statistics of datasets for genome assembly.

| **Sample** | **Number** | **Length (Gb)** | **Coverage** | **Sample** | **Number** | **Length (Gb)** | **Coverage** |
| --- | --- | --- | --- | --- | --- | --- | --- |
| sperm01 | 112,026,336 | 16.8 | 6X | sperm40 | 120,585,192 | 18.1 | 7X |
| sperm02 | 110,057,974 | 16.5 | 6X | sperm41 | 119,422,182 | 17.9 | 7X |
| sperm03 | 122,419,966 | 18.4 | 7X | sperm42 | 121,295,520 | 18.2 | 7X |
| sperm04 | 112,759,848 | 16.9 | 6X | sperm43 | 128,717,992 | 19.3 | 7X |
| sperm05 | 101,435,476 | 15.2 | 6X | sperm44 | 105,225,692 | 15.8 | 6X |
| sperm06 | 118,623,082 | 17.8 | 7X | sperm45 | 132,638,884 | 19.9 | 7X |
| sperm07 | 109,142,508 | 16.4 | 6X | sperm46 | 121,711,178 | 18.3 | 7X |
| sperm08 | 112,437,328 | 16.9 | 6X | sperm47 | 119,963,082 | 18.0 | 7X |
| sperm09 | 105,069,234 | 15.8 | 6X | sperm48 | 120,219,046 | 18.0 | 7X |
| sperm10 | 133,246,324 | 20.0 | 7X | sperm49 | 125,689,252 | 18.9 | 7X |
| sperm11 | 106,754,394 | 16.0 | 6X | sperm50 | 128,368,238 | 19.3 | 7X |
| sperm12 | 126,384,732 | 19.0 | 7X | sperm51 | 147,613,764 | 22.1 | 8X |
| sperm13 | 131,951,620 | 19.8 | 7X | sperm52 | 143,972,316 | 21.6 | 8X |
| sperm14 | 121,554,022 | 18.2 | 7X | sperm53 | 119,578,764 | 17.9 | 7X |
| sperm15 | 126,780,970 | 19.0 | 7X | sperm54 | 115,555,952 | 17.3 | 6X |
| sperm16 | 124,217,800 | 18.6 | 7X | sperm55 | 111,967,830 | 16.8 | 6X |
| sperm17 | 121,603,856 | 18.2 | 7X | sperm56 | 116,176,858 | 17.4 | 7X |
| sperm18 | 131,273,684 | 19.7 | 7X | sperm57 | 149,119,240 | 22.4 | 8X |
| sperm19 | 132,683,254 | 19.9 | 7X | sperm58 | 85,687,110 | 12.9 | 5X |
| sperm20 | 136,775,018 | 20.5 | 8X | sperm59 | 115,828,514 | 17.4 | 6X |
| sperm21 | 124,241,526 | 18.6 | 7X | sperm60 | 94,144,130 | 14.1 | 5X |
| sperm22 | 135,113,170 | 20.3 | 8X | sperm61 | 115,100,830 | 17.3 | 6X |
| sperm23 | 131,718,378 | 19.8 | 7X | sperm62 | 111,799,100 | 16.8 | 6X |
| sperm24 | 127,450,410 | 19.1 | 7X | sperm63 | 119,146,890 | 17.9 | 7X |
| sperm25 | 157,087,146 | 23.6 | 9X | sperm64 | 119,300,544 | 17.9 | 7X |
| sperm26 | 138,385,394 | 20.8 | 8X | sperm65 | 119,347,878 | 17.9 | 7X |
| sperm27 | 150,245,800 | 22.5 | 8X | sperm66 | 117,496,332 | 17.6 | 7X |
| sperm28 | 154,103,850 | 23.1 | 9X | sperm67 | 152,733,574 | 22.9 | 9X |
| sperm29 | 104,295,380 | 15.6 | 6X | sperm68 | 128,230,340 | 19.2 | 7X |
| sperm30 | 144,073,542 | 21.6 | 8X | sperm69 | 116,026,218 | 17.4 | 7X |
| sperm31 | 132,475,998 | 19.9 | 7X | sperm70 | 129,014,400 | 19.4 | 7X |
| sperm32 | 121,546,344 | 18.2 | 7X | sperm71 | 176,135,816 | 26.4 | 10X |
| sperm33 | 126,786,906 | 19.0 | 7X | sperm72 | 124,596,164 | 18.7 | 7X |
| sperm34 | 129,671,728 | 19.5 | 7X | sperm73 | 130,325,124 | 19.5 | 7X |
| sperm35 | 121,211,476 | 18.2 | 7X | sperm74 | 107,882,892 | 16.2 | 6X |
| sperm36 | 120,802,208 | 18.1 | 7X | sperm75 | 118,665,368 | 17.8 | 7X |
| sperm37 | 140,520,022 | 21.1 | 8X | sperm76 | 124,722,308 | 18.7 | 7X |
| sperm38 | 151,686,446 | 22.8 | 9X | sperm77 | 148,839,252 | 22.3 | 8X |
| sperm39 | 145,474,762 | 21.8 | 8X | sperm78 | 121,864,808 | 18.3 | 7X |

**Table S5.** Statistics of 78 sperms in male buffalo.

| **Tissue** | **Number** | **Length (Gb)** | **Coverage** |
| --- | --- | --- | --- |
| dorsal muscle | 35,601,646 | 5.3 | 146X |
| lung | 41,650,100 | 6.2 | 170X |
| liver | 42,665,088 | 6.4 | 175X |
| spleen | 43,484,510 | 6.5 | 178X |
| tongue | 49824848 | 7.4 | 204X |
| kidney | 36,971,846 | 5.5 | 151X |
| heart | 41,165,732 | 6.1 | 168X |
| hind leg | 37,726,560 | 5.6 | 154X |
| fore leg | 40,671,078 | 6.1 | 166X |
| adipose tissue | 41,190,228 | 6.1 | 168X |
| conarium | 42,904,208 | 6.4 | 175X |
| hypothalamus | 39,946,646 | 6.0 | 163X |
| cerebellum | 42,601,308 | 6.4 | 174X |
| medulla oblongata | 37,360,334 | 5.6 | 153X |
| BA7/20 | 45,309,066 | 6.8 | 185X |
| BA21/22/41/42 | 38,909,706 | 5.8 | 159X |
| BA23/31/35 | 74,363,296 | 11.1 | 304X |
| BA24/32 | 37,907,076 | 5.7 | 155X |
| BA43 | 39,394,286 | 5.9 | 161X |
| BA11/25 | 39,930,408 | 6.0 | 163X |
| BA44/45/46 | 36,732,986 | 5.5 | 150X |

**Table S6.** Summary of 21 transcriptome sequencing datasets.

| **Tissue** | **#SV-inserted genes showing highest expression** | **#genes showing highest expression** | **#SV-inserted genes** | **#genes in male swamp buffalo** | **p-value** |
| --- | --- | --- | --- | --- | --- |
| lung | 1,230 | 2,092 | 11,293 | 22,608 | 1.67E-05 |
| kindey | 1,086 | 1,912 | 11,293 | 22,608 | 0.001295 |
| heart | 535 | 904 | 11,293 | 22,608 | 0.002561 |
| liver | 436 | 755 | 11,293 | 22,608 | 0.01934 |
| foreleg | 406 | 700 | 11,293 | 22,608 | 0.0201 |
| adipose | 899 | 1,633 | 11,293 | 22,608 | 0.02543 |
| medulla | 398 | 695 | 11,293 | 22,608 | 0.03509 |
| spleen | 1,072 | 1,983 | 11,293 | 22,608 | 0.04825 |

**Table S7.** Tissue analysis of SV-inserted genes showing highest expression.

| **Tissue** | **#unique SV-inserted genes showing highest expression** | **#genes showing highest expression** | **#unique SV-inserted genes** | **#genes in male swamp buffalo** | **p-value** |
| --- | --- | --- | --- | --- | --- |
| lung | 520 | 2092 | 4243 | 22608 | 6.16E-08 |
| kidney | 440 | 1912 | 4243 | 22608 | 0.0002593 |
| medulla | 175 | 695 | 4243 | 22608 | 0.0007422 |
| adipose | 370 | 1633 | 4243 | 22608 | 0.001845 |
| heart | 216 | 904 | 4243 | 22608 | 0.002078 |
| muscle | 160 | 688 | 4243 | 22608 | 0.01845 |
| cerebellum | 256 | 1151 | 4243 | 22608 | 0.01858 |
| BA23/31/35 | 101 | 420 | 4243 | 22608 | 0.03103 |
| tongue | 138 | 597 | 4243 | 22608 | 0.0336 |

**Table S8.** Tissue analysis of unique SV-inserted genes showing highest expression.
