# Supplementary material for: Chromosome-level genome and recombination map of the male buffalo
Source: Gigascience. 2023 Aug 17;12:giad063. doi: 10.1093/gigascience/giad063 (PMC10433102; doi:10.1093/gigascience/giad063)
Supplement: giad063_Response_to_Reviewer_Comments_Original_Submission [file giad063_response_to_reviewer_comments_original_submission.pdf]

Author's Response To Reviewer Comments

Close

Dear editors and reviewers:

Thank you for your kind reply. We are very grateful for the reviewer's comments on our manuscript entitled "Chromosome-level genome and recombination map of the male buffalo" (Manuscript id: GIGA-D-22-00319). Indeed, your comments are very helpful in improving our manuscript. We have carefully studied your comments and tried our best to revise the manuscript to meet your approval. We think we have finished all concerns from reviewers. Here, the revisions are documented in the revised manuscript, response letter, and Supplementary Figs and Tables. The responses to the reviewers' comments are as follows.

Kind regards,

Qingyou Liu

E-mail address: qyliu-gene@fosu.edu.cn

Response to reviewers

Reviewer reports:
Reviewer #1: The authors have generated a new swamp buffalo genome and sperm sequencing data, which will undoubtedly be useful, but details are missing throughout the manuscript which makes it very hard to follow. For example in the methods they just say "To construct a high-quality genome of the male swamp buffalo, several platforms including Illumina, nanopore , Bionano and HiC were used to generate a bulk of datasets" but dont discuss details such as read numbers, bionano and HiC technologies used etc. This is the same throughout the manuscript with lacking details. What was the coverage of each RNA-seq library? Each sequenced sperm? In results section is the same for example say "We found that these SV-inserted genes most tended to have the highest expression levels in the tissue (P=1.7E-05)". Specifically what genes and how many? How is the P value calculated? What is it being compared to? "We aligned the two haplotypes on the X chromosome of female swamp buffalo to determine" their locations. Which female genome? "The homozygous single nucleotide polymorphism (SNP) ratio is approximately 3.39 x 10-6 estimated on genomic short-read alignment" How was this determined? Assuming not from the transcript data? What is the units?
Response: We appreciate your time and constructive suggestions, which is very helpful for improving our manuscript. We apologize for the lack of details provided in the methods section and throughout the manuscript.We hope our revisions in this version could meet your criteria and further receive your replies. We will response to your comments point to point.
Q1: "To construct a high-quality genome of the male swamp buffalo, several platforms including Illumina, nanopore , Bionano and HiC were used to generate a bulk of datasets" but dont discuss details such as read numbers, bionano and HiC technologies used etc.
R1: We provide the statistics of these genomic data in Table S4, and describe the function of bionano and HiC technologies in "Sample collection and sequencing" of the Method sanction with the sentence "We generated about 466.1Gb (174X) Illumina short reads, 271.9Gb (102X) nanopore long reads, 561.4Gb (210X) bionano molecules and 291.8Gb (109X) HiC data (Table S4). The HiC data was used to scaffold the primary genome assembly, and Bionano data was further used to manually check the order and orientation". We further extend the sentence in "Genome assembly" sanction of Method with "we used 3d-dna (v180922) [44] with HiC data to anchor the contigs and manually adjust their orders in Juicebox as well as check with Bionano data for generating a chromosome-level genome."

Platform Number Length (Gb) Coverage

Illumina 3,144,762,308 466.1 174X
Nanopore 17,326,651 271.9 102X
Bionano 2,729,520 561.4 210X
HiC 2,963,360,680 291.8 109X
Table S4. Statistics of datasets for genome assembly.

Q2: What was the coverage of each RNA-seq library? Each sequenced sperm?
R2: Summary of RNA-seq and sperm sequencing are now listed in Table S4 and S5 separately.

Q3: In results section is the same for example say "We found that these SV-inserted genes most tended to have the highest expression levels in the tissue (P=1.7E-05)". Specifically what genes and how many? How is the P value calculated? What is it being compared to?
R3: We feel sorry that the tissue "lung" is missing. Now we correct it with the sentence "We found that genes with SV insertions tended to have the highest expression levels in the lung tissue (P=1.7E-05)". SV-inserted gene means the gene body contains structure variations (SVs) which are detected by comparing cow and swamp buffalo genome. We totally found 11,293 SV-inserted genes. We use chi-square test to calculate the P value using the total male swamp genes (22,608) as background for comparison. For example, we identified 1,230 SV-inserted genes showing highest expression in lung, and 2,092 of all the swamp genes showing highest expression in lung. The matrix (1230, 2092, 11293, 22608) was used to calculate P-value with chisq.test function in the R package. We add the method in "Detection of SVs" sanction. The results are showed in Table S7 and S8.

Q4: We aligned the two haplotypes on the X chromosome of female swamp buffalo to determine" their locations. Which female genome?
R4: The female genome is from Luo et al. (2020). We add the reference now.

Q5: "The homozygous single nucleotide polymorphism (SNP) ratio is approximately 3.39 x 10-6 estimated on genomic short-read alignment" How was this determined? Assuming not from the transcript data? What is the units?
R5: The short-read data of the reference genome, not the transcript data, was aligned on genome using BWA, and samtools and bcftools were used to call SNPs. We detected 9,070 homozygous SNP loci, which represents potential single base errors. The ratio is used to assess the single-base error rate of the genome and is calculated by dividing the number of homozygous SNPs by the genome size (2,675,361,621bp).

These are just some examples. The lack of details makes reviewing the manuscript at present very difficult as often not clear what exactly has been done. Maybe sometimes the details are found in other sections (though generally I couldn't find them) but even if so readers shouldn't have to keep switching between sections just to understand what was done. So think the analyses need to be properly written up and described before the paper can be properly reviewed.
Response: Thank you for your additional comments. We appreciate your feedback and understand the importance of providing sufficient details in the manuscript for readers to fully understand the analyses conducted. Following your and other reviewers' suggestion, we believe the manuscript has been improved, especially in the Method sanction.

Some more general comments:

"Furthermore, the absence of male swamp-buffalo genome hinders the detection of meiotic recombination" - Not sure that is true? Can map recombination events in female offspring or using male genotype data. Unless mean specifically on the Y chromosome?
Response: Thank you for your comment. You are correct that the absence of a male swamp-buffalo genome does not necessarily hinder the detection of meiotic recombination events in female offspring or using male genotype data. However, we specifically meant the detection of recombination events on the Y chromosome in male swamp-buffalo, which is not possible without a high-quality male genome. We have revised the manuscript to make this point more clear with the sentence "Furthermore, the absence of male swamp-buffalo genome hinders the detection of sperm meiotic recombination on the Y chromosome". Thank you for bringing this to our attention and helping us improve the clarity of the manuscript.

"expect for chromosomes 1-5" - should be except
Response: Corrected.

"Recombination hotspots play an important role in shaping recombination landscapes" - Is that not just the same thing?
Response: Thank you for your comment. You are correct that the phrase "recombination hotspots" and "recombination landscapes" is redundant. We revise the sentence to clarify the role of recombination hotspots in assuring the orderly segregation of meiotic chromosomes as the reference [30] mentioned.

Why do the authors infer that genes being at recombination hotspots reflects an adaptive process. As they show many things shape recombination rates so would need to more clearly demonstrate a link than just some interesting genes are found at the recombination hotspots.
Response: We agree that the inference that genes being located at recombination hotspots reflects an adaptive process requires more evidence and clarification. We misinterpreted this result, and what we actually wanted to express was that recombination hotspots may provide diversity to these tooth-related genes, as mentioned in reference [30]. However, more experiments are needed to test this hypothesis. So, we correct the sentence "These genes related to tooth development around recombination hotspots may have contributed to better dietary adaptations in buffaloes" to "Recombination hotspots may provide genetic diversity to these tooth-related genes, but further experimental validation is required to confirm their functional roles".

Does the peak near 0 in Figure S5 suggest some false positive recombination events leading to nearby switches? If so should some filtering be applied?
Response: Thank you for your comment. The peak near 0 in Figure S5 may indeed suggest the presence of false positive recombination events or genotyping errors.
We did apply filtering criteria to reduce the impact of genotyping errors and false positive recombination events, such as removing SNPs with a low quality score and requiring a minimum number of reads for each SNP. However, we acknowledge that more stringent filtering criteria may be necessary to further reduce the impact of false positive events.
We revise the "SNP calling" and "Identifying recombination events in sperms" sanctions to provide a more detailed description of our filtering criteria and acknowledge the potential false positives on our results. Thank you for bringing this to our attention and helping us improve the accuracy of the manuscript.

What is the cause of the faint band in Figure S2?
Response: The weak band corresponds to the X chromosome, which indicates that the X chromosome interacts weakly with the other chromosomes of the male swamp buffalo. This phenomenon is also observed in Luo et al (see the HiC heatmaps below). The reasons behind this need to be studied in future.

Genome-wide all-by-all Hi-C interactions in female swamp (left) and river (right) buffalo.

Reviewer #2: The manuscript "Chromosome-level genome and recombination map of the male buffalo" by Wang and coauthors describes the accurate genome reconstruction of a male swamp buffalo. This information has great scientific and economic value in the livestock community.
Experimental techniques and computational methods adopted are adequate and well described and particularly useful to the reconstruction of the Y chromosome.
The manuscript also describes the first recombination map in buffalo, obtained by the whole genome sequencing of 78 sperms. Identification of hotspots located on chromosome 8 demonstrates the scientific potentiality of such result, showing how genes near these hotspots are related to tooth quality.

The manuscript is well written and can be accepted in its present form

Response: Thank you very much for your positive comments.

Reviewer #3: The study is well presented. The main problem found in the manuscript is related to some aspects of the methodology and typo errors or ambiguous phrases or sentences. It is necessary to improve

the manuscript by addressing the questions that need to be clarified in a better way.
Response: Thank you for taking the time to review our manuscript and for providing valuable feedback to help us improve the quality of the research. We carefully review the manuscript to address the unclear or ambiguous aspects and improve the clarity and accuracy of the presentation.

The Results and Discussion section needs to be more elaborate and include more references to support the findings.
Response: Thank you for your comment. We comprehensively polish the Results and Discussion section following your constructive suggestions (highlighted with blue colors). We also incorporate additional references to support our conclusions and ensure that our research is situated within the broader context of relevant literature in the field. Thank you for your valuable feedback and for helping us to improve the quality of our manuscript.

The use of different tissues and the distribution part has to be discussed much better.
Response: The use of different tissues and the distribution part have been discussed in our published paper (below), and we have added this reference in our manuscript. Thank you for your reminder.

Xiaobo, W., Hassan, F. U., Liu, S. et al. De Novo Transcriptome Dataset Generation of the Swamp Buffalo Brain and Non-Brain Tissues. BioMed Research International, 2022.

An ethics statement needs to be included to confirm that your named ethics committee specifically approved this study.
Response: Thank you for your comment. We agree that an ethics statement is an important aspect of any research study, and we have included a clear statement regarding the approval of the study by the appropriate ethics committee.

Some important questions should be also addressed and integrated in the manuscript such as:
What is the application aspect of the study?
Response: The main application of our study is to improve our understanding of the genetic mechanisms underlying important traits in swamp buffalo, which can help to facilitate the breeding of more productive and disease-resistant animals.

How would it be applicable to daily life of farmers?
Response: The study has the potential to impact the daily lives of farmers through its contributions to the breeding of water buffaloes for meat and milk production. By identifying genetic variations related to desirable traits and using this information in breeding programs, farmers can improve the productivity and profitability of their herds.

Which sector it covers the most?
Response: Our study could have significant implications for the agricultural sector, particularly in regions where swamp buffalo are an important livestock resource. Our research may also have broader implications for the study of genome evolution and recombination, which can provide insights into the genetic mechanisms that drive species diversification and adaptation.

What is the future of your research?
Response: The future of the research includes the construction of the genome and recombination map of male river buffalo, which will allow for further comparisons and insights into the domestication features of the two sub-species. Additionally, further functional assays need to be performed to characterize the binding motif of swamp buffalo PRDM9, which may lead to a better understanding of the factors affecting recombination distribution. We plan to continue investigating the genetic basis of important traits in swamp buffalo, and to explore ways to use this information to improve breeding programs and animal welfare. We also hope to collaborate with other researchers to develop new technologies and methodologies for studying the genetics of non-model organisms.

Close
