# Supplementary material for: Chromosome-level genome and recombination map of the male buffalo
Source: Gigascience. 2023 Aug 17;12:giad063. doi: 10.1093/gigascience/giad063 (PMC10433102; doi:10.1093/gigascience/giad063)
Supplement: giad063_Response_to_Reviewer_Comments_Revision_1 [file giad063_response_to_reviewer_comments_revision_1.pdf]

Author's Response To Reviewer Comments

Close

Reviewer #1: Thanks to the authors for their response. The manuscript looks good but I think there still are a couple of minor revisions needed. One is there are quite a few English errors so I have attached a tracked version of the manuscript where I have tried to fix/flag some of these in case useful. But think it could likely benefit from some further editing.
Response: Thank you for your feedback and suggestions. We have carefully reviewed and revised the manuscript according to your comments. We have also paid close attention to the English language errors and made necessary corrections. We appreciate your help in improving the quality of our manuscript.

Regarding the details of the sequencing technologies used. It is good to see the details the authors have added. But they still, for example, havnt specified things such as what HiC technology was used (Dovetail?), how was sequenced (Illumina? Read length?). Also for example was it Bionano Saphyr? I think this kind of information should be included in a genome assembly paper.
Response: Thank you for your comment. We apologize for any confusion caused by the lack of clarity in our manuscript. Illumina Hi-C technology was used in this study. For the construction of Hi-C libraries, the buffalo DNA was digested with the restriction enzyme MboI and then was sequenced on a Novoseq 6000 platform with PE100 reads. Bionano Saphyr technology was applied and DLE1 restriction enzyme was used for digestion. We have update our manuscript to include this information.

Also references are missing e.g. at line "Although several of female buffalo genomes have been finished " but they dont cite any. Or for example say "Besides, about 92% of the annotated Y genes in the bull genome could be explicitly..." and "which is well mapped by 92% of the annotated genes in the bull Y genome" but dont specify which bull or which annotations. Or where say "which exhibits better contiguity than published buffalo genomes ". But none cited.
Response:Thank you for your valuable feedback on our manuscript. We apologize for the oversight in not including the necessary references in the mentioned sections. We have now revised the manuscript and included the appropriate citations.

Authors still dont say what this number represents "The homozygous single nucleotide polymorphism (SNP) ratio was approximately 3.39 x 10- 6 based on genomic short-read alignment". From the author response it sounds like this is homozygous variant calls per basepair. If so the authors should specify this (or whatever other unit it is if this is not correct).
Response:Yes, the number represents the homozygous variant calls per basepair.We have revised the sentence to read as follows: 'The homozygous single nucleotide polymorphism (SNP) ratio was approximately 3.39 x 10- 6 per basepair based on genomic short-read alignment.'

"We mapped both swamp and river buffalo to the cattle reference genome and used Assemblytics to detect SVs ". If are talking about differences between species I dont think can refer to them as SVs. The term SV is typically used to refer to variants within a species, not across them. (including this is how is defined on wikipedia https://en.wikipedia.org/wiki/Structural_variation). Here seem to be referring to what are likely fixed genomic differences.
Response:Thank you for the suggestion. While the term "SV" is typically used to describe variants within a species, some studies do use it to describe genomic differences between different species. For example, Li et.al have aligned the gayal genome to the cattle genome to identify SVs. Therefore, in our study, we have also used the term "SV" to describe the genomic differences between swamp and river buffalo and cattle. However, we appreciate the reviewer's suggestion and will emphasize the point in the Method in our article.
Li, Y., Wang, S., Zhang, Z., Luo, J., Lin, G. L., Deng, W. D., et al. (2023). Large-scale chromosomal changes lead to genome-level expression alterations, environmental adaptation, and speciation in the Gayal (Bos frontalis). Molecular Biology and Evolution, 40(1), msad006.

"In addition, we identified an average of 69.2 PRDM9 binding motif (CCnCCnTnnCCnC) per Mb around crossovers ." How does this compare to regions not around crossovers? Otherwise figure is a bit meaningless.
Response: The density of PRDM9 binding motifs in the non-crossover regions is 66.3 per Mb. We have revised the description in the article to "Compared to non-crossover regions with a density of 66.3 PRDM9 binding motifs per Mb, we found a higher density of 69.2 binding motifs per Mb around crossovers, indicating a potential role of PRDM9 in regulating meiotic recombination hotspots."

"to perform functional analysis for candidate genes under a current background (Homo sapiens) with the Fisher's exact test" should you not restrict the background to genes with an orthologue in buffalo.
Response:DAVID requires selecting a species as background for functional enrichment analysis. As human genes have been studied more extensively, we first identified the human orthologs of the buffalo genes and then used human as the background species in DAVID for functional enrichment analysis.

"We found that gene numbers and lengths had..." think the authors mean gene density, not gene number.
Response: Thank you for your comment.We have revised the language to reflect that we are discussing gene density, not gene number. Additionally, we have also changed "TE number" to "TE density" to ensure clarity and accuracy in our analysis. Thank you for bringing this to our attention.

Close
