# Supplementary material for: Chromosome-level genome and recombination map of the male buffalo
Source: Gigascience. 2023 Aug 17;12:giad063. doi: 10.1093/gigascience/giad063 (PMC10433102; doi:10.1093/gigascience/giad063)
Supplement: giad063_Reviewer_1_Report_Original_Submission — James Prendergast -- 1/4/2023 Reviewed [file giad063_reviewer_1_report_original_submission.pdf]

**Reviewer Report**

**Title: Chromosome-level genome and recombination map of the male buffalo**

**Version: Original Submission** **Date:** 1/4/2023

**Reviewer name: James Prendergast**

**Reviewer Comments to Author:**

The authors have generated a new swamp buffalo genome and sperm sequencing data, which will undoubtedly be useful, but details are missing throughout the manuscript which makes it very hard to follow. For example in the methods they just say "To construct a high-quality genome of the male swamp buffalo, several platforms including Illumina, nanopore , Bionano and HiC were used to generate a bulk of datasets" but dont discuss details such as read numbers, bionano and HiC technologies used etc. This is the same throughout the manuscript with lacking details. What was the coverage of each RNA-seq library? Each sequenced sperm? In results section is the same for example say "We found that these SV-inserted genes most tended to have the highest expression levels in the tissue (P=1.7E-05)". Specifically what genes and how many? How is the P value calculated? What is it being compared to? "We aligned the two haplotypes on the X chromosome of female swamp buffalo to determine" their locations. Which female genome? "The homozygous single nucleotide polymorphism (SNP) ratio is approximately 3.39 x 10-6 estimated on genomic short-read alignment" How was this determined? Assuming not from the transcript data? What is the units?These are just some examples. The lack of details makes reviewing the manuscript at present very difficult as often not clear what exactly has been done. Maybe sometimes the details are found in other sections (though generally I couldn't find them) but even if so readers shouldn't have to keep switching between sections just to understand what was done. So think the analyses need to be properly written up and described before the paper can be properly reviewed.Some more general comments:"Furthermore, the absence of male swamp-buffalo genome hinders the detection of meiotic recombination" - Not sure that is true? Can map recombination events in female offspring or using male genotype data. Unless mean specifically on the Y chromosome?"expect for chromosomes 1-5" - should be except"Recombination hotspots play an important role in shaping recombination landscapes" - Is that not just the same thing?Why do the authors infer that genes being at recombination hotspots reflects an adaptive process. As they show many things shape recombination rates so would need to more clearly demonstrate a link than just some interesting genes are found at the recombination hotspots.Does the peak near 0 in Figure S5 suggest some false positive recombination events leading to nearby switches? If so should some filtering be applied?What is the cause of the faint band in Figure S2?

**Level of Interest**

Please indicate how interesting you found the manuscript: Choose an item.

**Quality of Written English**

Please indicate the quality of language in the manuscript: Choose an item.

**Declaration of Competing Interests**

Please complete a declaration of competing interests, considering the following questions:

- Have you in the past five years received reimbursements, fees, funding, or salary from an organisation that may in any way gain or lose financially from the publication of this manuscript, either now or in the future?
- Do you hold any stocks or shares in an organisation that may in any way gain or lose financially from the publication of this manuscript, either now or in the future?
- Do you hold or are you currently applying for any patents relating to the content of the manuscript?
- Have you received reimbursements, fees, funding, or salary from an organization that holds or has applied for patents relating to the content of the manuscript?
- Do you have any other financial competing interests?
- Do you have any non-financial competing interests in relation to this paper?

If you can answer no to all of the above, write 'I declare that I have no competing interests' below. If your reply is yes to any, please give details below.

I declare that I have no competing interests

I agree to the open peer review policy of the journal. I understand that my name will be included on my report to the authors and, if the manuscript is accepted for publication, my named report including any attachments I upload will be posted on the website along with the authors' responses. I agree for my report to be made available under an Open Access Creative Commons CC-BY license (http://creativecommons.org/licenses/by/4.0/). I understand that any comments which I do not wish to be included in my named report can be included as confidential comments to the editors, which will not be published.

Choose an item.

To further support our reviewers, we have joined with Publons, where you can gain additional credit to further highlight your hard work (see: https://publons.com/journal/530/gigascience). On publication of this paper, your review will be automatically added to Publons, you can then choose whether or not to claim your Publons credit. I understand this statement.

Yes Choose an item.
