# Supplementary material for: Chromosome-level genome and recombination map of the male buffalo
Source: Gigascience. 2023 Aug 17;12:giad063. doi: 10.1093/gigascience/giad063 (PMC10433102; doi:10.1093/gigascience/giad063)
Supplement: giad063_Reviewer_1_Report_Revision_1 — James Prendergast -- 4/26/2023 Reviewed [file giad063_reviewer_1_report_revision_1.pdf]

Reviewer Report

Title: Chromosome-level genome and recombination map of the male buffalo

Version: Revision 1 **Date:** 4/26/2023

Reviewer name: James Prendergast

Reviewer Comments to Author:

Thanks to the authors for their response. The manuscript looks good but I think there still are a couple of minor revisions needed. One is there are quite a few English errors so I have attached a tracked version of the manuscript where I have tried to fix/flag some of these in case useful. But think it could likely benefit from some further editing.Regarding the details of the sequencing technologies used. It is good to see the details the authors have added. But they still, for example, havnt specified things such as what HiC technology was used (Dovetail?), how was sequenced (Illumina? Read length?). Also for example was it Bionano Saphyr? I think this kind of information should be included in a genome assembly paper.Also references are missing e.g. at line "Although several of female buffalo genomes have been finished " but they dont cite any. Or for example say "Besides, about 92% of the annotated Y genes in the bull genome could be explicitly..." and "which is well mapped by 92% of the annotated genes in the bull Y genome" but dont specify which bull or which annotations. Or where say "which exhibits better contiguity than published buffalo genomes ". But none cited.Authors still dont say what this number represents "The homozygous single nucleotide polymorphism (SNP) ratio was approximately 3.39 x 10- 6 based on genomic short-read alignment". From the author response it sounds like this is homozygous variant calls per basepair. If so the authors should specify this (or whatever other unit it is if this is not correct)."We mapped both swamp and river buffalo to the cattle reference genome and used Assemblytics to detect SVs ". If are talking about differences between species I dont think can refer to them as SVs. The term SV is typically used to refer to variants within a species, not across them. (including this is how is defined on wikipedia https://en.wikipedia.org/wiki/Structural_variation). Here seem to be referring to what are likely fixed genomic differences."In addition, we identified an average of 69.2 PRDM9 binding motif (CCnCCnTnnCCnC) per Mb around crossovers ." How does this compare to regions not around crossovers? Otherwise figure is a bit meaningless."to perform functional analysis for candidate genes under a current background (Homo sapiens) with the Fisher's exact test" should you not restrict the background to genes with an orthologue in buffalo."We found that gene numbers and lengths had..." think the authors mean gene density, not gene number.

Level of Interest

Please indicate how interesting you found the manuscript: Choose an item.

Quality of Written English

Please indicate the quality of language in the manuscript: Choose an item.

Declaration of Competing Interests

Please complete a declaration of competing interests, considering the following questions:

- Have you in the past five years received reimbursements, fees, funding, or salary from an organisation that may in any way gain or lose financially from the publication of this manuscript, either now or in the future?
- Do you hold any stocks or shares in an organisation that may in any way gain or lose financially from the publication of this manuscript, either now or in the future?
- Do you hold or are you currently applying for any patents relating to the content of the manuscript?
- Have you received reimbursements, fees, funding, or salary from an organization that holds or has applied for patents relating to the content of the manuscript?
- Do you have any other financial competing interests?
- Do you have any non-financial competing interests in relation to this paper?

If you can answer no to all of the above, write 'I declare that I have no competing interests' below. If your reply is yes to any, please give details below.

I declare that I have no competing interests

I agree to the open peer review policy of the journal. I understand that my name will be included on my report to the authors and, if the manuscript is accepted for publication, my named report including any attachments I upload will be posted on the website along with the authors' responses. I agree for my report to be made available under an Open Access Creative Commons CC-BY license (http://creativecommons.org/licenses/by/4.0/). I understand that any comments which I do not wish to be included in my named report can be included as confidential comments to the editors, which will not be published.

Choose an item.

To further support our reviewers, we have joined with Publons, where you can gain additional credit to further highlight your hard work (see: https://publons.com/journal/530/gigascience). On publication of this paper, your review will be automatically added to Publons, you can then choose whether or not to claim your Publons credit. I understand this statement.

Yes Choose an item.
