# Supplementary material for: Chromosome-level genome and recombination map of the male buffalo
Source: Gigascience. 2023 Aug 17;12:giad063. doi: 10.1093/gigascience/giad063 (PMC10433102; doi:10.1093/gigascience/giad063)
Supplement: giad063_Reviewer_3_Report_Original_Submission — Dina El-Khishin -- 1/26/2023 Reviewed [file giad063_reviewer_3_report_original_submission.pdf]

Reviewer Report

Title: Chromosome-level genome and recombination map of the male buffalo

Version: Original Submission **Date:** 1/26/2023

Reviewer name: Dina El-Khishin

Reviewer Comments to Author:

The study is well presented. The main problem found in the manuscript is related to some aspects of the methodology and typo errors or ambiguous phrases or sentences. It is necessary to improve the manuscript by addressing the questions that need to be clarified in a better way.The Results and Discussion section needs to be more elaborate and include more references to support the findings.The use of different tissues and the distribution part has to be discussed much better.An ethics statement needs to be included to confirm that your named ethics committee specifically approved this study.Some important questions should be also addressed and integrated in the manuscript such as:What is the application aspect of the study?How would it be applicable to daily life of farmers?Which sector it covers the most?What is the future of your research?

Level of Interest

Please indicate how interesting you found the manuscript: Choose an item.

Quality of Written English

Please indicate the quality of language in the manuscript: Choose an item.

Declaration of Competing Interests

Please complete a declaration of competing interests, considering the following questions:

- Have you in the past five years received reimbursements, fees, funding, or salary from an organisation that may in any way gain or lose financially from the publication of this manuscript, either now or in the future?
- Do you hold any stocks or shares in an organisation that may in any way gain or lose financially from the publication of this manuscript, either now or in the future?
- Do you hold or are you currently applying for any patents relating to the content of the manuscript?
- Have you received reimbursements, fees, funding, or salary from an organization that holds or has applied for patents relating to the content of the manuscript?
- Do you have any other financial competing interests?
- Do you have any non-financial competing interests in relation to this paper?

If you can answer no to all of the above, write 'I declare that I have no competing interests' below. If your reply is yes to any, please give details below.

I declare that I have no competing interests

I agree to the open peer review policy of the journal. I understand that my name will be included on my report to the authors and, if the manuscript is accepted for publication, my named report including any attachments I upload will be posted on the website along with the authors' responses. I agree for my report to be made available under an Open Access Creative Commons CC-BY license (http://creativecommons.org/licenses/by/4.0/). I understand that any comments which I do not wish to be included in my named report can be included as confidential comments to the editors, which will not be published.

Choose an item.

To further support our reviewers, we have joined with Publons, where you can gain additional credit to further highlight your hard work (see: https://publons.com/journal/530/gigascience). On publication of this paper, your review will be automatically added to Publons, you can then choose whether or not to claim your Publons credit. I understand this statement.

Yes Choose an item.
